# Global antibiotic consumption and usage in humans, 2000–18: a spatial modelling study

**DOI:** 10.1016/S2542-5196(21)00280-1

**Published:** 2021-11-12

**Authors:** Annie J Browne, Michael G Chipeta, Georgina Haines-Woodhouse, Emmanuelle P A Kumaran, Bahar H Kashef Hamadani, Sabra Zaraa, Nathaniel J Henry, Aniruddha Deshpande, Robert C Reiner, Nicholas P J Day, Alan D Lopez, Susanna Dunachie, Catrin E Moore, Andy Stergachis, Simon I Hay, Christiane Dolecek

**Affiliations:** a Big Data Institute, Li Ka Shing Centre for Health Information and Discovery, Nuffield Department of Medicine, University of Oxford, Oxford, UK; b Oxford Centre for Global Health Research, Centre for Tropical Medicine and Global Health, Nuffield Department of Medicine, University of Oxford, Oxford, UK; c School of Pharmacy and School of Public Health, University of Washington, Seattle, WA, USA; d Institute for Health Metrics and Evaluation, University of Washington, Seattle, WA, USA; e Department of Health Metrics Sciences, School of Medicine, University of Washington, Seattle, WA, USA; f Mahidol-Oxford Tropical Medicine Research Unit, Faculty of Tropical Medicine, Mahidol University, Bangkok, Thailand

## Abstract

**Background:**

Antimicrobial resistance (AMR) is a serious threat to global public health. WHO emphasises the need for countries to monitor antibiotic consumption to combat AMR. Many low-income and middle-income countries (LMICs) lack surveillance capacity; we aimed to use multiple data sources and statistical models to estimate global antibiotic consumption.

**Methods:**

In this spatial modelling study, we used individual-level data from household surveys to inform a Bayesian geostatistical model of antibiotic usage in children (aged <5 years) with lower respiratory tract infections in LMICs. Antibiotic consumption data were obtained from multiple sources, including IQVIA, WHO, and the European Surveillance of Antimicrobial Consumption Network (ESAC-Net). The estimates of the antibiotic usage model were used alongside sociodemographic and health covariates to inform a model of total antibiotic consumption in LMICs. This was combined with a single model of antibiotic consumption in high-income countries to produce estimates of antibiotic consumption covering 204 countries and 19 years.

**Findings:**

We analysed 209 surveys done between 2000 and 2018, covering 284 045 children with lower respiratory tract infections. We identified large national and subnational variations of antibiotic usage in LMICs, with the lowest levels estimated in sub-Saharan Africa and the highest in eastern Europe and central Asia. We estimated a global antibiotic consumption rate of 14·3 (95% uncertainty interval 13·2–15·6) defined daily doses (DDD) per 1000 population per day in 2018 (40·2 [37·2–43·7] billion DDD), an increase of 46% from 9·8 (9·2–10·5) DDD per 1000 per day in 2000. We identified large spatial disparities, with antibiotic consumption rates varying from 5·0 (4·8–5·3) DDD per 1000 per day in the Philippines to 45·9 DDD per 1000 per day in Greece in 2018. Additionally, we present trends in consumption of different classes of antibiotics for selected Global Burden of Disease study regions using the IQVIA, WHO, and ESAC-net input data. We identified large increases in the consumption of fluoroquinolones and third-generation cephalosporins in North Africa and Middle East, and south Asia.

**Interpretation:**

To our knowledge, this is the first study that incorporates antibiotic usage and consumption data and uses geostatistical modelling techniques to estimate antibiotic consumption for 204 countries from 2000 to 2018. Our analysis identifies both high rates of antibiotic consumption and a lack of access to antibiotics, providing a benchmark for future interventions.

**Funding:**

Fleming Fund, UK Department of Health and Social Care; Wellcome Trust; and Bill & Melinda Gates Foundation.

## Introduction

Bacterial infections are a major cause of morbidity and mortality worldwide. Antibiotics have been hugely successful in improving health outcomes, and alongside improvements in nutrition, clean water, sanitation, and vaccination provision, have aided in the global reduction of under-5 mortality from 216 deaths per 1000 livebirths in 1950 to 39 deaths per 1000 livebirths in 2017, and an increase in male life expectancy from 48 years to 71 years within the same time period.^1^, ^2^ The positive impact of antibiotics on health, however, is threatened by increasing levels of antimicrobial resistance (AMR) worldwide and hampered by the lack of access to essential antibiotics in many low-income and middle-income countries (LMICs).^3^, ^4^

The relationship between antibiotic use and the development and spread of AMR is well documented.^5^, ^6^, ^7^ A full understanding of the quantities and classes of antibiotics being used globally and in each geographical context is vital to inform national action plans aimed at promoting judicious antibiotic use and reducing the spread and further entrenchment of AMR.^8^

Sustainable Development Goal 3.8 specifies the need for “access to safe, effective, quality and affordable essential medicines and vaccines for all”.^9^ Limited access to antibiotics results in many bacterial infections going untreated, increasing morbidity and mortality,^3^ whereas suboptimal dosing and poor pharmaceutical quality contributes to the development and propagation of AMR.^10^ Improving access to antibiotics is vital to better health outcomes. Surveillance of antibiotic consumption is a crucial component among strategies to tackle AMR.^4^, ^11^

**Research in context**

**Evidence before this study**

Despite some excellent national and regional surveillance systems for antimicrobial consumption, mostly in high-income countries (HICs), large data gaps remain. This is compounded by the fact that the most comprehensive and detailed sources of global antibiotic sales data are proprietary databases. The IQVIA MIDAS database covers 76 countries or territories, including only one low-income country. To identify existing studies estimating global antibiotic consumption we searched PubMed from Jan 1, 1980, to July 28, 2020, for studies using the terms (“antimicrobial” OR “antibiotic”) AND (“consumption” OR “use” OR “usage”) AND (“global” OR “low-income” OR “middle-income” OR “high-income”) in the title, with no language restrictions.

We identified five studies that estimated global antibiotic consumption, four of these were published in the past 10 years and were based entirely on the IQVIA MIDAS database, with the two most recent studies following the WHO Anatomical Therapeutic Chemical (ATC) classification/defined daily doses (DDD) methodology. Additionally, several studies were identified that focused on analysing the consumption of child-appropriate oral formulations of antibiotics using the IQVIA MIDAS database. The most comprehensive study estimated global antibiotic consumption from 2000 to 2015 and calculated consumption for the 76 countries or territories covered by the dataset, extrapolating the rates of consumption to the global population. However, the study did not provide estimates of antibiotic consumption for individual countries not covered by the IQVIA dataset.

Our searches for global antibiotic usage identified one study estimating caregiver-reported antibiotic usage in children (aged <5 years) with fever, diarrhoea, or cough with difficulty breathing in low-income and middle-income countries (LMICs). The study used Demographic Health Survey and Multiple Indicator Cluster household data; estimates were stratified by WHO region. The study did not produce estimates of antibiotic usage for individual countries and districts.

**Added value of this study**

We provide the first estimates of longitudinal human antibiotic consumption (ATC J01) for 204 countries from 2000 to 2018, using a novel approach that applied spatial modelling techniques and incorporated multiple data sources, including large-scale antibiotic usage surveys. We used the most recent version of the WHO ATC/DDD guideline and index with higher DDD values for key antibiotics such as ampicillin and amoxicillin. We estimate that the global volume of antibiotic consumption was 40·2 (95% uncertainty interval 37·2-43·7) billion DDD in 2018, an increase of 46% since 2000. Antibiotic consumption in 2018 was inequitable between HICs and LMICs, with respective rates of 20·6 DDD and 13·1 DDD per 1000 population per day.

Rates of antibiotic consumption varied by nearly a factor of 10, highlighting the lack of access to essential antibiotics as well as overuse. We also present trends in consumption of different classes of antibiotics using the IQVIA, WHO, and European Surveillance of Antimicrobial Consumption Network (ESAC-Net) input data. Additionally, we provide detailed subnational estimates of community-based antibiotic usage in children with lower respiratory tract infections in LMICs (135 countries), from 2000 to 2018. Our study highlights the substantial variation in antibiotic usage between LMICs and the high inequalities within some countries.

**Implications of all the available evidence**

Insights from longitudinal antibiotic consumption surveillance within a country are essential for implementing and monitoring policy to tackle inappropriate antibiotic use. Addressing lack of access as well as inappropriate antibiotic use is crucial for tackling the growing threat of antimicrobial resistance and ensuring fair and equitable access to essential medicines for all.

Despite some excellent national and regional surveillance systems,^12^, ^13^ mainly in high-income countries (HICs), large data gaps remain. Only a few studies have been done to quantify antibiotic consumption globally,^14^, ^15^, ^16^, ^17^ all were based solely on a single data source, namely the IQVIA MIDAS dataset (including 76 mostly high-income and middle-income countries or territories), and did not provide estimates for individual countries not covered by the dataset.

This study aims to estimate global antibiotic consumption using two different types of data: first, antibiotic consumption data, referring to the aggregated amount of antibiotics consumed by a country, derived from national sales and import and reimbursement databases, obtained from multiple sources; and second, antibiotic usage data, referring to the use of antibiotics by individual patients,^18^ derived from household surveys in LMICs. We combine these data and use geospatial modelling techniques to produce contemporary, cross-validated estimates of antibiotic consumption for 204 countries over 19 years, from 2000 to 2018.

## Methods

### Overview

We modelled antibiotic consumption in HICs and LMICs separately due to differing covariate effects and data availability (appendix p 4). For LMICs, we built two distinct models: first, we modelled antibiotic usage (individual patient-level data); and second, we used the estimates of this model to inform a model of antibiotic consumption (aggregated national-level data). For HICs a single model of antibiotic consumption was implemented. For the purposes of this study, China, Russia, and Lebanon were classified as HICs (appendix p 31); all other countries were categorised based on the World Bank classifications.^19^ This study complied with the Guidelines for Accurate and Transparent Health Estimates Reporting (appendix p 51).^20^ This research was exempted from ethics approval as all analyses on antibiotic usage (patient-level information) were done on publicly accessible de-identified data.

### Antibiotic usage data

Antibiotic usage data were extracted from household surveys, primarily the United States Agency for International Development's Demographic Health Surveys (DHS) and UNICEF's Multiple Indicator Cluster Surveys (MICS; appendix pp 7–18). These large nationally representative surveys are regularly done in LMICs, use standardised data collections forms and methods, providing internationally comparable data for multiple years. Caregivers of children (aged <5 years) were asked whether the child had suffered from symptoms of lower respiratory tract infection (assessed as cough, fever, difficulty or rapid breathing, and chest symptoms) in the previous 2 weeks and whether the child received antibiotics for this illness (only yes or no answers were possible). We have referred to the information from surveys as antibiotic usage data.

Data on the proportion of children (aged <5 years) with symptoms of lower respiratory tract infection in the previous 2 weeks who received antibiotics, for the time period from 2000 to 2018, were extracted and linked to the smallest geographical location available: global positioning system cluster coordinates (point locations) or administrative districts (hereafter administrative level 1 areas will be referred to as states and administrative level 2 areas as districts). Administrative districts were resampled to point locations using population weighted K-means clustering^21^ (appendix p 19). Point and resampled administrative level data were combined and used as input data for the model.

### Antibiotic usage model

We used a two-stage Bayesian hierarchical model, established for fine scale mapping of health outcomes^2^, ^21^, ^22^, ^23^, ^24^ to estimate the proportion of antibiotic usage for each 5 × 5 km grid cell across our study area (LMICs), and each year from 2000 to 2018. In the first stage, a stacked ensemble model (using generalised additive models, boosted regression trees, and elastic-net penalised regression) was fit to the data using sociodemographic, health, and environmental covariates (appendix p 21). Five-fold cross-validation was used to obtain out-of-sample predictions and estimates from these models used as the explanatory covariates to fit a spatially and temporally explicit binomial Bayesian generalised linear model, with a logit link function, predicting the probability of antibiotic usage. The residual spatial and temporal error was modelled as a space-time Gaussian process with spatial covariance modelled as a stationary Matérn function and temporal covariance corresponding to an autoregressive 1 function by year. The model was fit using Integrated Nested Laplace Approximation in R statistical software (version 3.5.0); 1000 draws of the model were taken and the mean prediction calculated with 95% uncertainty intervals (UIs). Results were aggregated to national, state, and district resolutions at the draw level (based on the Database of Global Administrative Areas boundaries),^25^ preserving uncertainty in estimates.

Antibiotic usage was not estimated for North Korea or island nations with populations under 100 000 people due to a lack of sufficient survey data, covariates, and geographical contiguity. For these countries, national estimates were inferred as the median antibiotic usage for that region. Finally, we analysed the within-country variation in antibiotic usage by calculating the relative deviation of antibiotic usage in each district from the country mean (subtracting the national estimate from the district estimate and dividing by the national estimate), and summarising at the national level by calculating the range of these relative deviations.

### Antibiotic consumption data

We obtained antibiotic consumption data from IQVIA for 76 countries and territories from 2000 to 2018. Data were provided in two datasets; 2000–13 (IQVIA MIDAS) data were provided as standard units for each product of J01 class antibiotics (standard units are defined as a single dose of that particular compound [pills, capsules, or ampoules]), and data for 2014–18 (IQVIA Analytics) were provided in kilograms, aggregated for each J01 class antibiotic (regardless of product or preparation); we have referred to the combined data as the IQVIA dataset. Data were provided disaggregated by hospital and retail sectors.

We restricted the dataset to antibiotics for systemic use (J01 Anatomical Therapeutic Chemical [ATC] classification). Standard units (for 2000–13) were converted to kilograms and defined daily doses (DDD) calculated for each antibiotic following the latest versions of the WHO Collaborating Centre for Drug Statistics Methodology and ATC/DDD index (the 2019 index included higher DDD values for nine antimicrobials and routes; appendix pp 25–27).^26^, ^27^ Where differing DDD values were provided for oral and parenteral preparations, expert opinion was used to determine the most appropriate value (appendix p 27).

24 (32%) countries contained only data from the retail sector; for these data points the values for antibiotic consumption in hospitals were imputed using classification and regression trees (CART) multiple imputation method from the MICE package (version 3.7.0) in R (version 3.6.1). The data for all J01 antibiotics consumed in hospital and retail sectors were aggregated to provide one value for total consumption per country year.

Additional sources of national antibiotic consumption data from which DDD were calculable for countries and years not included in the IQVIA datasets were obtained from the WHO report on surveillance of antibiotic consumption (referred to as the WHO dataset), which contains consumption data from 65 countries for 2015–16;^18^ European Surveillance of Antimicrobial Consumption Network (ESAC-Net) from the European Centre for Disease Control and Prevention which report data from 27 European countries for 1998–2018;^12^ and published literature from Samoa for 2004^28^ and Kenya for 1997–2001.^29^ The total DDD per 1000 population for all J01 antibiotics were calculated by country and year using population estimates from the Global Burden of Disease (GBD) study. The final dataset covered 47 (68%) of 69 HICs and 65 (48%) of 135 LMICs.

### Modelling of antibiotic consumption

#### HICs

Data coverage for HICs was high (99·2% population coverage); countries with missing data (appendix p 31) were generally small. We deemed a spatial and temporal model of antibiotic consumption unsuitable for HICs, due to the lack of spatial trends in antibiotic consumption and inadequate availability of covariates to capture complex associations. The total DDD per 1000 population for the missing HICs and years were estimated using the CART multiple imputation method.

#### LMICs

For LMICs, data coverage was sparser than for HICs (78·3% population coverage). To account for this sparsity of data and to leverage the estimates of antibiotic usage in LMICs, we used a two-stage modelling framework to estimate antibiotic consumption (in DDD per 1000 population) for each LMIC from 2000 to 2018. The estimates of antibiotic usage prevalence, aggregated to the national level, were used as a covariate (in addition to antenatal care coverage, hospital beds per 1000 persons, temperature, outdoor air pollution, sanitation, physicians per capita, Health Access and Quality Index, and socio-demographic index) to fit a stacked ensemble model. Stacking has been shown to improve predictive validity compared with non-ensemble modelling methodologies, to aid with covariate selection and to account for non-linearity and interactions between covariates.^30^ These estimates were then smoothed in space and time using a Spatial-Temporal Gaussian Process Regression model.^31^ All models were validated using five-fold cross validation. Full details of this modelling strategy and model validation are available in the appendix (pp 32–39).

The estimates of antibiotic consumption in LMICs and HICs were combined to present global antibiotic consumption in DDD per 1000 population per day for each year, from 2000 to 2018. Uncertainty for the LMIC antibiotic consumption model was estimated by taking 1000 draws of the Gaussian process regression and calculating the 95% UIs. Uncertainty was not calculated for HICs due to the differing modelling strategies.

#### Consumption by antibiotic classes

A spatial-temporal modelling approach to estimate consumption by antibiotic class was deemed inappropriate, due to the lack of spatial trends and association to health and sociodemographic covariates for the data. Antibiotic consumption for each class of antibiotics (ATC level 3 and select ATC level 4 classes) and WHO AWaRe categories^32^ were calculated by applying the proportions of antibiotic consumption in each group from the cleaned antibiotic consumption data (before imputing missing values) to the total mean antibiotic consumption estimated for each country and year. In countries with no input data, the proportions for the GBD region to which the country belonged were used.

### Role of the funding source

The funders of the study had no role in study design, data collection, data analysis, data interpretation, or writing of the report.

## Results

From 2000 to 2018, data from 209 surveys and 284 045 children (aged <5 years), covering 101 countries and 19 years, were input into the model of antibiotic usage in LMICs (135 countries; appendix pp 5–6). All the results are available on the Nuffield Department of Medicine's website. Results showed high spatial variability in our estimates of antibiotic usage, both between and within countries.

**Figure 1:**
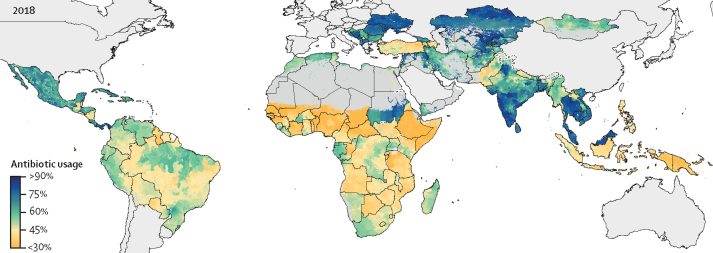
The percentage of children (aged <5 years) with symptoms of lower respiratory tract infections with caregiver-reported antibiotic usage in low-income and middle-income countries, 2018 Modelled estimates are shown by level two administrative divisions. High-income countries and pixels (1×1 km) with populations of less than ten people are shown in grey.

In 2018, antibiotic usage was highest across the central Europe, eastern Europe, and central Asia super-region, where the median national antibiotic usage was 72% (IQR 60–78), with national estimates highest in Ukraine (80%, 95% UI 68–89), Kazakhstan (80%, 74–85), Bosnia and Herzegovina (79%, 73–85), and Belarus (79%, 73–84; figure 1; appendix pp 41–42). The lowest levels of antibiotic usage were estimated for sub-Saharan Africa, with a median national usage of 42% (IQR 35–49) and Mauritania having the lowest national usage of any country (23%, 95% UI 15–32). Conversely, antibiotic use was considerably higher in North Africa and Middle East (median 61%, IQR 56–68), with notable variation between neighbouring countries—Sudan had high usage (73%, 95% UI 69–77) and Ethiopia low usage (28%, 95% UI 24–31).

In southeast Asia antibiotic usage was very diverse with a median usage of 51% (IQR 46–63), with high usage estimated in Vietnam (74%, 95% UI 68–80) and Malaysia (79%, 71–85), whereas Papua New Guinea (33%, 16–54), Philippines (42%, 28–56), and Indonesia (43%, 33–54) were notably lower.

**Figure 2:**
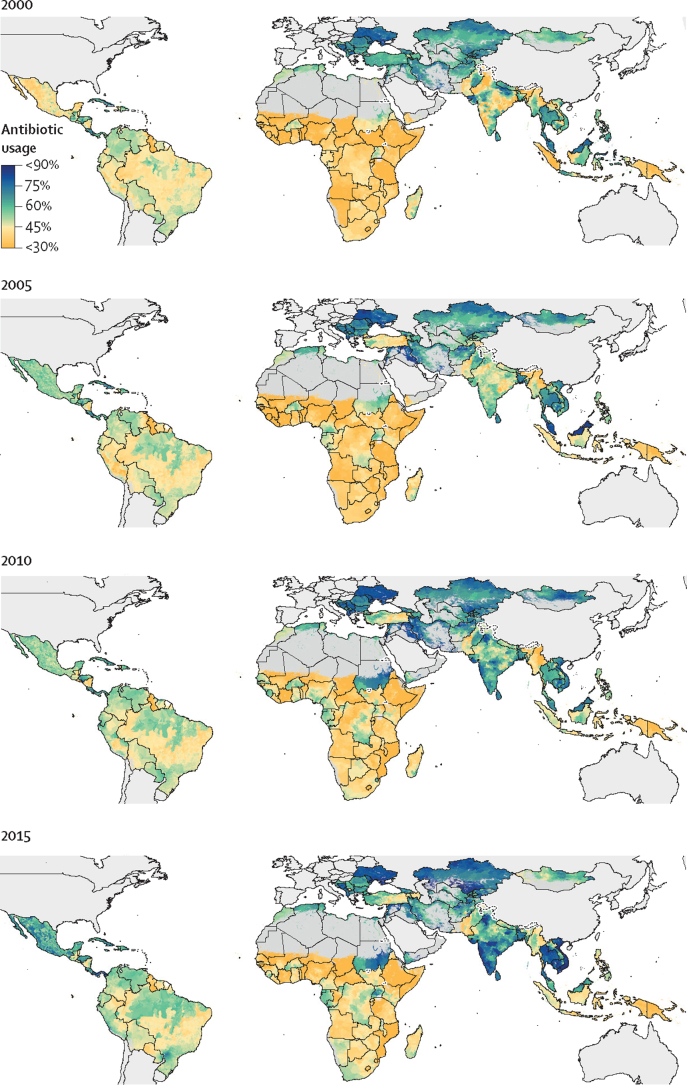
5 yearly estimates of the percentage of children (aged <5 years) with lower respiratory tract infections with caregiver-reported antibiotic usage in low-income and middle-income countries Modelled estimates are shown by level two administrative divisions. High-income countries and pixels (1×1 km) with populations of less than ten people are shown in grey.

From 2000 to 2018, the prevalence of antibiotic usage was consistently low in sub-Saharan Africa and consistently high in eastern Europe and central Asia (figure 2). However, dramatic increases in antibiotic usage were noted in Sudan (from 49% [95% UI 45–55] to 73% [69–77]), India (from 48% [41–56] to 67% [58–74]), and parts of southeast Asia (Vietnam, 59% [50–67] to 74% [68–80]; and Cambodia, 63% [51–73] to 77% [70–84]).

**Figure 3:**
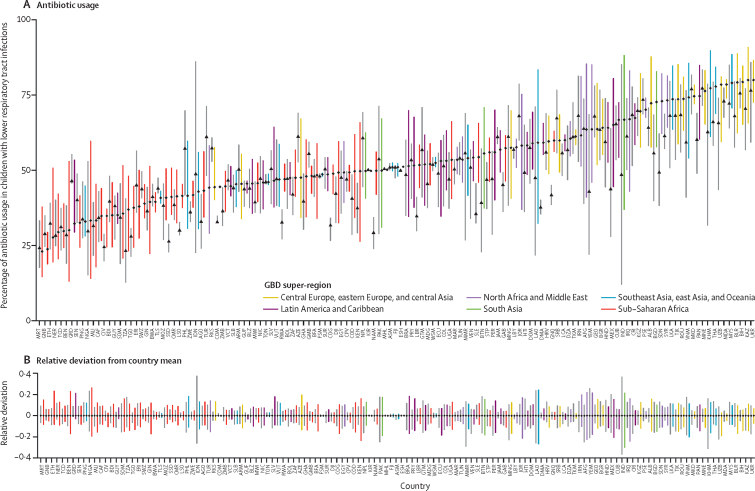
Within-country variation in antibiotic usage in 2000 and 2018 (A) Bars show the range in antibiotic usage for districts within each country; coloured bars represent estimates from 2018 and grey bars represent estimates from 2000. Points represents the mean percentage of antibiotic usage for each country, with diamonds representing 2018 and triangles representing 2000. (B) Bars show the range in the relative deviation from the mean for antibiotic usage in each country; coloured bars represent estimates from 2018 and the grey bars represent estimates from 2000. The 2018 colours are based on the GBD super-regions to which the country belongs, and countries are ordered (on the x-axis) based on the mean antibiotic usage in 2018 (ascending). Countries are labelled using the International Organisation for Standardisation codes. GBD=Global Burden of Disease.

We identified high inequalities in antibiotic usage within countries; in 2018, the relative deviation in antibiotic usage was highest in India and Laos (both 51% relative deviation). In sub-Saharan Africa, the relative deviation in antibiotic usage was highest in Nigeria (46%) and Benin (40%). Four countries in sub-Saharan Africa (Benin, Ethiopia, Nigeria, and Mauritania) were home to the 50 districts with the lowest proportion of antibiotic usage. Overall, there was little difference in the relative deviation in antibiotic usage between 2000 and 2018; however, decreases were noted in India (73% to 51%), Indonesia (63% to 25%), and Cambodia (47% to 28%; figure 3).

**Figure 4:**
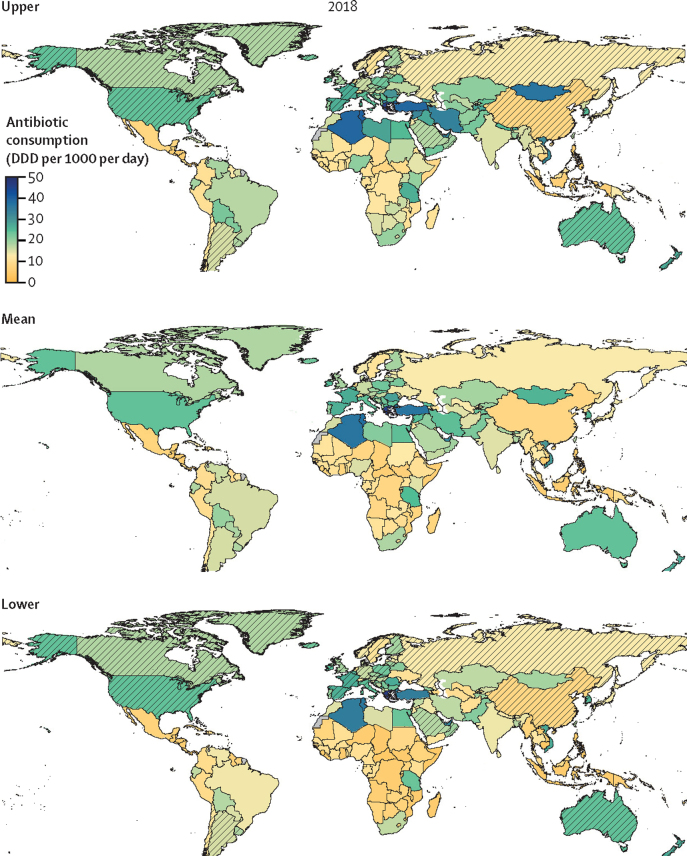
Total antibiotic consumption rates for 2018, with uncertainty intervals Estimates of antibiotic consumption rates in DDD per 1000 population per day. The mean map combines the modelled estimates for LMICs, and the imputed dataset for HICs. The upper and lower maps represent the upper and lower 95% uncertainty intervals for the LMIC model, with HICs shaded. The time-series of total antibiotic consumption rates in DDD per 1000 population per day from 2000 to 20015 is shown in the appendix (p 45). DDD=defined daily doses. HIC=high-income country. LMIC=low-income and middle-income country.

**Figure 5:**
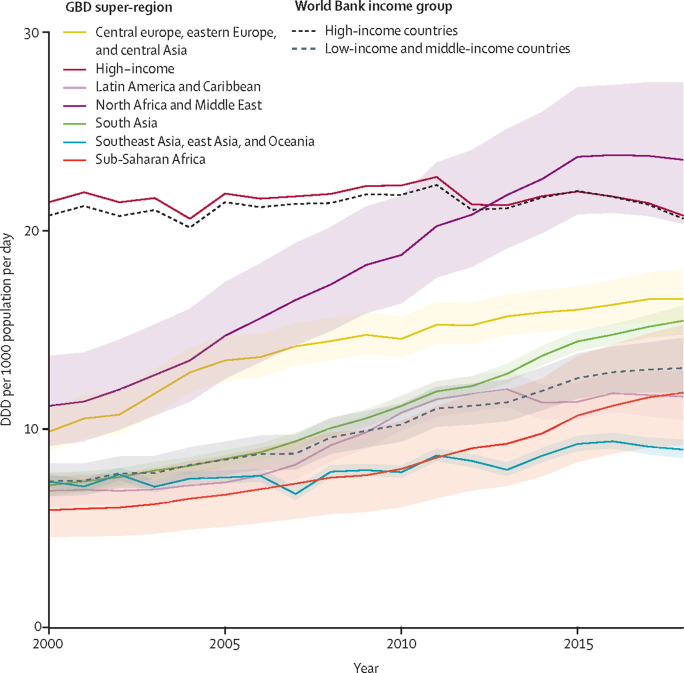
Temporal trends in the total antibiotic consumption rates for GBD super-regions and World Bank income groups Modelled estimates of antibiotic consumption rates in DDD per 1000 population per day from 2000 to 2018. Estimates are plotted as the total for each GBD super-region (solid lines) and each World Bank income group (dashed lines), with the 95% uncertainty intervals represented by the coloured ribbons (not available for high-income countries). DDD=defined daily doses. GBD=Global Burden of Disease.

We estimate that global antibiotic consumption was 40·1 (95% UI 37·2–43·7) billion DDD in 2018, equating to a rate of 14·1 (13·2–15·6) DDD per 1000 population per day (UIs represent the uncertainty from the LMICs model; therefore results from HICs are not displayed with accompanying UIs). Antibiotic consumption was inequitable between World Bank classified HICs and LMICs, with respective rates of 20·6 DDD per 1000 per day and 13·1 (95% UI 11·8–14·6) DDD per 1000 per day (Figure 4, Figure 5). South Asia (India, Pakistan, Nepal, Bangladesh, and Bhutan), with a population of 1·8 billion, consumed over a quarter (25·2%) of all antibiotics in 2018, whereas China alone (1·4 billion population) consumed 10%. Antibiotic consumption rates were highest in North Africa and Middle East (23·6 [95% UI 20·4–27·5] DDD per 1000 per day), closely followed by high-income North America (23·4 DDD per 1000 per day). The lowest rate in Central sub-Saharan Africa (8·2 [5·9–11·3] DDD per 1000 per day; table).

**Table:** Antibiotic consumption estimates by GBD super-region and GBD region, for the year 2018

		**Volume of total antibiotic consumption (Million DDD, 95%UI)**	**Global antibiotic consumption**	**Rate of total antibiotic consumption (DDD per 1000 per day, 95% UI)**
Southeast Asia, east Asia, and Oceania				
	Super-region estimate	7048 (6717–7437)*	17·5%	9·0 (8·5–9·5)*
	East Asia	4413 (4383–4454)*	11·0%	8·2 (8·2–8·3)*
	Southeast Asia	2592 (2305–2926)	6·5%	10·6 (9·4–12·0)
	Oceania	42 (29–57)	0·1%	9·3 (6·5–12·6)
Central Europe, eastern Europe, and central Asia				
	Super-region estimate	2525 (2333–2752)*	6·3%	16·5 (15·3–18·0)
	Central Asia	546 (433–682)	1·4%	16·2 (12·8–20·2)
	Central Europe	951 (922–984)	2·4%	22·5 (21·8–23·3)
	Eastern Europe	1028 (977–1086)*	2·6%	13·4 (12·8–14·2)*
High income				
	Super-region estimate	8196*	20·4%	20·8*
	High-income Asia-Pacific	1142*	2·8%	16·7*
	Australasia	257*	0·6%	24·5*
	Western Europe	3364*	8·4%	21·1*
	Southern Latin America	342*	0·9%	14·2*
	High-income North America	3092*	7·7%	23·4*
Latin America and Caribbean				
	Super-region estimate	2477 (2226–2758)	6·2%	11·6 (10·5–13·0)
	Caribbean	166 (135–207)	0·4%	10·2 (8·3–12·7)
	Andean Latin America	319 (296–343)	0·8%	13·6 (12·6–14·6)
	Central Latin America	792 (733–856)	2·0%	8·5 (7·8–9·1)
	Tropical Latin America	1200 (1062–1352)	3·0%	15·1 (13·4–17)
North Africa and Middle East				
	Super-region estimate	5229 (4515–6095)	13·0%	23·6 (20·4–27·5)
South Asia				
	Super-region estimate	10 104 (9650–10 598)	25·2%	15·5 (14·8–16·2)
Sub-Saharan Africa				
	Super-region estimate	4586 (3564–5905)	11·4%	11·8 (9·2–15·2)
	Central sub-Saharan Africa	387 (276–533)	1·0%	8·2 (5·9–11·3)
	Eastern sub-Saharan Africa	1788 (1374–2324)	4·5%	12·2 (9·4–15·9)
	Southern sub-Saharan Africa	445 (391–509)	1·1%	15·6 (13·7–17·9)
	Western sub-Saharan Africa	1966 (1524–2538)	4·9%	11·9 (9·2–15·4)
Global		40 165 (37 200–43 740)*	100·0%	14·3 (13·2–15·6)*

Data are DDD (95% UI) or %. DDD=defined daily doses. GBD=Global Burden of Disease. UI=uncertainty interval.

* UIs were not estimated for high-income countries, Russia, Lebanon, and China.

We identified large spatial disparities in the rates of antibiotic consumption, with the rates in Greece (45·9 DDD per 1000 per day) and Tunisia (38·0 [95% UI 36·0–40·2] DDD per 1000 per day) almost ten times higher than the lowest rates found in countries such as the Philippines (5·0 [4·8–5·3]), Honduras (5·6 [5·4–5·9]), Burundi (5·3 [4·3–6·4]), and Central African Republic (5·7 [4·0–8·0]; figure 4). Rates of antibiotic consumption differed notably between neighbouring countries, with Mongolia (26·1 [18·3–36·9]) surpassing consumption rates in China (8·0) and Sudan having noticeably higher consumption (12·3 [8·4–16·8]) than neighbouring South Sudan (6·5 [4·5–9·1]) and Chad (6·9 [4·8–9·8]). Similarly, low consumption was estimated in Indonesia (5·3 [4·5–6·2]) and Philippines (5·0 [4·8–5·3]) in stark contrast to high consumption in Vietnam (30·0 [27·7–32·5]), Thailand (12·4 [11·8–13·2]), and Malaysia (9·9 [9·4–10·5]).

Global antibiotic consumption rates increased from 9·8 (95% UI 9·2–10·5) DDD per 1000 per day in 2000 to 14·3 (13·2–15·6) in 2018. This differed greatly between HICs and LMICs; although consumption rates remained stable in HICs across the study period (20·8 DDD per 1000 per day in 2000 compared to 20·6 in 2018), there was a 76% increase observed in LMICs (from 7·4 [95% UI 6·6–8·3] DDD per 1000 per day in 2000 to 13·1 [11·8–14·6] in 2018; figure 5). The largest increases in antibiotic consumption were seen in the North Africa and Middle East region (111% increase from 11·2 [9·2–13·7] DDD per 1000 per day in 2000 to 23·6 [20·4–27·5] in 2018) and south Asia (116% increase from 7·2 [6·6–7·8] DDD per 1000 per day in 2000 to 15·5 [14·8–16·2] in 2018); for all other regions (excluding the high-income super-region that decreased slightly) more modest increases in consumption were seen (22–100% increases; figure 5). The time-series of global antibiotic consumption rates from 2000 to 2015 is shown in the appendix (p 45).

We identified large variations in the consumption rates of antibiotic classes (ATC level 3 and 4 groups; appendix pp 46–47). Examining key antibiotics in selected GBD super-regions, we identified stable rates of broad-spectrum penicillin consumption over the study period, with the highest rates in 2018 in the high-income super-region (3·9 DDD per 1000 per day), and lowest in south Asia (0·8 DDD per 1000 per day; appendix p 47). Carbapenem consumption was highest in the high-income super-region, where it increased from 0·05 to 0·09 DDD per 1000 per day from 2000 to 2018. Fluoroquinolone consumption increased in all super-regions and in 2018 was highest in North Africa and Middle East (2·7 DDD per 1000 per day) and south Asia (3·0 DDD per 1000 per day). The consumption of third-generation cephalosporins increased more than ten-fold in North Africa and Middle East (from 0·09 DDD per 1000 per day in 2000 to 1·7 in 2018) and south Asia (from 0·09 DDD per 1000 per day in 2000 to 3·4 in 2018), but remained lower in the high-income super-region (1·1 DDD per 1000 per day) and sub-Saharan Africa (0·5 DDD per 1000 per day) super-regions in 2018 (appendix p 47).

## Discussion

We provide the first estimates of longitudinal human antibiotic consumption for 204 countries from 2000 to 2018, using a novel approach that applied spatial modelling techniques and incorporated multiple data sources, including large-scale antibiotic usage surveys. Our results highlight important spatial disparities in antibiotic consumption and provide estimates for countries lacking surveillance data. We identified high rates of antibiotic consumption in high-income and upper-middle-income countries in North America, Europe, and the Middle East, contrasted by very low rates of consumption in sub-Saharan Africa and parts of southeast Asia. Total antibiotic consumption rates showed a nearly ten-fold variation, from as low as 5·0 DDD per 1000 population per day in the Philippines to 45·9 DDD per 1000 per day in Greece. Additionally, to our knowledge, our analysis is the first global longitudinal analysis of consumption that applies the higher 2019 DDD values^26^, ^27^ for nine antibiotics and routes (including amoxicillin and ampicillin, the most consumed broad-spectrum antibiotics).

Studies analysing antibiotic consumption on a global scale and evaluating time trends are scarce,^14^, ^15^, ^16^ reflecting the lack of surveillance data in LMICs. A previous analysis (using the ATC/DDD methodology) of global antibiotic consumption from 2000 to 2015^14^ relied exclusively on IQVIA MIDAS data and derived global estimates by extrapolating from countries within the same income group. Our estimates of global antibiotic consumption were comparable, albeit slightly lower with 38·3 (95% UI 36·1–41·2) billion DDD (14·1 [95% UI 13·3–15·2] DDD per 1000 per day) for 2015 compared with the 42·3 billion DDD (15·7 DDD per 1000 per day) by Klein and colleagues.^14^ The differences were most apparent in HICs, where our estimates of 20·6 DDD per 1000 per day in 2018 were considerably lower than those of 25·7 DDD per 1000 per day in 2015 by Klein and colleagues. This discrepancy is partially explained by our use of the updated DDD values^26^, ^27^ for key antibiotics such as ampicillin and amoxicillin; analysing the IQVIA dataset using the historic DDD values would result in consumption rates of 24·4 DDD per 1000 per day in HICs for 2018. Notably, this difference was not observed in LMICs (appendix p 50), which might reflect a higher proportional use of other antibiotic classes in LMICs, especially those classified into the WHO Watch category.^16^, ^32^

Both inappropriate use of antibiotics and lack of access to antibiotics are important public health problems^33^ and are highlighted by our study. Some of the highest rates of antibiotic consumption are estimated in HICs, with countries such as the USA identifying up to 30% of antibiotic prescriptions as unnecessary.^34^ We estimate the lowest rate of antibiotic consumption to be in sub-Saharan Africa, a region characterised by the highest prevalence of sepsis.^35^ Although the lack of access and delayed access to antibiotics is an important contributing factor to the high mortality in children younger than 5 years in LMICs,^3^, ^36^ high levels of inapropriate antibiotic use have been reported in many LMICs,^37^, ^38^ especially in south and southeast Asia, where a recent study identified high levels of both non-licensed antibiotic vendors and self-medication.^39^ Lack of access to high quality health care and inappropriate use of antibiotics often coexist within one health system and need to be tackled in tandem to ensure appropriate treatment of bacterial infections and to preserve the efficacy of antibiotics.^33^, ^38^

Previous publications have shown gross domestic product to be significantly associated with antibiotic consumption in LMICs,^14^ and we identified a suite of sociodemographic and health-related covariates that drove the model of antibiotic consumption. However, in HICs these covariates had no association with the observed patterns in consumption. Drivers of antibiotic use in HICs were found to be related to cultural and behavioural attitudes of the populations,^40^, ^41^, ^42^ as well as attitudes of prescribers, and awareness of antibiotic efficacy and resistance.^41^, ^43^ This resulted in few identifiable spatial patterns in antibiotic consumption in HICs, precluding the use of spatial–temporal modelling to estimate total antibiotic consumption. Additionally, standardised covariates for numerous factors hypothesised to impact antibiotic usage and consumption were not available to be used in our model (eg, antibiotic prescription policy and implementation). This limited the impact of covariates within the model and future iterations of this work should focus on improving the availability of such covariates. Our modelling strategy was focused on prediction, and the use of the same covariates for various stages of the model precludes our ability to infer causation at any stage.

Our study enhances our understanding of antibiotic use in the community by providing detailed subnational estimates for caregiver-reported antibiotic usage in children (aged <5 years) with lower respiratory tract infections in LMICs from 2000 to 2018. We identified large variations in the proportion of children with reported antibiotic use across and within LMICs. In 2018, the highest levels of antibiotic usage were estimated in the central Europe, eastern Europe, and central Asia super-region (median antibiotic usage of 72%), and the lowest levels for sub-Saharan Africa (median antibiotic usage 43%). Our subnational estimates highlight inequalities in antibiotic usage within countries, underlining issues of access to health care, particularly in poor and rural communities. A recent study^44^ also used household surveys to estimate antibiotic usage in children presenting with fever, diarrhoea, and lower respiratory tract infections across LMICs, and grouped the results by WHO and World Bank regions. The authors estimated the lowest usage to be in southeast Asia.^44^ Possible explanations for these differences might be regional estimates of antibiotic usage disguising spatial and temporal trends, and differing probabilities of antibiotic usage for distinct infectious disease syndromes; however, it was not possible to determine the exact reasons for these differences from the provided data sources at this time.

We identified not only large variations in the rates of total antibiotic consumption across countries, but also in the proportion of antibiotics classes used in different geographical contexts.^16^ Our results show the highest rates of broad-spectrum penicillin consumption in the high-income super-region and the lowest in south Asia. In south Asia, notable increases in fluoroquinolone (1·8-fold) and third-generation cephalosporin (37-fold) consumption rates occurred during the study period, driving AMR. Our findings are corroborated by a recent study^16^ that estimated that WHO Access antibiotics made up less than 30% of total antibiotic consumption in India in 2015. The heterogeneity in antibiotic classes consumed in our global study likely reflect differing treatment guidelines, or non-adherence, and self-medication in less regulated markets.^45^ It is crucial that antibiotic stewardship interventions are strengthened to achieve the WHO target of 60% of Access antibiotic consumption.^16^, ^32^

The main limitations of our modelling study are centred around data quality and availability issues. Data on antibiotic usage in LMICs are sparse, with most studies focusing on the knowledge, attributes, and practices of antibiotic usage in specific study locations.^46^, ^47^, ^48^ Although such studies are methodologically strong and provide a detailed insight into location-specific antibiotic usage, there is often limited comparability between studies. Although the DHS and MICS are the only sources of standardised, representative, and internationally comparable data, they contain multiple biases: first, small sample sizes restrict the ability to accurately determine prevalence of disease and antibiotic usage from the sample;^49^ second, the use of caregiver-reported symptoms is inaccurate in determining disease occurrence (the denominator); and third, the inaccuracy of caregiver identification of antibiotics introduces further uncertainty.^49^ Furthermore, it is not possible to identify antibiotic classes (due to the dichotomous nature of the question) or ascertain the correct indication for antibiotic use. There are currently no alternatives to these surveys in LMICs; if interpreted with care, they provide a useful resource for comparing spatial and temporal trends in antibiotic usage.^49^, ^50^ The expansion of antibiotic usage questions within the DHS and MICS series would be beneficial, and could involve the extension to all household members. Additionally, distinct appearance^47^ and labelling,^51^ such as used by the Red Line Campaign, might improve correct identification of antibiotics.

Another limitation stems from the fact that we used antibiotic usage in children (aged <5 years) to model antibiotic consumption in the general population in LMICs, assuming that the spatial–temporal trends in antibiotic usage between adults and children were comparable. High antibiotic usage in adults is corroborated by health-care utilisation surveys done in LMICs that show a strong trend for adults to access community pharmacies as first point of contact and to self-medicate with over-the-counter antibiotics;^39^, ^52^, ^53^, ^54^ however, the prevalence of antibiotic usage in children might over-estimate that in adults due to the higher frequencies of paediatric respiratory infections and health-care visits.^37^ Limited data on the sales and use of suboptimal (substandard or falsified) antibiotics exists and, therefore, has not been incorporated into our analysis.^55^, ^56^

The IQVIA MIDAS database is a proprietary database, restricting access and analysis. For the 24 included countries lacking data from hospitals, we relied on multiple imputation methods to estimate total antibiotic consumption. Additionally, for some countries, such as India, the IQVIA MIDAS hospital data exclude public facilities, which could underestimate consumption. Only formal channels of antibiotic sales are included in IQVIA MIDAS; therefore sales of antibiotics from informal markets, which could make up a considerable proportion of antibiotic sales in LMICs,^57^, ^58^ might not be included in these data.

The lack of antibiotic consumption data in LMICs is another limitation. Our combined dataset only covers 48% of LMICs, highlighting the urgent need for the establishment of antibiotic consumption surveillance programmes,^18^ especially in LMICs, to improve estimates of consumption and aid in the implementation of antibiotic stewardship programmes, including enforcement of existing regulations.^8^ The sparse data in LMICs could also affect the robustness of our estimates of proportional consumption of antibiotic classes (we used the proportion of the relevant GBD region for countries lacking data); the proportional consumption of pharmacological subgroups in individual countries might differ from the regional estimate. Moreover, our study pertains to the definition of DDD as an adult antibiotic maintenance dose. As antibiotic doses for children are substantially lower, this could lead to an underestimation of antibiotic exposures in countries with a young demographic.^26^ Lastly, antibiotic sales and consumption data do not necessarily equate to antibiotic exposures, as this is dependent on patients' compliance; however, they represent the best data available.

To our knowledge, our study provides the first longitudinal antibiotic consumption estimates for 204 countries from 2000 to 2018, presenting an important benchmark of antibiotic consumption and highlighting spatial disparities in antibiotic usage and consumption. We identified countries with very high antibiotic consumption, indicating excess use, and countries with very low consumption, indicative of a lack of access to essential antibiotics. These findings underline the scope and potential major benefits of antibiotic stewardship interventions, aided by antibiotic consumption surveillance,^8^ to patient outcomes, reduction of harm, and safe-guarding the future effectiveness of antibiotics. It is imperative to curb the unnecessary demand for antibiotics and combat AMR by improving drinking water and sanitation, vaccine coverage, and the availability of rapid diagnostic testing,^4^ while also increasing access to antibiotics where they are needed the most.

## Data sharing

The source code for all analysis is available at https://github.com/NDM-GRAM/Antibiotic-consumption. Interactive data visualisations and all estimates, including antibiotic usage estimates aggregated to national, state, and district levels, and antibiotic consumption estimates for total J01 antibiotic consumption, consumption by ATC level 3 classes, and consumption by select ATC level 4 classes are available at www.ndm.ac/gram-project/antibiotic-consumption.

## Declaration of interests

AS has been the recipient of research grants from the Bill & Melinda Gates Foundation. All other authors declare no competing interests.

## References

[1] Dicker D, Nguyen G, Abate D (2018). Global, regional, and national age-sex-specific mortality and life expectancy, 1950-2017: a systematic analysis for the Global Burden of Disease Study 2017. Lancet.

[2] Burstein R, Henry NJ, Collison ML (2019). Mapping 123 million neonatal, infant and child deaths between 2000 and 2017. Nature.

[3] Laxminarayan R, Matsoso P, Pant S (2016). Access to effective antimicrobials: a worldwide challenge. Lancet.

[4] O'Neill J (2016). Tackling drug-resistant infections globally: final report and recommendations. https://amr-review.org/sites/default/files/160525_Final%20paper_with%20cover.pdf.

[5] Goossens H, Ferech M, Vander Stichele R, Elseviers M (2005). Outpatient antibiotic use in Europe and association with resistance: a cross-national database study. Lancet.

[6] Albrich WC, Monnet DL, Harbarth S (2004). Antibiotic selection pressure and resistance in *Streptococcus pneumoniae* and *Streptococcus pyogenes*. Emerg Infect Dis.

[7] van de Sande-Bruinsma N, Grundmann H, Verloo D (2008). Antimicrobial drug use and resistance in Europe. Emerg Infect Dis.

[8] WHO (2015). Global action plan on antimicrobial resistance. https://apps.who.int/iris/bitstream/handle/10665/193736/9789241509763_eng.pdf?sequence=1.

[9] UN General Assembly (2015). Transforming our world: the 2030 agenda for sustainable development. https://sdgs.un.org/sites/default/files/publications/21252030%20Agenda%20for%20Sustainable%20Development%20web.pdf.

[10] Pisani E (2015). Antimicrobial resistance: what does medicine quality have to do with it?. https://amr-review.org/sites/default/files/ElizabethPisaniMedicinesQualitypaper.pdf.

[11] Simonsen GS, Tapsall JW, Allegranzi B, Talbot EA, Lazzari S (2004). The antimicrobial resistance containment and surveillance approach—a public health tool. Bull World Health Organ.

[12] European Centre for Disease Prevention and Control (2018).

[13] Versporten A, Bolokhovets G, Ghazaryan L (2014). Antibiotic use in eastern Europe: a cross-national database study in coordination with the WHO Regional Office for Europe. Lancet Infect Dis.

[14] Klein EY, Van Boeckel TP, Martinez EM (2018). Global increase and geographic convergence in antibiotic consumption between 2000 and 2015. Proc Natl Acad Sci USA.

[15] Van Boeckel TP, Gandra S, Ashok A (2014). Global antibiotic consumption 2000 to 2010: an analysis of national pharmaceutical sales data. Lancet Infect Dis.

[16] Klein EY, Milkowska-Shibata M, Tseng KK (2020). Assessment of WHO antibiotic consumption and access targets in 76 countries, 2000–15: an analysis of pharmaceutical sales data. Lancet Infect Dis.

[17] Col NF, O'Connor RW (1987). Estimating worldwide current antibiotic usage: report of Task Force 1. Rev Infect Dis.

[18] WHO (2018). WHO report on surveillance of antibiotic consumption: 2016–2018 early implementation. https://apps.who.int/iris/bitstream/handle/10665/277359/9789241514880-eng.pdf?ua=1.

[19] The World Bank World Bank country and lending groups. https://datahelpdesk.worldbank.org/knowledgebase/articles/906519.

[20] Stevens GA, Alkema L, Black RE (2016). Guidelines for Accurate and Transparent Health Estimates Reporting: the GATHER statement. Lancet.

[21] Golding N, Burstein R, Longbottom J (2017). Mapping under-5 and neonatal mortality in Africa, 2000–15: a baseline analysis for the Sustainable Development Goals. Lancet.

[22] Osgood-Zimmerman A, Millear AI, Stubbs RW (2018). Mapping child growth failure in Africa between 2000 and 2015. Nature.

[23] Reiner RC, Wiens KE, Deshpande A (2020). Mapping geographical inequalities in childhood diarrhoeal morbidity and mortality in low-income and middle-income countries, 2000–17: analysis for the Global Burden of Disease Study 2017. Lancet.

[24] Wiens K, Lindstedt PA, Blacker BF, Johnson KB, Baumann MM, Schaeffer LE (2020). Mapping geographical inequalities in oral rehydration therapy coverage in low-income and middle-income countries, 2000-17. Lancet Glob Health.

[25] Global Administrative Areas (2018). Old GADM data. https://gadm.org/old_versions.html.

[26] WHO Collaborating Centre for Drug Statistics Methodology. Guidelines for ATC classification and DDD assignment 2020. Norway: 2019.

[27] WHO Collaborating Centre for Drug Statistics Methodology (2019). ATC/DDD Index 2020. https://www.whocc.no/atc_ddd_index/.

[28] Norris P, Nguyen HA (2007). Consumption of antibiotics in a small Pacific island nation: Samoa. Pharm Pract (Granada).

[29] Mitema ES, Kikuvi GM (2004). Surveillance of the overall use of antimicrobial drugs in humans over a 5 year period (1997–2001) in Kenya. J Antimicrob Chemother.

[30] Bhatt S, Cameron E, Flaxman SR, Weiss DJ, Smith DL, Gething PW (2017). Improved prediction accuracy for disease risk mapping using Gaussian process stacked generalization. J R Soc Interface.

[31] Stanaway JD, Afshin A, Gakidou E (2018). Global, regional, and national comparative risk assessment of 84 behavioural, environmental and occupational, and metabolic risks or clusters of risks for 195 countries and territories, 1990-2017: a systematic analysis for the Global Burden of Disease Study 2017. Lancet.

[32] WHO (2019). World Health Organization model list of essential medicines, 21^st^ List. https://www.who.int/publications/i/item/WHOMVPEMPIAU2019.06.

[33] Mendelson M, Røttingen JA, Gopinathan U (2016). Maximising access to achieve appropriate human antimicrobial use in low-income and middle-income countries. Lancet.

[34] Fleming-Dutra KE, Hersh AL, Shapiro DJ (2016). Prevalence of inappropriate antibiotic prescriptions among US ambulatory care visits, 2010-2011. JAMA.

[35] Rudd KE, Johnson SC, Agesa KM (2020). Global, regional, and national sepsis incidence and mortality, 1990-2017: analysis for the Global Burden of Disease Study. Lancet.

[36] Liu L, Oza S, Hogan D (2015). Global, regional, and national causes of child mortality in 2000-13, with projections to inform post-2015 priorities: an updated systematic analysis. Lancet.

[37] Fink G, D'Acremont V, Leslie HH, Cohen J (2020). Antibiotic exposure among children younger than 5 years in low-income and middle-income countries: a cross-sectional study of nationally representative facility-based and household-based surveys. Lancet Infect Dis.

[38] Sulis G, Gandra S (2021). Access to antibiotics: not a problem in some LMICs. Lancet Glob Health.

[39] Do NTT, Vu HTL, Nguyen CTK (2021). Community-based antibiotic access and use in six low-income and middle-income countries: a mixed-method approach. Lancet Glob Health.

[40] Kenyon C, Manoharan-Basil SS (2020). Cultural drivers of antibiotic consumption in high-income countries: A global ecological analysis. Microb Drug Resist.

[41] Borg MA (2012). National cultural dimensions as drivers of inappropriate ambulatory care consumption of antibiotics in Europe and their relevance to awareness campaigns. J Antimicrob Chemother.

[42] Gaygısız Ü, Lajunen T, Gaygısız E (2017). Socio-economic factors, cultural values, national personality and antibiotics use: a cross-cultural study among European countries. J Infect Public Health.

[43] Harbarth S, Albrich W, Brun-Buisson C (2002). Outpatient antibiotic use and prevalence of antibiotic-resistant pneumococci in France and Germany: a sociocultural perspective. Emerg Infect Dis.

[44] Allwell-Brown G, Hussain-Alkhateeb L, Kitutu FE, Strömdahl S, Mårtensson A, Johansson EW (2020). Trends in reported antibiotic use among children under 5 years of age with fever, diarrhoea, or cough with fast or difficult breathing across low-income and middle-income countries in 2005-17: a systematic analysis of 132 national surveys from 73 countries. Lancet Glob Health.

[45] Auta A, Hadi MA, Oga E (2019). Global access to antibiotics without prescription in community pharmacies: a systematic review and meta-analysis. J Infect.

[46] Elong Ekambi G-A, Okalla Ebongue C, Penda IC, Nnanga Nga E, Mpondo Mpondo E, Eboumbou Moukoko CE (2019). Knowledge, practices and attitudes on antibiotics use in Cameroon: self-medication and prescription survey among children, adolescents and adults in private pharmacies. PLoS One.

[47] Cambaco O, Alonso Menendez Y, Kinsman J (2020). Community knowledge and practices regarding antibiotic use in rural Mozambique: where is the starting point for prevention of antibiotic resistance?. BMC Public Health.

[48] Abubakar U, Tangiisuran B (2020). Knowledge and practices of community pharmacists towards non-prescription dispensing of antibiotics in Northern Nigeria. Int J Clin Pharm.

[49] Campbell H, El Arifeen S, Hazir T (2013). Measuring coverage in MNCH: challenges in monitoring the proportion of young children with pneumonia who receive antibiotic treatment. PLoS Med.

[50] Padget M, Guillemot D, Delarocque-Astagneau E (2016). Measuring antibiotic consumption in low-income countries: a systematic review and integrative approach. Int J Antimicrob Agents.

[51] Kakkar M, Walia K, Vong S, Chatterjee P, Sharma A (2017). Antibiotic resistance and its containment in India. BMJ.

[52] Panzner U, Pak GD, Aaby P (2016). Utilization of healthcare in the typhoid fever surveillance in Africa program. Clin Infect Dis.

[53] Morgan DJ, Okeke IN, Laxminarayan R, Perencevich EN, Weisenberg S (2011). Non-prescription antimicrobial use worldwide: a systematic review. Lancet Infect Dis.

[54] Alhomoud F, Aljamea Z, Almahasnah R, Alkhalifah K, Basalelah L, Alhomoud FK (2017). Self-medication and self-prescription with antibiotics in the Middle East-do they really happen? A systematic review of the prevalence, possible reasons, and outcomes. Int J Infect Dis.

[55] Almuzaini T, Choonara I, Sammons H (2013). Substandard and counterfeit medicines: a systematic review of the literature. BMJ Open.

[56] Kelesidis T, Falagas ME (2015). Substandard/counterfeit antimicrobial drugs. Clin Microbiol Rev.

[57] Khare S, Purohit M, Sharma M (2019). Antibiotic prescribing by informal healthcare providers for common illnesses: a repeated cross-sectional study in rural India. Antibiotics (Basel).

[58] Suy S, Rego S, Bory S (2019). Invisible medicine sellers and their use of antibiotics: a qualitative study in Cambodia. BMJ Glob Health.

