# Supplementary material for: Global antibiotic consumption and usage in humans, 2000–18: a spatial modelling study
Source: Lancet Planet Health. 2021 Nov 12;5(12):e893–904. doi: 10.1016/S2542-5196(21)00280-1 (PMC8654683; doi:10.1016/S2542-5196(21)00280-1)
Supplement: Supplementary appendix [file mmc1.pdf]

THE LANCET
Planetary Health

Supplementary appendix

This appendix formed part of the original submission and has been peer reviewed. We post it as supplied by the authors.

Supplement to: Browne AJ, Chipeta MG, Haines-Woodhouse G, et al. Global antibiotic consumption and usage in humans, 2000–18: a spatial modelling study. *Lancet Planet Health* 2021; published online Nov 11. http://dx.doi.org/10.1016/S2542-5196(21)00280-1.

Contents

List of Figures and Tables **2**
Figures 2
Tables 2
Supplementary Methods **4**
i. Overview 4
1. Antibiotic use data 5
1.1 Data extraction 5
1.2 Polygon resampling 19
1.3 Data preparation 19
2. Antibiotic usage model 20
2.1 Covariate selection 20
2.2 Stacked ensemble model 21
2.3 Geostatistical model 22
2.4 Model validation 23
3. Antibiotic consumption data preparation 23
3.1 IQVIA data processing 23
3.2 IQVIA missing data imputation 24
3.3 WHO data and correlation between IQVIA Analytics and WHO estimates 28
3.4 ESAC-NET data 29
3.5 Combining data sources 30
4. Antibiotic consumption model 31
4.1 High income countries antibiotic consumption model specifications 31
4.2 Low- and middle- income countries antibiotic consumption model specifications 32
4.3 Types of antibiotics consumed 39
Supplementary Results **40**
1. Antibiotic usage model results 40
1.1 Model results 40
1.2 Model validation 43
2. Global antibiotic consumption model results 44
2.1 Model results 44
2.3 Model validation 48
2.4 Impact of updated daily defined dose values 49
Gather Checklist **51**
References **52**

List of Figures and Tables

Figures

Figure S1: Schematic overview of the study

Figure S2: Map of data availability for the antibiotic use model.

Figure S3: Plot of data availability by country and year.

Figure S4: Polygon resampling.

Figure S5: Regions for the model of antibiotic use

Figure S6. Covariates included in the model of antibiotic usage for each modelling regions

Figure S7: Finite elements mesh for the South Asia region.

Figure S8: Comparison of IQVIA and WHO antibiotic consumption data.

Figure S9: Comparison of IQVIA and the ESAC-NET antibiotic consumption dataset.

Figure S10. All data sources included in the model of antibiotic consumption.

Figure S11: Relative uncertainty of antibiotic usage for 2018.

Figure S12. Estimates of the proportion of antibiotic use in LMICs for 2018 with the mean and upper and lower 95% uncertainty intervals.

Figure S13. Estimates of the proportion of antibiotic use in LMICs for 2018 at varying spatial aggregations.

Figure S14. Validation plots for the model of antibiotic use.

Figure S15. Validation plots for the model of antibiotic use for each modelling region

Figure S16. Five yearly estimates of total antibiotic consumption rates from 2000 to 2015

Figure S17: Antibiotic consumption by ATC level 3 class. Data are expressed in DDDs/1,000 population/day

Figure S18: Temporal trends in antibiotic consumption by class (ATC level 3) for 2000-2018

Figure S19: Temporal trends in consumption of selected antibiotic classes (ATC level 4), displayed for four selected GBD super regions

Figure S20: Proportion of total antibiotics consumed by AWaRe categories.

Figure S21. Validation plots for the model of antibiotic consumption.

Tables

Table S1: Surveys included in the antibiotic use model

Table S2: LRI cross-walk coefficients

Table S3: Covariates included in the model of antibiotic use

Table S4: DDD values for each included J01 antibiotic

Table S5: AWaRe categorisation of J01 antibiotics

Table S6. Global Burden of Disease location hierarchy

Table S7. In- and out-of-sample metrics for the five-fold cross validation models on antibiotic use

Table S8: Total DDDs and DDD/1000/day in HICs and LMICs for 2000-2018 based on 2019 DDD values and pre-2019 DDD values

Table S9: GATHER checklist

Supplementary Methods

i. Overview

Figure S1 shows a schematic overview of the data processing and modelling for this study

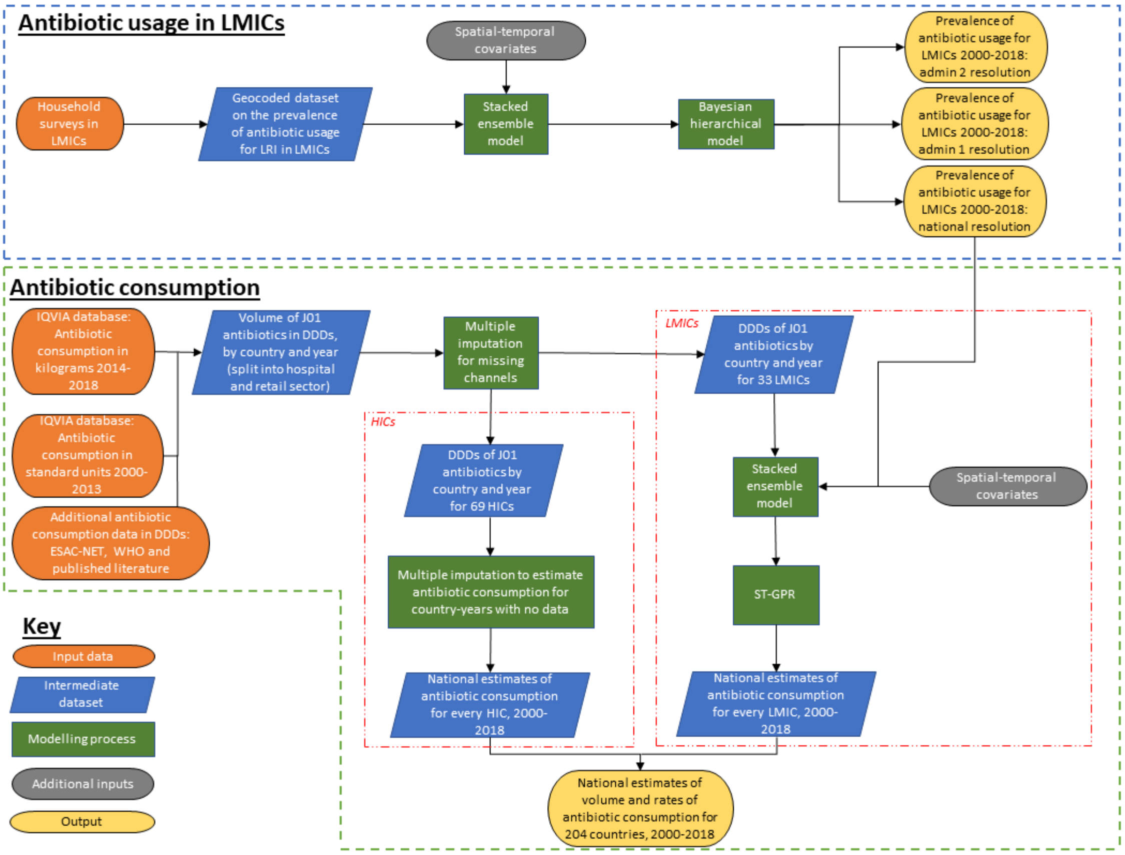

Figure S1: Schematic overview of the study

1. Antibiotic use data

1.1 Data extraction

Data were extracted from 209 surveys covering 284,045 children < 5 years with LRI. Details of the included surveys are supplied in Table S1. We extracted data for all children under five years whose caregivers reported them having displayed symptoms of cough in the previous two weeks. Additional binary indicators extracted for those children were caregiver reported a) difficulty or rapid breathing, b) chest symptoms, c) fever, and d) use of antibiotics. Surveys had to report symptoms of cough and antibiotic use (binary indicator, "yes" or "no"), and be linkable to subnational locations (either latitudes and longitudes or administrative divisions) to be included in the extracted dataset.

Extracted data covered 101 countries (Figure S2) and 19 years (Figure S3). Data were linked to the smallest location available, either point locations or administrative divisions. Point locations were used directly in the geostatistical model whilst administrative divisions were linked to polygons in shapefiles which were then resampled to point locations (see section 1.2).

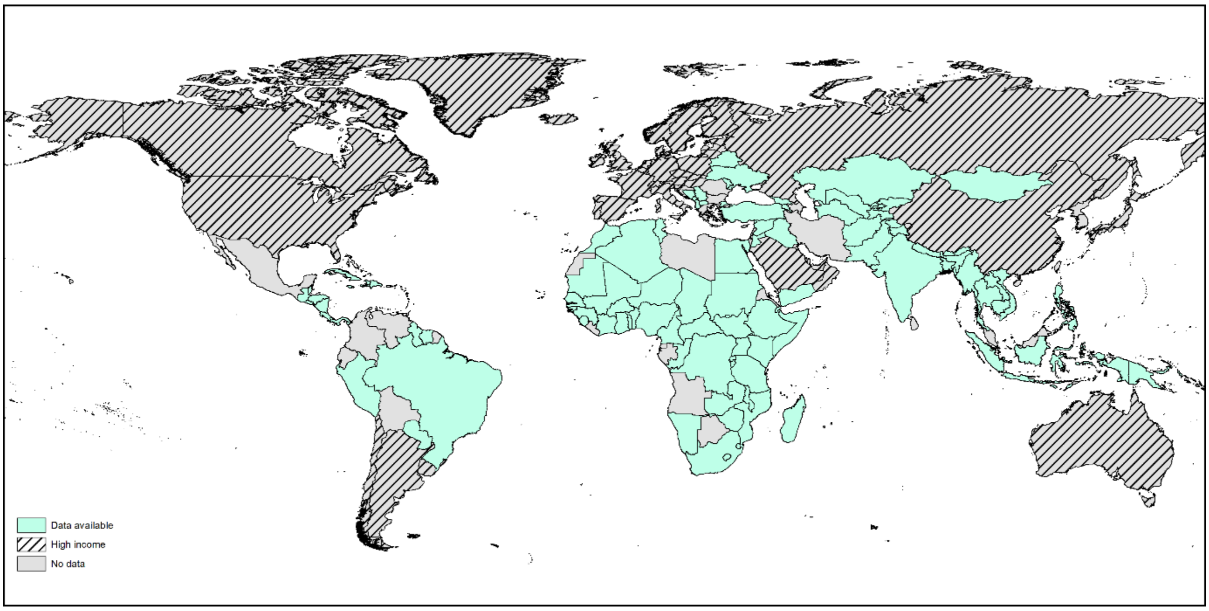

Figure S2: Map of data availability for the antibiotic usage model. Low and middle-income countries with household surveys contributing to the model are in turquoise, countries with no data are in grey, whilst high-income countries (which are not being modelled) are shown in grey with diagonal stripes.

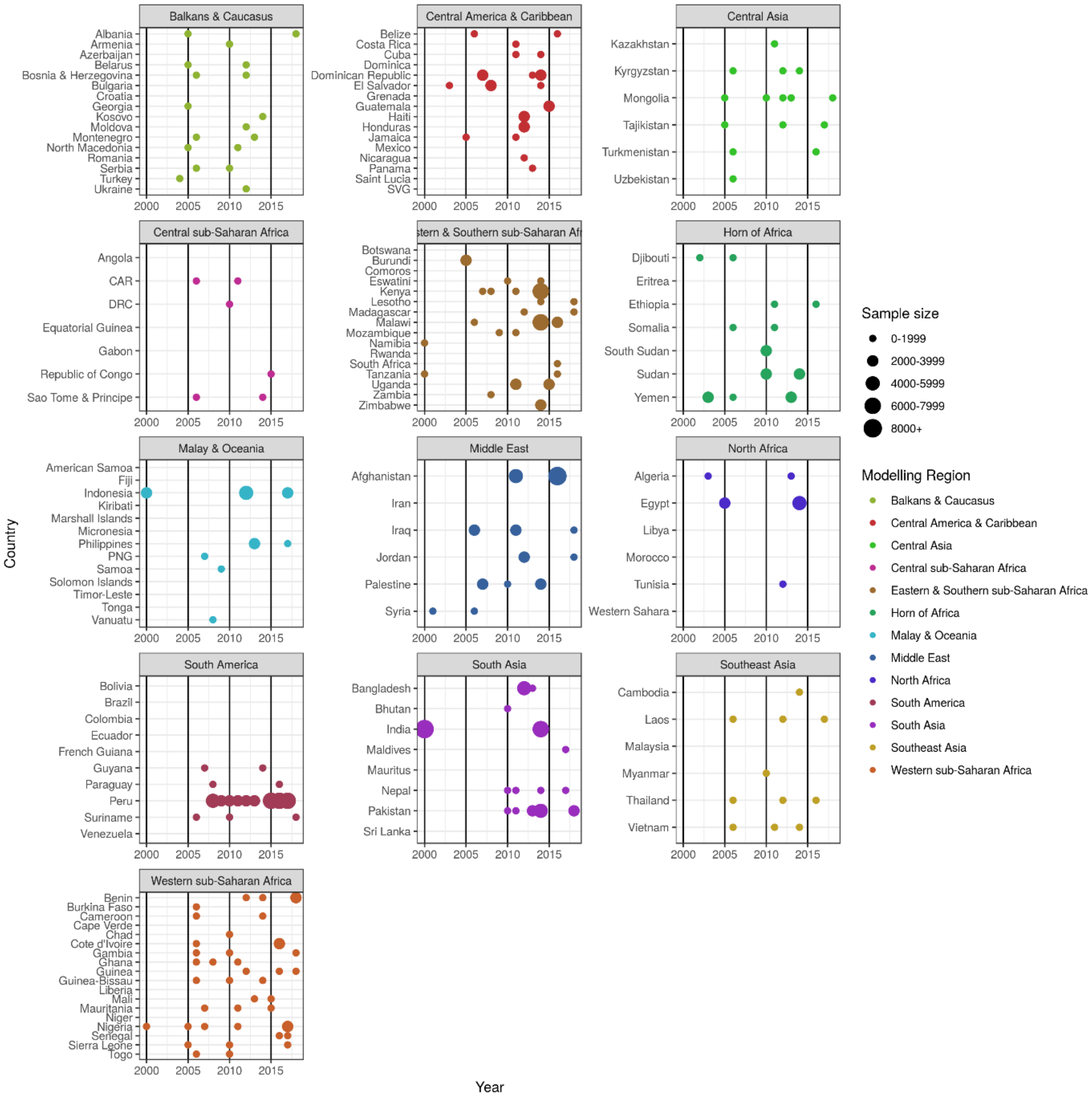

Figure S3: Plot of data availability for antibiotic usage by country and year. The x-axis indicates the year and the y-axis the country in which the survey was undertaken**.** The size of the points is representative of the number of children with LRI in each survey, and the colour indicates the modelling region to which that country belongs.

Country	Year	Survey series	Citation
Afghanistan	2011	Multiple Indicator Cluster Survey (MICS)	Central Statistics Organization (Afghanistan), United Nations Children's Fund (UNICEF). Afghanistan Multiple Indicator Cluster Survey 2010-2011. New York, United States of America: United Nations Children's Fund (UNICEF), 2013.
Afghanistan	2016	Demographic and Health Survey (DHS)	Central Statistics Organization (Afghanistan), ICF International, Ministry of Public Health (Afghanistan). Afghanistan Demographic and Health Survey 2015-2016. Fairfax, United States of America: ICF International, 2017.
Albania	2005	Multiple Indicator Cluster Survey (MICS)	National Institute of Statistics (Albania), United Nations Children's Fund (UNICEF). Albania Multiple Indicator Cluster Survey 2005. New York, United States: United Nations Children's Fund (UNICEF).
Albania	2018	Demographic and Health Survey (DHS)	Albania Institute of Public Health (IPH), Albania Institute of Statistics (INSTAT), ICF International. Albania Demographic and Health Survey 2017-2018. Fairfax, United States: ICF International, 2019.
Algeria	2003	Pan Arab Project for Family Health (PAPFAM)	National Office of Statistics (Algeria), Ministry of Health, Population and Hospital Reform (Algeria), League of Arab States. Algeria Family Health Survey 2002-2003.
Algeria	2013	Multiple Indicator Cluster Survey (MICS)	Ministry of Health and Population (Algeria), United Nations Children's Fund (UNICEF). Algeria Multiple Indicator Cluster Survey 2012-2013. New York, United States of America: United Nations Children's Fund (UNICEF), 2018.
Argentina	2012	Multiple Indicator Cluster Survey (MICS)	National Institute of Statistics and Censuses (Argentina), National Secretariat for Children, Youth, and Family (SENAF), Ministry of Social Development (Argentina), National University of La Matanza (Argentina), National University of Tres de Febrero (Argentina), United Nations Children's Fund (UNICEF). Argentina Multiple Indicator Cluster Survey 2011-2012. New York, United States: United Nations Children's Fund (UNICEF), 2015.
Armenia	2010	Demographic and Health Survey (DHS)	ICF Macro, Ministry of Health (Armenia), National Statistical Service of the Republic of Armenia. Armenia Demographic and Health Survey 2010. Fairfax, United States of America: ICF International, 2015.
Bangladesh	2012	Demographic and Health Survey (DHS)	ICF Macro, Mitra and Associates, National Institute of Population Research and Training (NIPORT). Bangladesh Demographic and Health Survey 2011-2012. Calverton, United States of America: ICF Macro.
Bangladesh	2013	Multiple Indicator Cluster Survey (MICS)	Bangladesh Bureau of Statistics (BBS), Government of Bangladesh, Ministry of Planning (Bangladesh), United Nations Children's Fund (UNICEF). Bangladesh Multiple Indicator Cluster Survey 2012-2013. New York, United States of America: United Nations Children's Fund (UNICEF), 2015.
Barbados	2012	Multiple Indicator Cluster Survey (MICS)	Barbados Statistical Service, United Nations Children's Fund (UNICEF). Barbados Multiple Indicator Cluster Survey 2012. New York, United States: United Nations Children's Fund (UNICEF).
Belarus	2005	Multiple Indicator Cluster Survey (MICS)	Ministry of Statistics and Analysis of the Republic of Belarus, United Nations Children's Fund (UNICEF). Belarus Multiple Indicator Cluster Survey 2005. New York, United States: United Nations Children's Fund (UNICEF).
Belarus	2012	Multiple Indicator Cluster Survey (MICS)	National Statistical Committee of the Republic of Belarus, United Nations Children's Fund (UNICEF). Belarus Multiple Indicator Cluster Survey 2012. New York, United States: United Nations Children's Fund (UNICEF), 2013.
Belize	2006	Multiple Indicator Cluster Survey (MICS)	Statistical Institute of Belize, United Nations Children's Fund (UNICEF). Belize Multiple Indicator Cluster Survey 2006. New York, United States of America: United Nations Children's Fund (UNICEF).
Belize	2016	Multiple Indicator Cluster Survey (MICS)	Government of Belize, Statistical Institute of Belize, UN Resident Coordinator Fund (UN ResCor), United Nations Children's Fund (UNICEF), United Nations Development Programme (UNDP). Belize Multiple Indicator Cluster Survey 2015-2016. New York, United States of America: United Nations Children's Fund (UNICEF), 2018.
Benin	2012	Demographic and Health Survey (DHS)	ICF International, National Institute of Statistics and Economic Analysis (INSAE) (Benin), National Program Against AIDS (PNLS) (Benin). Benin Demographic and Health Survey 2011-2012. Fairfax, United States of America: ICF International, 2014.
Benin	2014	Multiple Indicator Cluster Survey (MICS)	National Institute of Statistics and Economic Analysis (INSAE) (Benin), United Nations Children's Fund (UNICEF). Benin Multiple Indicator Cluster Survey 2014. New York, United States of America: United Nations Children's Fund (UNICEF), 2017.
Benin	2018	Demographic and Health Survey (DHS)	Hubert Koutoukou Maga National University Hospital Center (CNHU-HKM)(Benin), ICF International, National Institute of Statistics and Economic Analysis (INSAE) (Benin), National Malaria Control Program, Ministry of Health (Benin), Permanent Secretariat of the Food Council and Nutrition (SP-CAN)(Benin). Benin Demographic and Health Survey 2017-2018. Fairfax, United States of America: ICF International, 2018.

Bhutan	2010	Multiple Indicator Cluster Survey (MICS)	National Statistics Bureau (Bhutan), United Nations Children's Fund (UNICEF), United Nations Population Fund (UNFPA). Bhutan Multiple Indicator Cluster Survey 2010. New York, United States: United Nations Children's Fund (UNICEF).
Bosnia and Herzegovina	2006	Multiple Indicator Cluster Survey (MICS)	Directorate for Economic Planning (Bosnia and Herzegovina), Federal Office of Statistics (Federation of Bosnia and Herzegovina), Institute of Statistics (Republic of Srpska), Ministry of Health (Federation of Bosnia and Herzegovina), Ministry of Health and Social Welfare (Republic of Srpska), Public Health Institute of Federation of Bosnia and Herzegovina, United Nations Children's Fund (UNICEF). Bosnia and Herzegovina Multiple Indicator Cluster Survey 2006. New York, United States: United Nations Children's Fund (UNICEF).
Bosnia and Herzegovina	2012	Multiple Indicator Cluster Survey (MICS)	Agency for Statistics (Bosnia and Herzegovina), Federal Ministry of Health (Bosnia and Herzegovina), Ministry of Health and Social Welfare (Republic of Srpska), Public Health Institute of Federation of Bosnia and Herzegovina, United Nations Children's Fund (UNICEF), United Nations Entity for Gender Equality and the Empowerment of Women (UN Women). Bosnia and Herzegovina Multiple Indicator Cluster Survey 2011-2012.
Burkina Faso	2006	Multiple Indicator Cluster Survey (MICS)	National Institute of Statistics and Demography (Burkina Faso), United Nations Children's Fund (UNICEF). Burkina Faso Multiple Indicator Cluster Survey 2006. New York, United States of America: United Nations Children's Fund (UNICEF).
Burundi	2005	Multiple Indicator Cluster Survey (MICS)	United Nations Children's Fund (UNICEF), Burundi Institute of Statistics and Economic Studies, United Nations Population Fund (UNFPA). Burundi Multiple Indicator Cluster Survey 2005. New York, United States: United Nations Children's Fund (UNICEF).
Côte d'Ivoire	2006	Multiple Indicator Cluster Survey (MICS)	United Nations Children's Fund (UNICEF), National Institute of Statistics (Côte d'Ivoire). Côte d'Ivoire Multiple Indicator Cluster Survey 2006. New York, United States: United Nations Children's Fund (UNICEF).
Cambodia	2014	Demographic and Health Survey (DHS)	ICF International, Ministry of Health (Cambodia), National Institute of Statistics (Cambodia). Cambodia Demographic and Health Survey 2014. Fairfax, United States of America: ICF International, 2017.
Cameroon	2006	Multiple Indicator Cluster Survey (MICS)	United Nations Children's Fund (UNICEF), National Institute of Statistics (Cameroon). Cameroon Multiple Indicator Cluster Survey 2006. New York, United States: United Nations Children's Fund (UNICEF).
Cameroon	2014	Multiple Indicator Cluster Survey (MICS)	Ministry of Public Health (Cameroon), National Institute of Statistics (Cameroon), United Nations Children's Fund (UNICEF). Cameroon Multiple Indicator Cluster Survey 2014. New York, United States of America: United Nations Children's Fund (UNICEF), 2017.
Central African Republic	2006	Multiple Indicator Cluster Survey (MICS)	United Nations Children's Fund (UNICEF). Central African Republic Multiple Indicator Cluster Survey 2006. New York, United States: United Nations Children's Fund (UNICEF).
Central African Republic	2011	Multiple Indicator Cluster Survey (MICS), Demographic and Health Survey (DHS)	Central African Institute of Statistics, Economic and Social Studies (ICASEES) (Central African Republic), ICF International. Central African Republic Multiple Indicator Cluster Survey 2010-2011. Fairfax, United States of America: ICF International, 2013.
Chad	2010	Multiple Indicator Cluster Survey (MICS)	Ministry of Planning, Economy, and International Cooperation (Chad), National Institute for Statistics, Economic and Demographic Studies (INSEED) (Chad), United Nations Children's Fund (UNICEF). Chad Multiple Indicator Cluster Survey 2010. New York, United States of America: United Nations Children's Fund (UNICEF), 2014.
Congo	2015	Multiple Indicator Cluster Survey (MICS)	National Institute of Statistics (INS) (Congo, Rep.), United Nations Children's Fund (UNICEF). Congo Multiple Indicator Cluster Survey 2014-2015. New York, United States of America: United Nations Children's Fund (UNICEF), 2018.
Costa Rica	2011	Multiple Indicator Cluster Survey (MICS)	Costa Rican Demographic Association, Ministry of Health (Costa Rica), United Nations Children's Fund (UNICEF). Costa Rica Multiple Indicator Cluster Survey 2011. New York, United States of America: United Nations Children's Fund (UNICEF), 2013.
Côte d'Ivoire	2016	Multiple Indicator Cluster Survey (MICS), Demographic and Health Survey (DHS)	National Institute of Statistics (Côte d'Ivoire), United Nations Children's Fund (UNICEF). Côte d'Ivoire Multiple Indicator Cluster Survey 2016. New York, United States of America: United Nations Children's Fund (UNICEF), 2018.
Cuba	2011	Multiple Indicator Cluster Survey (MICS)	Ministry of Public Health (Cuba), United Nations Children's Fund (UNICEF). Cuba Multiple Indicator Cluster Survey 2010-2011. New York, United States of America: United Nations Children's Fund (UNICEF).

Cuba	2014	Multiple Indicator Cluster Survey (MICS)	Ministry of Public Health (Cuba), National Office of Statistics (Cuba), United Nations Children's Fund (UNICEF). Cuba Multiple Indicator Cluster Survey 2014. New York, United States of America: United Nations Children's Fund (UNICEF), 2005.
Democratic Republic of the Congo	2010	Multiple Indicator Cluster Survey (MICS)	National Statistical Institute (Congo, DR), Ministry of Planning (Congo, DR), United Nations Children's Fund (UNICEF). Congo, DR Multiple Indicator Cluster Survey 2010. New York, United States: United Nations Children's Fund (UNICEF).
Djibouti	2002	Pan Arab Project for Family Health (PAPFAM)	Department of Statistics and Demographic Studies (Djibouti), League of Arab States, Ministry of Health (Djibouti), Pan Arab Project for Family Health (PAPFAM). Djibouti Family Health Survey 2002.
Djibouti	2006	Multiple Indicator Cluster Survey (MICS)	Ministry of Economy, Finance, and Planning in charge of Privatization (Djibouti), Ministry of Health (Djibouti), United Nations Children's Fund (UNICEF). Djibouti Multiple Indicator Cluster Survey 2006. New York, United States of America: United Nations Children's Fund (UNICEF).
Dominican Republic	2007	Demographic and Health Survey (DHS)	Center for Social and Demographic Studies (Dominican Republic) (CESDEM), Macro International, Inc. Dominican Republic Demographic and Health Survey 2007. Fairfax, United States of America: ICF International.
Dominican Republic	2013	Demographic and Health Survey (DHS)	Center for Social and Demographic Studies (Dominican Republic) (CESDEM), ICF International, Ministry of Public Health and Social Assistance (Dominican Republic). Dominican Republic Demographic and Health Survey 2013. Fairfax, United States of America: ICF International, 2014.
Dominican Republic	2013	Demographic and Health Survey (DHS)	Center for Social and Demographic Studies (Dominican Republic) (CESDEM), ICF International, National Public Health Laboratory (Dominican Republic). Dominican Republic Special Demographic and Health Survey 2013. Fairfax, United States of America: ICF International, 2015.
Dominican Republic	2014	Multiple Indicator Cluster Survey (MICS)	National Statistics Office (Dominican Republic), United Nations Children's Fund (UNICEF). Dominican Republic Multiple Indicator Cluster Survey 2014. New York, United States of America: United Nations Children's Fund (UNICEF), 2016.
Egypt	2005	Demographic and Health Survey (DHS)	El-Zanaty and Associates, Macro International, Inc, Ministry of Health and Population (Egypt), Population Council (Egypt). Egypt Demographic and Health Survey 2005. Fairfax, United States of America: ICF International.
Egypt	2014	Multiple Indicator Cluster Survey (MICS)	El-Zanaty and Associates, Ministry of Health and Population (Egypt), United Nations Children's Fund (UNICEF). Egypt IPHN Rural Districts Multiple Indicator Cluster Survey 2013-2014. New York, United States of America: United Nations Children's Fund (UNICEF), 2016.
Egypt	2014	Demographic and Health Survey (DHS)	El-Zanaty and Associates, ICF International, Ministry of Health and Population (Egypt). Egypt Demographic and Health Survey 2014. Fairfax, United States of America: ICF International.
El Salvador	2003	Reproductive Health Survey (RHS)	Asociación Demográfica Salvadoreña (ADS), Division of Reproductive Health-Centers for Disease Control and Prevention (CDC). (2004) El Salvador Reproductive Health Survey 2002-2003. San Salvador, El Salvador: ADS.
El Salvador	2008	Reproductive Health Survey (RHS)	Asociación Demográfica Salvadoreña (ADS), Division of Reproductive Health-Centers for Disease Control and Prevention (CDC). (2009) El Salvador Reproductive Health Survey 2008. San Salvador, El Salvador: ADS.
El Salvador	2014	Multiple Indicator Cluster Survey (MICS)	General Administration of Statistics and Censuses (El Salvador), Ministry of Health (El Salvador), United Nations Children's Fund (UNICEF). El Salvador Multiple Indicator Cluster Survey 2014. New York, United States of America: United Nations Children's Fund (UNICEF), 2017.
Ethiopia	2011	Demographic and Health Survey (DHS)	Central Statistical Agency (Ethiopia), ICF Macro, Ministry of Health (Ethiopia). Ethiopia Demographic and Health Survey 2010-2011. Fairfax, United States of America: ICF International.
Ethiopia	2016	Demographic and Health Survey (DHS)	Central Statistical Agency (Ethiopia), ICF International. Ethiopia Demographic and Health Survey 2016. Fairfax, United States of America: ICF International, 2017.
Gambia	2006	Multiple Indicator Cluster Survey (MICS)	Gambia Bureau of Statistics (GBOS), United Nations Children's Fund (UNICEF). Gambia Multiple Indicator Cluster Survey 2005-2006. New York, United States of America: United Nations Children's Fund (UNICEF).
Gambia	2010	Multiple Indicator Cluster Survey (MICS)	Gambia Bureau of Statistics (GBOS), United Nations Children's Fund (UNICEF). Gambia Multiple Indicator Cluster Survey 2010. New York, United States of America: United Nations Children's Fund (UNICEF), 2018.
Gambia	2018	Multiple Indicator Cluster Survey (MICS)	Gambia Bureau of Statistics (GBOS), United Nations Children's Fund (UNICEF). Gambia Multiple Indicator Cluster Survey 2018. New York, United States of America: United Nations Children's Fund (UNICEF), 2019.

Georgia	2005	Multiple Indicator Cluster Survey (MICS)	National Center for Disease Control (Georgia), State Department of Statistics of Georgia, United Nations Children's Fund (UNICEF). Georgia Multiple Indicator Cluster Survey 2005. New York, United States: United Nations Children's Fund (UNICEF).
Ghana	2006	Multiple Indicator Cluster Survey (MICS), Demographic and Health Survey (DHS)	Ministry of Health (MOH) (Ghana), Ghana Statistical Service and United Nations Children's Fund (UNICEF). Ghana Multiple Indicator Cluster Survey 2006. New York, United States: United Nations Children's Fund (UNICEF).
Ghana	2008	Multiple Indicator Cluster Survey (MICS)	Ghana Statistical Service, Ministry of Health (Ghana), United Nations Children's Fund (UNICEF). Ghana District Multiple Indicator Cluster Survey 2007-2008.
Ghana	2011	Multiple Indicator Cluster Survey (MICS)	Institute of Statistical, Social and Economic Research, University of Ghana, United Nations Children's Fund (UNICEF). Ghana - Accra Multiple Indicator Cluster Survey 2010-2011. New York, United States of America: United Nations Children's Fund (UNICEF), 2014.
Ghana	2011	Multiple Indicator Cluster Survey (MICS), Demographic and Health Survey (DHS)	Centers for Disease Control and Prevention (CDC), Ghana Statistical Service, Government of Japan, ICF Macro, Ministry of Health (Ghana), Navrongo Health Research Centre, United Nations Children's Fund (UNICEF), United Nations Population Fund (UNFPA), United States Agency for International Development (USAID). Ghana Multiple Indicator Cluster Survey 2011. New York, United States of America: United Nations Children's Fund (UNICEF), 2013.
Guatemala	2015	Demographic and Health Survey (DHS)	ICF International, Institute of Nutrition of Central America and Panama, Ministry of Public Health and Social Assistance (Guatemala), National Statistics Institute (Guatemala), Secretary of Planning and Programming of the Presidency (Segeplán) (Guatemala). Guatemala Demographic and Health Survey 2014-2015. Fairfax, United States of America: ICF International, 2017.
Guinea	2012	Demographic and Health Survey (DHS)	ICF Macro, Ministry of Health and Public Hygiene (Guinea), National Institute of Statistics (Guinea). Guinea Demographic and Health Survey 2012. Fairfax, United States of America: ICF International.
Guinea	2016	Multiple Indicator Cluster Survey (MICS)	National Institute of Public Health (NPHI) (Guinea), National Institute of Statistics (Guinea), National Malaria Control Program (Guinea), United Nations Children's Fund (UNICEF). Guinea Multiple Indicator Cluster Survey 2016. New York, United States of America: United Nations Children's Fund (UNICEF), 2018.
Guinea	2018	Demographic and Health Survey (DHS)	Ministry of Health (Guinea), Ministry of Planning and Economic Development (Guinea), National Institute of Statistics (Guinea). Guinea Demographic and Health Survey 2018. Fairfax, United States of America: ICF International, 2019.
Guinea-Bissau	2006	Multiple Indicator Cluster Survey (MICS)	United Nations Children's Fund (UNICEF), Government of Guinea-Bissau. Guinea-Bissau Multiple Indicator Cluster Survey 2006. New York, United States: United Nations Children's Fund (UNICEF).
Guinea-Bissau	2010	Multiple Indicator Cluster Survey (MICS)	Centers for Disease Control and Prevention (CDC), National Statistics Institute (Guinea-Bissau), United Nations Children's Fund (UNICEF). Guinea-Bissau Multiple Indicator Cluster Survey 2010. New York, United States: United Nations Children's Fund (UNICEF), 2018.
Guinea-Bissau	2014	Multiple Indicator Cluster Survey (MICS)	National Statistics Institute (Guinea-Bissau), United Nations Children's Fund (UNICEF). Guinea-Bissau Multiple Indicator Cluster Survey 2014. New York, United States of America: United Nations Children's Fund (UNICEF), 2016.
Guyana	2007	Multiple Indicator Cluster Survey (MICS)	United Nations Children's Fund (UNICEF), Bureau of Statistics (Guyana). Guyana Multiple Indicator Cluster Survey 2006. New York, United States: United Nations Children's Fund (UNICEF).
Guyana	2014	Multiple Indicator Cluster Survey (MICS)	Bureau of Statistics (Guyana), Ministry of Health (Guyana), United Nations Children's Fund (UNICEF). Guyana Multiple Indicator Cluster Survey 2014. New York, United States of America: United Nations Children's Fund (UNICEF), 2016.
Haiti	2012	Demographic and Health Survey (DHS)	Centers for Disease Control and Prevention (CDC), Haitian Institute of Childhood (IHE), Haitian Institute of Statistics and Informatics, Macro International, Inc. Haiti Demographic and Health Survey 2012. Fairfax, United States of America: ICF International.
Honduras	2012	Demographic and Health Survey (DHS)	ICF Macro, National Institute of Statistics (Honduras). Honduras Demographic and Health Survey 2011-2012. Fairfax, United States of America: ICF International.
India	2000	Demographic and Health Survey (DHS)	International Institute for Population Sciences (India). India Demographic and Health Survey 1992-1993. Mumbai, India: International Institute for Population Sciences (India).

India	2014	India District Level Household Survey (DLHS)	International Institute for Population Sciences (India). India District Level Household Survey 2012-2014. New Delhi, India: Ministry of Health and Family Welfare (India).
Indonesia	2000	Demographic and Health Survey (DHS)	Central Bureau of Statistics (Indonesia), Macro International, Inc, Ministry of Health (Indonesia), National Family Planning Coordinating Board (Indonesia). Indonesia Demographic and Health Survey 1991. Fairfax, United States: ICF International.
Indonesia	2012	Demographic and Health Survey (DHS)	ICF International, Ministry of Health (Indonesia), National Population and Family Planning Board (Indonesia), Statistics Indonesia. Indonesia Demographic and Health Survey 2012. Fairfax, United States of America: ICF International.
Indonesia	2017	Demographic and Health Survey (DHS)	Ministry of Health (Indonesia), National Population and Family Planning Board (Indonesia), Statistics Indonesia. Indonesia Demographic and Health Survey 2017. Fairfax, United States of America: ICF International, 2019.
Iraq	2006	Multiple Indicator Cluster Survey (MICS)	United Nations Children's Fund (UNICEF), Central Organization for Statistics and Information Technology (Iraq), Kurdistan Regional Statistics Office. Iraq Multiple Indicator Cluster Survey 2006. New York, United States: United Nations Children's Fund (UNICEF).
Iraq	2011	Multiple Indicator Cluster Survey (MICS)	Central Organization for Statistics and Information Technology (Iraq), Kurdistan Regional Statistics Office, Ministry of Health (Iraq), United Nations Children's Fund (UNICEF). Iraq Multiple Indicator Cluster Survey 2011. New York, United States of America: United Nations Children's Fund (UNICEF), 2013.
Iraq	2018	Multiple Indicator Cluster Survey (MICS)	Central Statistical Organization (Iraq), United Nations Children's Fund (UNICEF). Iraq Multiple Indicator Cluster Survey 2018. New York, United States of America: United Nations Children's Fund (UNICEF), 2019.
Jamaica	2005	Multiple Indicator Cluster Survey (MICS)	Statistical Institute of Jamaica (STATIN) and United Nations Children's Fund (UNICEF). Jamaica Multiple Indicator Cluster Survey 2005. New York, United States: United Nations Children's Fund (UNICEF).
Jamaica	2011	Multiple Indicator Cluster Survey (MICS)	Statistical Institute of Jamaica, United Nations Children's Fund (UNICEF). Jamaica Multiple Indicator Cluster Survey 2011. New York, United States of America: United Nations Children's Fund (UNICEF), 2015.
Jordan	2012	Demographic and Health Survey (DHS)	Department of Statistics (Jordan), ICF International. Jordan Demographic and Health Survey 2012. Fairfax, United States of America: ICF International.
Jordan	2018	Demographic and Health Survey (DHS)	Department of Statistics (Jordan), ICF International. Jordan Demographic and Health Survey 2017-2018. Fairfax, United States of America: ICF International, 2019.
Kazakhstan	2011	Multiple Indicator Cluster Survey (MICS)	Agency of the Republic of Kazakhstan on Statistics, United Nations Children's Fund (UNICEF). Kazakhstan Multiple Indicator Cluster Survey 2010-2011. New York, United States: United Nations Children's Fund (UNICEF), 2013.
Kenya	2007	Multiple Indicator Cluster Survey (MICS)	Kenya National Bureau of Statistics, United Nations Children's Fund (UNICEF). Kenya - North Eastern Province Multiple Indicator Cluster Survey 2007. Nairobi, Kenya: Kenya National Bureau of Statistics.
Kenya	2008	Multiple Indicator Cluster Survey (MICS)	Kenya National Bureau of Statistics, United Nations Children's Fund (UNICEF). Kenya - Eastern Province Multiple Indicator Cluster Survey 2008. Nairobi, Kenya: Kenya National Bureau of Statistics.
Kenya	2011	Multiple Indicator Cluster Survey (MICS)	Kenya National Bureau of Statistics, United Nations Children's Fund (UNICEF). Kenya - Nyanza Province Multiple Indicator Cluster Survey 2011. Nairobi, Kenya: Kenya National Bureau of Statistics.
Kenya	2014	Multiple Indicator Cluster Survey (MICS)	Kenya National Bureau of Statistics, Population Studies and Research Institute, University of Nairobi (Kenya), United Nations Children's Fund (UNICEF). Kenya - Turkana County Multiple Indicator Survey 2013-2014. New York, United States of America: United Nations Children's Fund (UNICEF), 2015.
Kenya	2014	Multiple Indicator Cluster Survey (MICS)	Kenya National Bureau of Statistics, Population Studies and Research Institute, University of Nairobi (Kenya), United Nations Children's Fund (UNICEF). Kenya - Kakamega County Multiple Indicator Survey 2013-2014. New York, United States of America: United Nations Children's Fund (UNICEF), 2015.
Kenya	2014	Multiple Indicator Cluster Survey (MICS)	Kenya National Bureau of Statistics, Population Studies and Research Institute, University of Nairobi (Kenya), United Nations Children's Fund (UNICEF). Kenya - Bungoma County Multiple Indicator Survey 2013-2014. New York, United States of America: United Nations Children's Fund (UNICEF), 2015.
Kenya	2014	Demographic and Health Survey (DHS)	ICF International, Kenya Medical Research Institute (KEMRI), Kenya National Bureau of Statistics, Ministry of Health (Kenya), National AIDS Control Council (Kenya), National Council for Population and Development (Kenya). Kenya Demographic and Health Survey 2014. Fairfax, United States of America: ICF International.

Kosovo	2014	Multiple Indicator Cluster Survey (MICS)	Kosovo Agency of Statistics, United Nations Children's Fund (UNICEF). Kosovo Multiple Indicator Cluster Survey 2013-2014. New York, United States: United Nations Children's Fund (UNICEF), 2015.
Kyrgyzstan	2006	Multiple Indicator Cluster Survey (MICS)	United Nations Children's Fund (UNICEF), National Statistical Committee of the Kyrgyz Republic. Kyrgyzstan Multiple Indicator Cluster Survey 2005-2006. New York, United States: United Nations Children's Fund (UNICEF).
Kyrgyzstan	2012	Demographic and Health Survey (DHS)	ICF International, Ministry of Health (Kyrgyzstan), National Statistical Committee of the Kyrgyz Republic. Kyrgyzstan Demographic and Health Survey 2012. Fairfax, United States of America: ICF International.
Kyrgyzstan	2014	Multiple Indicator Cluster Survey (MICS)	National Statistical Committee of the Kyrgyz Republic, United Nations Children's Fund (UNICEF). Kyrgyzstan Multiple Indicator Cluster Survey 2014. New York, United States of America: United Nations Children's Fund (UNICEF), 2015.
Lao People's Democratic Republic	2006	Multiple Indicator Cluster Survey (MICS)	United Nations Children's Fund (UNICEF), Department of Statistics (Laos), Ministry of Health (Laos). Laos Multiple Indicator Cluster Survey 2006. New York, United States: United Nations Children's Fund (UNICEF). Â
Lao People's Democratic Republic	2012	Multiple Indicator Cluster Survey (MICS), Demographic and Health Survey (DHS)	Ministry of Education and Sports (Laos), Ministry of Health (Laos), Ministry of Planning and Investment (Laos). Laos Multiple Indicator Cluster Survey 2011-2012. New York, United States of America: United Nations Children's Fund (UNICEF), 2013.
Lao People's Democratic Republic	2017	Multiple Indicator Cluster Survey (MICS), Demographic and Health Survey (DHS)	Lao Statistics Bureau, Ministry of Education and Sports (Laos), Ministry of Health (Laos), United Nations Children's Fund (UNICEF). Laos Multiple Indicator Cluster Survey 2017. New York, United States of America: United Nations Children's Fund (UNICEF), 2018.
Lesotho	2014	Demographic and Health Survey (DHS)	ICF International, Ministry of Health and Social Welfare (Lesotho). Lesotho Demographic and Health Survey 2014. Fairfax, United States of America: ICF International.
Lesotho	2018	Multiple Indicator Cluster Survey (MICS)	Bureau of Statistics (Lesotho), United Nations Children's Fund (UNICEF). Lesotho Multiple Indicator Cluster Survey 2018. New York, United States of America: United Nations Children's Fund (UNICEF), 2019.
Macedonia	2005	Multiple Indicator Cluster Survey (MICS)	State Statistical Office (Macedonia) and United Nations Children's Fund (UNICEF). Macedonia Multiple Indicator Cluster Survey 2005. New York, United States: United Nations Children's Fund (UNICEF).
Macedonia	2011	Multiple Indicator Cluster Survey (MICS)	Institute of Public Health (Macedonia), Ipsos Strategic Puls, Ministry of Education and Science (Macedonia), Ministry of Labor and Social Policy (Macedonia), United Nations Children's Fund (UNICEF). Macedonia Multiple Indicator Cluster Survey 2011. New York, United States: United Nations Children's Fund (UNICEF), 2013.
Madagascar	2012	Multiple Indicator Cluster Survey (MICS)	National Institute of Statistics (Madagascar), United Nations Children's Fund (UNICEF). Madagascar - South Multiple Indicator Cluster Survey 2012. New York, United States of America: United Nations Children's Fund (UNICEF), 2015.
Madagascar	2018	Multiple Indicator Cluster Survey (MICS)	National Institute of Statistics (Madagascar), United Nations Children's Fund (UNICEF). Madagascar Multiple Indicator Cluster Survey 2018. 2019.
Malawi	2006	Multiple Indicator Cluster Survey (MICS)	United Nations Children's Fund (UNICEF), National Statistics Office (Malawi). Malawi Multiple Indicator Cluster Survey 2006. New York, United States: United Nations Children's Fund (UNICEF).
Malawi	2014	Multiple Indicator Cluster Survey (MICS)	National Statistical Office of Malawi, United Nations Children's Fund (UNICEF). Malawi Multiple Indicator Cluster Survey 2013-2014. New York, United States of America: United Nations Children's Fund (UNICEF), 2015.
Malawi	2016	Demographic and Health Survey (DHS)	Emory University and Centers for Disease Control & Prevention Collaboration, ICF International, Ministry of Health (Malawi), National Statistical Office of Malawi. Malawi Demographic and Health Survey 2015-2016. Fairfax, United States of America: ICF International, 2017.
Maldives	2017	Demographic and Health Survey (DHS)	ICF International, Ministry of Health (Maldives). Maldives Demographic and Health Survey 2016-2017. Fairfax, United States: ICF International, 2019.
Mali	2013	Demographic and Health Survey (DHS)	ICF International, INFO-STAT (Mali), Ministry of Health (Mali), National Institute of Statistics (INSTAT) (Mali), Planning and Statistics Unit, Ministry of Health (Mali). Mali Demographic and Health Survey 2012-2013. Fairfax, United States of America: ICF International, 2014.
Mali	2015	Multiple Indicator Cluster Survey (MICS)	Ministry of Health (Mali), Ministry of Planning (Mali), National Institute of Statistics (INSTAT) (Mali), United Nations Children's Fund (UNICEF). Mali Multiple Indicator Cluster Survey 2015. New York, United States of America: United Nations Children's Fund (UNICEF), 2017.

Mauritania	2007	Multiple Indicator Cluster Survey (MICS)	National Office of Statistics (Mauritania), United Nations Children's Fund (UNICEF). Mauritania Multiple Indicator Cluster Survey 2007. New York, United States of America: United Nations Children's Fund (UNICEF).
Mauritania	2011	Multiple Indicator Cluster Survey (MICS)	National Office of Statistics (Mauritania), United Nations Children's Fund (UNICEF). Mauritania Multiple Indicator Cluster Survey 2011. New York, United States of America: United Nations Children's Fund (UNICEF), 2015.
Mauritania	2015	Multiple Indicator Cluster Survey (MICS)	National Office of Statistics (Mauritania), United Nations Children's Fund (UNICEF). Mauritania Multiple Indicator Cluster Survey 2015. New York, United States of America: United Nations Children's Fund (UNICEF), 2018.
Mongolia	2005	Multiple Indicator Cluster Survey (MICS)	National Statistical Office of Mongolia, United Nations Children's Fund (UNICEF). Mongolia Multiple Indicator Cluster Survey 2005. New York, United States of America: United Nations Children's Fund (UNICEF).
Mongolia	2010	Multiple Indicator Cluster Survey (MICS)	National Statistical Office of Mongolia, United Nations Children's Fund (UNICEF). Mongolia Multiple Indicator Cluster Survey 2010. New York, United States of America: United Nations Children's Fund (UNICEF), 2013.
Mongolia	2012	Multiple Indicator Cluster Survey (MICS)	National Statistical Office of Mongolia, Statistics Department of Khuvsgul Aimag (Mongolia), United Nations Children's Fund (UNICEF). Mongolia - Khuvsgul Multiple Indicator Cluster Survey 2012. New York, United States of America: United Nations Children's Fund (UNICEF), 2015.
Mongolia	2013	Multiple Indicator Cluster Survey (MICS)	Government of Mongolia, National Statistical Office of Mongolia, United Nations Children's Fund (UNICEF), United Nations Population Fund (UNFPA). Mongolia Multiple Indicator Cluster Survey 2013. New York, United States of America: United Nations Children's Fund (UNICEF), 2016.
Mongolia	2018	Multiple Indicator Cluster Survey (MICS)	Government of Mongolia, National Statistical Office of Mongolia, United Nations Children's Fund (UNICEF), United Nations Population Fund (UNFPA). Mongolia Multiple Indicator Cluster Survey 2018. New York, United States of America: United Nations Children's Fund (UNICEF), 2019.
Montenegro	2006	Multiple Indicator Cluster Survey (MICS)	United Nations Children's Fund (UNICEF), Statistical Office of Montenegro. Montenegro Multiple Indicator Cluster Survey 2005. New York, United States: United Nations Children's Fund (UNICEF).
Montenegro	2013	Multiple Indicator Cluster Survey (MICS)	Statistical Office of Montenegro. Montenegro Multiple Indicator Cluster Survey 2013. New York, United States: United Nations Children's Fund (UNICEF), 2015.
Mozambique	2009	Multiple Indicator Cluster Survey (MICS)	United Nations Children's Fund (UNICEF), National Statistics Institute (Mozambique). Mozambique Multiple Indicator Cluster Survey 2008-2009. New York, United States: United Nations Children's Fund (UNICEF).
Mozambique	2011	Demographic and Health Survey (DHS)	ICF Macro, Manhica Health Research Center (CISM), Ministry of Health (Mozambique), National Institute of Statistics (INE) (Mozambique). Mozambique Demographic and Health Survey 2011. Fairfax, United States of America: ICF International.
Myanmar	2010	Multiple Indicator Cluster Survey (MICS)	Ministry of Health (Myanmar), Ministry of National Planning and Economic Development (Myanmar), United Nations Children's Fund (UNICEF). Myanmar Multiple Indicator Cluster Survey 2009-2010.
Namibia	2000	Demographic and Health Survey (DHS)	Central Statistics Office (Namibia), Macro International, Inc, Ministry of Health and Social Services (Namibia). Namibia Demographic and Health Survey 1992. Fairfax, United States: ICF International.
Nepal	2010	Multiple Indicator Cluster Survey (MICS)	United Nations Children's Fund (UNICEF), Central Bureau of Statistics (Nepal). Nepal Multiple Indicator Cluster Survey 2010. New York, United States: United Nations Children's Fund (UNICEF).
Nepal	2011	Demographic and Health Survey (DHS)	ICF Macro, Ministry of Health and Population (Nepal), New ERA. Nepal Demographic and Health Survey 2011. Fairfax, United States of America: ICF International.
Nepal	2014	Multiple Indicator Cluster Survey (MICS)	Central Bureau of Statistics (Nepal), United Nations Children's Fund (UNICEF). Nepal Multiple Indicator Cluster Survey 2014. New York, United States of America: United Nations Children's Fund (UNICEF), 2015.
Nepal	2017	Demographic and Health Survey (DHS)	ICF International, Ministry of Health (Nepal), New ERA. Nepal Demographic and Health Survey 2016-2017. Fairfax, United States of America: ICF International, 2017.
Nicaragua	2012	Other	Ministry of Health (Nicaragua), National Institute for Development Information (Nicaragua). Nicaragua National Demographic and Health Survey 2011-2012. Managua, Nicaragua: National Institute for Development Information (Nicaragua).
Nigeria	2000	Demographic and Health Survey (DHS)	Federal Office of Statistics (Nigeria), Institute for Resource Development, Macro Systems. Nigeria Demographic and Health Survey 1990. Fairfax, United States: ICF International.

Nigeria	2005	Nigeria Reproductive Health, Child Health, and Education Household, School, and Health Facility Survey	MEASURE Evaluation Project, Carolina Population Center, University of North Carolina, Center for Research, Evaluation, and Resource Development (CRERD), Center for Communication Programs, Bloomberg School of Public Health, Johns Hopkins, Creative Associates International, Constella Futures, Adolescent Health and Information Project (Nigeria), Federation of Muslim Women's Associations of Nigeria (FOMWAN), Nigerian Medical Association, Management Sciences for Health (MSH), Civil Society Action Coalition on Education For All. Nigeria Reproductive Health, Child Health, and Education Household, School, and Health Facility Baseline Surveys 2005. Chapel Hill, United States: MEASURE Evaluation Project, Carolina Population Center, University of North Carolina.
Nigeria	2007	Nigeria Reproductive Health, Child Health, and Education Household, School, and Health Facility Survey	MEASURE Evaluation Project, Carolina Population Center, University of North Carolina, Center for Research, Evaluation, and Resource Development (CRERD), Center for Communication Programs, Bloomberg School of Public Health, Johns Hopkins, Creative Associates International, Constella Futures, Adolescent Health and Information Project (Nigeria), Federation of Muslim Women's Associations of Nigeria (FOMWAN), Nigerian Medical Association, Management Sciences for Health (MSH), Civil Society Action Coalition on Education For All. Nigeria Reproductive Health, Child Health, and Education Household, School, and Health Facility Midline Surveys 2007. Chapel Hill, United States: MEASURE Evaluation Project, Carolina Population Center, University of North Carolina.
Nigeria	2007	Multiple Indicator Cluster Survey (MICS)	United Nations Children's Fund (UNICEF), National Bureau of Statistics (Nigeria). Nigeria Multiple Indicator Cluster Survey 2007. New York, United States: United Nations Children's Fund (UNICEF).
Nigeria	2011	Multiple Indicator Cluster Survey (MICS)	National Bureau of Statistics (Nigeria), United Nations Children's Fund (UNICEF). Nigeria Multiple Indicator Cluster Survey 2011. New York, United States of America: United Nations Children's Fund (UNICEF), 2013.
Nigeria	2017	Multiple Indicator Cluster Survey (MICS)	National Agency for the Control of AIDS (Nigeria), National Bureau of Statistics (Nigeria), National Primary Health Care Development Agency (NPHCDA) (Nigeria), United Nations Children's Fund (UNICEF). Nigeria Multiple Indicator Cluster Survey with National Immunization Coverage Survey Supplement 2016-2017. New York, United States of America: United Nations Children's Fund (UNICEF), 2018.
Pakistan	2010	Multiple Indicator Cluster Survey (MICS)	Government of Balochistan (Pakistan), United Nations Children's Fund (UNICEF). Pakistan - Balochistan Multiple Indicator Cluster Survey 2010. New York, United States of America: United Nations Children's Fund (UNICEF).
Pakistan	2011	Multiple Indicator Cluster Survey (MICS)	Bureau of Statistics Punjab (Pakistan), United Nations Children's Fund (UNICEF), United Nations Development Programme (UNDP). Pakistan - Punjab Multiple Indicator Cluster Survey 2011. New York, United States of America: United Nations Children's Fund (UNICEF), 2013.
Pakistan	2013	Demographic and Health Survey (DHS)	ICF International, National Institute of Population Studies (Pakistan), Pakistan Bureau of Statistics. Pakistan Demographic and Health Survey 2012-2013. Fairfax, United States of America: ICF International.
Pakistan	2014	Multiple Indicator Cluster Survey (MICS)	Bureau of Statistics, Planning and Development Department, Government of Sindh (Pakistan), Global Alliance for Improved Nutrition (GAIN), Pakistan Council of Research in Water Resource (PCRWR), United Nations Children's Fund (UNICEF). Pakistan - Sindh Multiple Indicator Cluster Survey 2014. Fairfax, United States of America: ICF International, 2016.
Pakistan	2014	Multiple Indicator Cluster Survey (MICS)	Bureau of Statistics Punjab (Pakistan), United Nations Children's Fund (UNICEF). Pakistan - Punjab Multiple Indicator Cluster Survey 2014. New York, United States of America: United Nations Children's Fund (UNICEF), 2015.
Pakistan	2018	Demographic and Health Survey (DHS)	ICF International, Ministry of National Health Services, Regulations & Coordination (Pakistan), National Institute of Population Studies (Pakistan). Pakistan Demographic and Health Survey 2017-2018. Fairfax, United States of America: ICF International, 2018.
Palestine	2007	Pan Arab Project for Family Health (PAPFAM)	League of Arab States, Palestinian Central Bureau of Statistics, United Nations Children's Fund (UNICEF). Palestine Family Health Survey 2006-2007.
Palestine	2010	Multiple Indicator Cluster Survey (MICS)	Ministry of Health (Palestine), Palestinian Central Bureau of Statistics, United Nations Children's Fund (UNICEF), United Nations Population Fund (UNFPA). Palestine Multiple Indicator Cluster Survey 2010. New York, United States of America: United Nations Children's Fund (UNICEF), 2014.
Palestine	2014	Multiple Indicator Cluster Survey (MICS)	Ministry of Health (Palestine), Palestinian Central Bureau of Statistics, United Nations Children's Fund (UNICEF). Palestine Multiple Indicator Cluster Survey 2014. New York, United States of America: United Nations Children's Fund (UNICEF), 2015.

Panama	2013	Multiple Indicator Cluster Survey (MICS)	National Institute of Statistics and Census (Panama), United Nations Children's Fund (UNICEF). Panama Multiple Indicator Cluster Survey 2013. New York, United States of America: United Nations Children's Fund (UNICEF).
Papua New Guinea	2007	Asian Development Bank Demographic and Health Survey (ADB DHS)	National Statistical Office (Papua New Guinea), National Statistics Office (Philippines). Papua New Guinea Demographic and Health Survey 2006-2007.
Paraguay	2008	Reproductive Health Survey (RHS)	Paraguay Center for Population Studies (CEPEP). Paraguay Reproductive Health Survey 2008. Asunción, Paraguay: Paraguayan Center for Population Studies (CEPEP).
Paraguay	2016	Multiple Indicator Cluster Survey (MICS)	General Directorate of Statistics, Surveys and Censuses (Paraguay), Ministry of Public Health and Social Welfare (Paraguay), United Nations Children's Fund (UNICEF). Paraguay Multiple Indicator Cluster Survey 2016. New York, United States of America: United Nations Children's Fund (UNICEF), 2017.
Peru	2008	Demographic and Health Survey (DHS)	Ministry of Economy and Finance (Peru), National Institute of Statistics and Informatics (Peru), ORC Macro. Peru Continuous Demographic and Health Survey 2003-2008. Fairfax, United States of America: ICF International.
Peru	2009	Demographic and Health Survey (DHS)	National Institute of Statistics and Informatics (Peru), ORC Macro. Peru Continuous Demographic and Health Survey 2009. Fairfax, United States of America: ICF International.
Peru	2010	Demographic and Health Survey (DHS)	National Institute of Statistics and Informatics (Peru). Peru Continuous Demographic and Health Survey 2010. Fairfax, United States of America: ICF International.
Peru	2011	Demographic and Health Survey (DHS)	Macro International, Inc, National Institute of Statistics and Informatics (Peru). Peru Continuous Demographic and Health Survey 2011. Fairfax, United States of America: ICF International.
Peru	2012	Demographic and Health Survey (DHS)	Macro International, Inc, National Institute of Statistics and Informatics (Peru). Peru Continuous Demographic and Health Survey 2012. Fairfax, United States of America: ICF International.
Peru	2013	Demographic and Health Survey (DHS)	ICF International, National Institute of Statistics and Informatics (Peru). Peru Continuous Demographic and Health Survey 2013 - INEI. Lima, Peru: National Institute of Statistics and Informatics (Peru), 2014.
Peru	2015	Peru Demographic and Family Health Survey (ENDES)	National Institute of Statistics and Informatics (Peru). Peru Demographic and Family Health Survey 2015. Lima, Peru: National Institute of Statistics and Informatics (Peru), 2017.
Peru	2016	Peru Demographic and Family Health Survey (ENDES)	National Institute of Statistics and Informatics (Peru). Peru Demographic and Family Health Survey 2016. Lima, Peru: National Institute of Statistics and Informatics (Peru), 2017.
Peru	2017	Peru Demographic and Family Health Survey (ENDES)	National Center for Food and Nutrition, National Institute of Health (Peru), National Institute of Statistics and Informatics (Peru), National Police of Peru (PNP). Peru Demographic and Family Health Survey 2017. Lima, Peru: National Institute of Statistics and Informatics (Peru).
Philippines	2013	Demographic and Health Survey (DHS)	ICF International, Philippines Statistics Authority. Philippines Demographic and Health Survey 2013. Fairfax, United States of America: ICF International, 2014.
Philippines	2017	Demographic and Health Survey (DHS)	ICF International, Philippines Statistics Authority, United States Agency for International Development (USAID). Philippines Demographic and Health Survey 2017. Fairfax, United States of America: ICF International, 2018.
Republic of Moldova	2012	Multiple Indicator Cluster Survey (MICS)	Ministry of Health (Moldova), National Bureau of Statistics (Moldova), United Nations Children's Fund (UNICEF). Moldova Multiple Indicator Cluster Survey 2012. New York, United States of America: United Nations Children's Fund (UNICEF), 2015.
Samoa	2009	Demographic and Health Survey (DHS)	ICF Macro, Ministry of Health (Samoa), Samoa Bureau of Statistics. Samoa Demographic and Health Survey 2009.
Sao Tome and Principe	2006	Multiple Indicator Cluster Survey (MICS)	National Institute of Statistics (Sao Tome and Principe), United Nations Children's Fund (UNICEF). Sao Tome and Principe Multiple Indicator Cluster Survey 2006. New York, United States: United Nations Children's Fund (UNICEF).
Sao Tome and Principe	2014	Multiple Indicator Cluster Survey (MICS), Demographic and Health Survey (DHS)	Global Fund to Fight Aids Tuberculosis and Malaria (GFATM), ICF International, National Center for Endemic Diseases (CNE) (Sao Tome and Principe), National Institute of Statistics (Sao Tome and Principe), United Nations Children's Fund (UNICEF), United Nations Development Programme (UNDP). Sao Tome and Principe Multiple Indicator Cluster Survey 2014. New York, United States of America: United Nations Children's Fund (UNICEF), 2016.
Senegal	2016	Multiple Indicator Cluster Survey (MICS)	National Agency of Statistics and Demography (Senegal), United Nations Children's Fund (UNICEF). Senegal - Dakar Urban Multiple Indicator Cluster Survey 2015-2016. New York, United States of America: United Nations Children's Fund (UNICEF), 2018.

Senegal	2017	Demographic and Health Survey (DHS)	ICF International, Ministry of Health and Social Action (Senegal), National Agency of Statistics and Demography (Senegal), Unit for the Fight Against Malnutrition (Senegal). Senegal Continuous Demographic and Health Survey 2017. Fairfax, United States of America: ICF International, 2018.
Serbia	2006	Multiple Indicator Cluster Survey (MICS)	United Nations Children's Fund (UNICEF), Statistical Office of the Republic of Serbia (SORS), Strategic Marketing Research Agency (SMMRI). Serbia Multiple Indicator Cluster Survey 2005-2006. New York, United States: United Nations Children's Fund (UNICEF).
Serbia	2010	Multiple Indicator Cluster Survey (MICS)	Statistical Office of the Republic of Serbia, United Nations Children's Fund (UNICEF). Serbia Multiple Indicator Cluster Survey 2010. New York, United States: United Nations Children's Fund (UNICEF).
Sierra Leone	2005	Multiple Indicator Cluster Survey (MICS)	United Nations Children's Fund (UNICEF), Statistics Sierra Leone. Sierra Leone Multiple Indicator Cluster Survey 2005. New York, United States: United Nations Children's Fund (UNICEF).
Sierra Leone	2010	Multiple Indicator Cluster Survey (MICS)	Statistics Sierra Leone, United Nations Children's Fund (UNICEF). Sierra Leone Multiple Indicator Cluster Survey 2010. New York, United States of America: United Nations Children's Fund (UNICEF).
Sierra Leone	2017	Multiple Indicator Cluster Survey (MICS)	Statistics Sierra Leone, United Nations Children's Fund (UNICEF). Sierra Leone Multiple Indicator Cluster Survey 2017. New York, United States of America: United Nations Children's Fund (UNICEF), 2018.
Somalia	2006	Multiple Indicator Cluster Survey (MICS)	Pan Arab Project for Family Health (PAPFAM), United Nations Children's Fund (UNICEF). Somalia Multiple Indicator Cluster Survey 2006. New York, United States of America: United Nations Children's Fund (UNICEF).
Somalia	2011	Multiple Indicator Cluster Survey (MICS)	Ministry of National Planning and Development (Somaliland), United Nations Children's Fund (UNICEF). Somalia - Somaliland Multiple Indicator Cluster Survey 2011. New York, United States of America: United Nations Children's Fund (UNICEF), 2015.
Somalia	2011	Multiple Indicator Cluster Survey (MICS)	Puntland Ministry of Planning and International Cooperation (Somalia), United Nations Children's Fund (UNICEF). Somalia - Northeast Zone Multiple Indicator Cluster Survey 2011. New York, United States of America: United Nations Children's Fund (UNICEF), 2015.
South Africa	2016	Demographic and Health Survey (DHS)	Department of Health (South Africa), ICF International, South African Medical Research Council, Statistics South Africa. South Africa Demographic and Health Survey 2016. Fairfax, United States of America: ICF International, 2019.
South Sudan	2010	Multiple Indicator Cluster Survey (MICS)	Central Bureau of Statistics (Sudan), Ministry of Health (South Sudan). Sudan - North Multiple Indicator Cluster Survey 2010. New York, United States of America: United Nations Children's Fund (UNICEF), 2015.
Sudan	2010	Multiple Indicator Cluster Survey (MICS)	Central Bureau of Statistics (Sudan), Federal Ministry of Health (Sudan), Government of Sudan, Ministry of Health (South Sudan), Southern Sudan Centre for Census, Statistics and Evaluation. Sudan - South Multiple Indicator Cluster Survey 2010. New York, United States of America: United Nations Children's Fund (UNICEF), 2015.
Sudan	2014	Multiple Indicator Cluster Survey (MICS)	Central Bureau of Statistics (Sudan), Federal Ministry of Health (Sudan), United Nations Children's Fund (UNICEF). Sudan Multiple Indicator Cluster Survey 2014. New York, United States of America: United Nations Children's Fund (UNICEF), 2016.
Suriname	2006	Multiple Indicator Cluster Survey (MICS)	General Statistical Office (Suriname), United Nations Children's Fund (UNICEF). Suriname Multiple Indicator Cluster Survey 2006. New York, United States of America: United Nations Children's Fund (UNICEF).
Suriname	2010	Multiple Indicator Cluster Survey (MICS)	General Bureau of Statistics (Suriname), Ministry of Planning and Development Cooperation (Suriname), Ministry of Social Affairs and Housing (Suriname), United Nations Children's Fund (UNICEF). Suriname Multiple Indicator Cluster Survey 2010. New York, United States of America: United Nations Children's Fund (UNICEF), 2013.
Suriname	2018	Multiple Indicator Cluster Survey (MICS)	General Bureau of Statistics (Suriname), Ministry of Planning and Development Cooperation (Suriname), Ministry of Social Affairs and Housing (Suriname), United Nations Children's Fund (UNICEF). Suriname Multiple Indicator Cluster Survey 2018. New York, United States of America: United Nations Children's Fund (UNICEF), 2019.
Swaziland	2010	Multiple Indicator Cluster Survey (MICS)	Central Statistical Office (Swaziland), United Nations Children's Fund (UNICEF). Swaziland Multiple Indicator Cluster Survey 2010. New York, United States of America: United Nations Children's Fund (UNICEF).
Swaziland	2014	Multiple Indicator Cluster Survey (MICS)	Central Statistical Office (Swaziland), United Nations Children's Fund (UNICEF), United Nations Educational, Scientific and Cultural Organization (UNESCO), United Nations Population Fund (UNFPA). Swaziland Multiple Indicator Cluster Survey 2014. New York, United States of America: United Nations Children's Fund (UNICEF), 2016.

Syrian Arab Republic	2001	Pan Arab Project for Family Health (PAPFAM)	Central Bureau of Statistics (Syria), League of Arab States. Syria Family Health Survey 2001.
Syrian Arab Republic	2006	Multiple Indicator Cluster Survey (MICS)	United Nations Children's Fund (UNICEF), Central Bureau of Statistics (Syria), Ministry of Health (Syria), Pan Arab Project for Family Health (PAPFAM). Syria Multiple Indicator Cluster Survey 2006. New York, United States: United Nations Children's Fund (UNICEF).
Tajikistan	2005	Multiple Indicator Cluster Survey (MICS)	United Nations Children's Fund (UNICEF), State Committee on Statistics of the Republic of Tajikistan. Tajikistan Multiple Indicator Cluster Survey 2005. New York, United States: United Nations Children's Fund (UNICEF).
Tajikistan	2012	Demographic and Health Survey (DHS)	ICF International, Ministry of Health (Tajikistan), Statistical Agency under the President of the Republic of Tajikistan. Tajikistan Demographic and Health Survey 2012. Fairfax, United States of America: ICF International, 2013.
Tajikistan	2017	Demographic and Health Survey (DHS)	ICF International, Statistical Agency under the President of the Republic of Tajikistan. Tajikistan Demographic and Health Survey 2017. Fairfax, United States of America: ICF International, 2018.
Thailand	2006	Multiple Indicator Cluster Survey (MICS)	National Statistical Office (Thailand), United Nations Children's Fund (UNICEF). Thailand Multiple Indicator Cluster Survey 2005-2006. New York, United States of America: United Nations Children's Fund (UNICEF).
Thailand	2012	Multiple Indicator Cluster Survey (MICS)	College of Population Studies, Chulalongkorn University (Thailand), Institute for Population and Social Research, Mahidol University (Thailand), International Health Policy Program (Thailand), Ministry of Education (Thailand), Ministry of Public Health (Thailand), Ministry of Social Development and Human Security (MSDHS) (Thailand), National Health Security Office (Thailand), National Statistical Office (Thailand), Thai Health Promotion Foundation, United Nations Children's Fund (UNICEF). Thailand Multiple Indicator Cluster Survey 2012. New York, United States of America: United Nations Children's Fund (UNICEF), 2016.
Thailand	2016	Multiple Indicator Cluster Survey (MICS)	National Statistical Office (Thailand), United Nations Children's Fund (UNICEF). Thailand 14 Provinces Multiple Indicator Cluster Survey 2015-2016.
Thailand	2016	Multiple Indicator Cluster Survey (MICS)	National Health Security Office (Thailand), National Statistical Office (Thailand), United Nations Children's Fund (UNICEF). Thailand Multiple Indicator Cluster Survey 2015-2016. New York, United States of America: United Nations Children's Fund (UNICEF), 2018.
Thailand	2016	Multiple Indicator Cluster Survey (MICS)	National Health Security Office (Thailand), National Statistical Office (Thailand), United Nations Children's Fund (UNICEF). Thailand - Bangkok Small Community Multiple Indicator Cluster Survey 2016. New York, United States of America: United Nations Children's Fund (UNICEF), 2018.
Togo	2006	Multiple Indicator Cluster Survey (MICS)	Directorate General of Statistics and National Accounting (Togo), United Nations Children's Fund (UNICEF). Togo Multiple Indicator Cluster Survey 2006. New York, United States: United Nations Children's Fund (UNICEF).
Togo	2010	Multiple Indicator Cluster Survey (MICS)	Directorate General of Statistics and National Accounting (Togo), United Nations Children's Fund (UNICEF). Togo Multiple Indicator Cluster Survey 2010. New York, United States: United Nations Children's Fund (UNICEF).
Trinidad and Tobago	2006	Multiple Indicator Cluster Survey (MICS)	Central Statistical Office (Trinidad and Tobago) and United Nations Children's Fund (UNICEF). Trinidad and Tobago Multiple Indicator Cluster Survey 2006. New York, United States: United Nations Children's Fund (UNICEF).
Tunisia	2012	Multiple Indicator Cluster Survey (MICS)	Ministry of Regional Development and Planning (Tunisia), National Institute of Statistics (Tunisia), United Nations Children's Fund (UNICEF). Tunisia Multiple Indicator Cluster Survey 2011-2012. New York, United States of America: United Nations Children's Fund (UNICEF), 2014.
Turkey	2004	Demographic and Health Survey (DHS)	Institute of Population Studies, Hacettepe University, Ministry of Health (Turkey). Turkey Demographic and Health Survey 2003-2004. Fairfax, United States: ICF International.
Turkmenistan	2006	Multiple Indicator Cluster Survey (MICS)	Ministry of Foreign Affairs (Turkmenistan), Ministry of Health and Medical Industry (Turkmenistan), National Institute of State Statistics and Information (Turkmenistan), United Nations Children's Fund (UNICEF). Turkmenistan Multiple Indicator Cluster Survey 2006. New York, United States of America: United Nations Children's Fund (UNICEF), 2016.
Turkmenistan	2016	Multiple Indicator Cluster Survey (MICS)	State Committee on Statistics of Turkmenistan, United Nations Children's Fund (UNICEF). Turkmenistan Multiple Indicator Cluster Survey 2015-2016. New York, United States of America: United Nations Children's Fund (UNICEF), 2017.
Uganda	2011	Demographic and Health Survey (DHS)	ICF Macro, Uganda Bureau of Statistics. Uganda Demographic and Health Survey 2011. Fairfax, United States of America: ICF International.

Uganda	2015	Gavi Full Country Evaluations (FCE) Project	Institute for Health Metrics and Evaluation (IHME), Infectious Diseases Research Collaboration (IDRC). Uganda Gavi FCE Household Survey 2015. Seattle, United States: Institute for Health Metrics and Evaluation (IHME), 2017.
Ukraine	2012	Multiple Indicator Cluster Survey (MICS)	StatInform Consulting, State Statistics Service (Ukraine), Ukrainian Center for Social Reforms (UCSR), United Nations Children's Fund (UNICEF). Ukraine Multiple Indicator Cluster Survey 2012. New York, United States of America: United Nations Children's Fund (UNICEF), 2014.
United Republic of Tanzania	2000	Demographic and Health Survey (DHS)	Bureau of Statistics (Tanzania), Macro International, Inc, Ministry of Health (Tanzania). Tanzania Demographic and Health Survey 1991-1992. Fairfax, United States: ICF International.
United Republic of Tanzania	2016	Demographic and Health Survey (DHS)	ICF International, Ministry of Health (Zanzibar), Ministry of Health, Community Development, Gender, Elderly and Children (MoHCDEC) (Tanzania), National Bureau of Statistics (Tanzania), Office of the Chief Government Statistician (OCGS) (Zanzibar). Tanzania Demographic and Health Survey 2015-2016. Fairfax, United States of America: ICF International, 2016.
Uzbekistan	2006	Multiple Indicator Cluster Survey (MICS)	United Nations Children's Fund (UNICEF), State Committee of the Republic of Uzbekistan on Statistics. Uzbekistan Multiple Indicator Cluster Survey 2006. New York, United States: United Nations Children's Fund (UNICEF).
Vanuatu	2008	Multiple Indicator Cluster Survey (MICS)	Ministry of Health (Vanuatu), United Nations Children's Fund (UNICEF). Vanuatu Multiple Indicator Cluster Survey 2007-2008. New York, United States: United Nations Children's Fund (UNICEF).
Viet Nam	2006	Multiple Indicator Cluster Survey (MICS)	General Statistics Office (Vietnam), United Nations Children's Fund (UNICEF). Vietnam Multiple Indicator Cluster Survey 2006. New York, United States of America: United Nations Children's Fund (UNICEF).
Viet Nam	2011	Multiple Indicator Cluster Survey (MICS)	General Statistics Office (Vietnam), United Nations Children's Fund (UNICEF). Vietnam Multiple Indicator Cluster Survey 2010-2011. New York, United States of America: United Nations Children's Fund (UNICEF).
Viet Nam	2014	Multiple Indicator Cluster Survey (MICS)	General Statistics Office (Vietnam), United Nations Children's Fund (UNICEF). Vietnam Multiple Indicator Cluster Survey 2013-2014. New York, United States of America: United Nations Children's Fund (UNICEF), 2015.
Yemen	2003	Pan Arab Project for Family Health (PAPFAM)	Central Statistical Organization (Yemen), League of Arab States, Ministry of Public Health and Population (Yemen), Pan Arab Project for Family Health (PAPFAM). Yemen Family Health Survey 2003.
Yemen	2006	Multiple Indicator Cluster Survey (MICS)	Ministry of Health (Yemen) and United Nations Children's Fund (UNICEF). Yemen Multiple Indicator Cluster Survey 2006. New York, United States: United Nations Children's Fund (UNICEF).
Yemen	2013	Demographic and Health Survey (DHS)	Central Statistical Organization (Yemen), ICF International, Ministry of Public Health and Population (Yemen). Yemen Demographic and Health Survey 2013. Fairfax, United States of America: ICF International.
Zambia	2008	Global Fund Household Health Coverage Survey	Central Statistical Office (Zambia). Zambia Global Fund Household Health Coverage Survey 2008. Lusaka, Zambia: Central Statistical Office (Zambia).
Zimbabwe	2014	Multiple Indicator Cluster Survey (MICS)	United Nations Children's Fund (UNICEF), Zimbabwe National Statistics Agency. Zimbabwe Multiple Indicator Cluster Survey 2014. New York, United States of America: United Nations Children's Fund (UNICEF), 2015.

Table S1: Surveys included in the antibiotic usage model

1.2 Polygon resampling

The methods for polygon resampling are depicted in Figure S4. For each polygon we cropped a raster of the WorldPop 2010 global population estimates,[1] with a cell area of 5x5km at the equator, to that area (Figure S4a). We sampled 10,000-point locations (latitude and longitude of the cell centroid) from each polygon, with a sampling probability proportional to the cell population (Figure S4b). *K*-means clustering was then applied to the point locations, so that there was one cluster per 1,000 raster cells within the polygon. Each cluster was then assigned an integration weight proportional of the number of point locations that were geographically closer to this cluster than any other (Figure S4c). The sum of the weights of the point locations for each polygon resampled was equal to one.

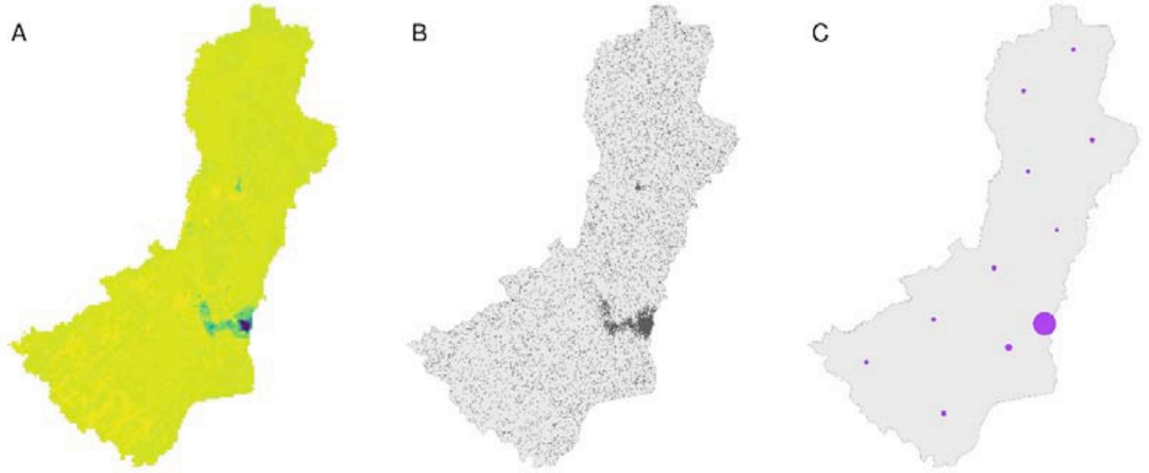

Figure S4: Polygon resampling. A pictorial representation of polygon resampling methods using k-means clustering; a) a raster of the total population cropped to the polygon to be resampled; b) 10,000 point locations sampled with a probability based on the cell population; c) k-means clustering applied to the point locations in (b), the size of the point represents the integration weight. (Figure from Golding *et al.* 2017.[2]

1.3 Data preparation

The resampled, weighted point locations were linked to the data for that polygon and added to the data linked to actual point locations which were assigned a weight of one; this was then cleaned to be used in the geospatial model. Data were collapsed to one grouped observation per point location. We calculated the numerator as the number of children reported to have received antibiotics and the denominator as the number of children with information on antibiotic use. We used the caregiver reported symptoms of cough, rapid/difficulty breathing, chest symptoms and fever in the previous two weeks as our standard definition of LRI. However, respiratory symptoms varied between surveys and included children with the following combinations of symptoms: a) cough, b) cough and rapid/difficulty breathing, c) cough and fever, d) cough and rapid/difficulty breathing and fever, OR e) cough, rapid/difficulty breathing and chest symptoms. To maximise the amount of data and ensure that all surveys were comparable, we adjusted the proportion of children using antibiotics in each of these slightly differing denominator groups to be equivalent to the proportion of children using antibiotics for LRI.

For each survey we calculated the proportion of children using antibiotics for each combination of symptoms. We then tested the linear relationship between the proportion of antibiotic use in children with LRI (using our standard definition) to the proportion of antibiotic use in each of the other combinations of symptoms, using Pearson correlation coefficient. The correlation was over 0.89 r^2 for each pairwise comparisons (Table S2), confirming a strong linear relationship between antibiotic use for each combination of symptoms. We then used linear regression models to calculate the relationship between the proportion of antibiotic use in children with LRI and the proportion of antibiotic use in each of the other combinations of symptoms. The coefficients of these models (Table S2) were then used to adjust the collapsed data.

Coefficient	Denominator				
	cough	cough, rapid/difficulty breathing & chest symptoms	cough, rapid/difficulty breathing and fever	cough and rapid/difficulty breathing	cough and fever
correlation (r^2)	0.89	0.93	0.91	0.92	0.9
Intercept	0.053	-0.002	0.036	0.021	0.038
beta	1.107	1.018	1.077	1.086	1.050

Table S2: LRI adjustment model coefficients. Pearson's correlation coefficients and linear regression coefficients for relationship between the proportion of antibiotic use in children with LRI (standard definition) to those with slightly differing combinations of symptoms

2. Antibiotic usage model

A two stage Bayesian binomial hierarchal model was fit to estimate the probability of antibiotic usage for each 5x5km pixel for 127 LMICs. The model was fit separately for each region (Figure S5) to improve computational stability, incorporate epidemiological differences between regions, and to allow modelling/assessing differing effects and powers of covariates.

For Maldives, Mauritius, North Korea, Nauru, Tuvalu, American Samoa and Dominica, estimates of antibiotic usage were not produced using the geostatistical model as these countries/territories either have small populations (Nauru, Tuvalu, Dominica and American Samoa have populations of less than 100,000 people) and limited data; are located a long distance from neighbouring countries (Maldives and Mauritius), or have no accurate covariate data (North Korea) meaning that the suitability of the geostatistical model is limited. For these countries, the median of the nationally aggregated estimates for those regions were imputed as the antibiotic usage proportion for all pixels in the same region. Additionally, China, Russia and Lebanon were not included in this model and for the purpose of this study were designated as high-income countries; although classified as upper-middle income by the World Bank, the data sources available in these countries were most suitable for the high-income analysis.

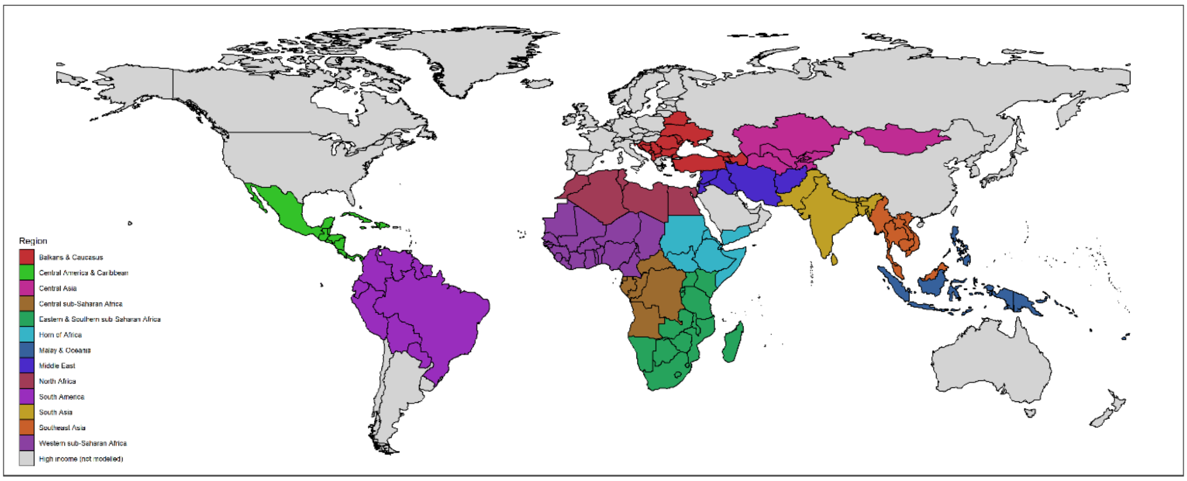

Figure S5: Geographical regions for the model of antibiotic use. Although not modelled, North Korea was considered part of Southeast Asia; American Samoa, Nauru and Tuvalu as part of Malay and Oceania, Dominica as part of Central America and the Caribbean, and Mauritius and the Maldives as part of South Asia.

2.1 Covariate selection

Covariates to inform the model of antibiotic usage were selected based on biological plausibility and importance in the model. Covariates were checked for correlation using the Pearson correlation coefficient, one of any pair of covariates with an $r^2 > 0.8$ were dropped. The selected covariates are displayed in Table S3.

Covariate name	Covariate Source
Travel time to nearest settlement >50,000 inhabitants	Weiss DJ, Nelson A, Gibson HS. et al. A global map of travel time to cities to assess inequalities in accessibility in 2015. *Nature* 2018; **533**(7688):333-336. doi: 10.1038/nature25181
Ratio of dependants to working-age adults	Lloyd CT, Sorichetta A, Tatem AJ. High resolution global gridded data for use in population studies. *Sci Data* 2017; **4**(170001). doi: 10.1038/sdata.2017.1. Available from: http://www.worldpop.org.uk/data/get_data/
Distance to rivers and lakes	Natural Earth. Rivers and lake centerlines dataset. Available at: http://www.naturalearthdata.com/downloads/10mphysical-vectors/10m-rivers-lake-centerlines/. (Accessed: 24th July 2017)
DPT3 vaccine coverage	Produced by the local burden of disease team at the Institute of Health Metrics and evaluation, contact Simon Hay (sihay@uw.edu) for further information
Malaria incidence	Bhatt S, Weiss DJ, Cameron E, et al. The effect of malaria control on Plasmodium falciparum in Africa between 2000 and 2015. *Nature* 2015; **526**(7572)**:**207–211. doi: 10.1038/nature15535
Gross domestic product (GDP) per pixel	Kummu, M., Taka, M., & Guillaume, J. H. A. Data Descriptor: Gridded global datasets for Gross Domestic Product and Human Development Index over 1990-2015. *Sci Data* 2018; **6**(5):180004. doi: 10.1038/sdata.2018.4.
Urbanicity	Pesaresi M, Ehrlich D, Ferri S et al. Operating procedure for the production of the Global Human Settlement Layer from Landsat data of the epochs 1975, 1990, 2000, and 2014. JRC Technical Report EUR 27741 EN. doi:10.2788/253582 (online)
Years of maternal education	Graetz N, Friedman J, Osgood-Zimmerman A. et al. Mapping local variation in educational attainment across Africa. *Nature* 2018; **555**:48–53. https://doi.org/10.1038/nature25761 (2018).
Ambient air pollution, particulate matter 2.5	Data integration model available at: https://arxiv.org/abs/1609.00141
Population of children under 5	Lloyd C, Sorichetta A, & Tatem A. High resolution global gridded data for use in population studies. *Sci Data* 2017; **4**(170001). doi: 10.1038/sdata.2017.1. Available at: http://www.worldpop.org.uk/data/get_data/
Mean temperature	Harris I, Jones PD, Osborn TJ, Lister DH. Updated high-resolution grids of monthly climatic observations – the CRU TS3.10 dataset. *Int J Climatol* 2014; **34:** 623–642
Childhood stunting	Produced by the local burden of disease team at the Institute of Health Metrics and evaluation, contact Simon Hay (sihay@uw.edu) for further information
Normalised difference vegetation index	NASA & NOAA. Advanced Very High Resolution Radiometer (AVHRR) Normalized Difference Vegetation Index (NDVI) dataset. Available at: https://nex.nasa.gov/nex/projects/1349/. (Accessed: 25th July 2017)
Latitude	The latitude of each pixel calculated by the local burden of disease team at the Institute of Health Metrics and evaluation.
Health Access and Quality Index (HAQI)	Barber, RM. et al. Healthcare Access and Quality Index based on mortality from causes amenable to personal health care in 195 countries and territories, 1990–2015: a novel analysis from the Global Burden of Disease Study 2015. *Lancet* 2017; **390**(10091): 231 – 266. doi: 10.1016/S0140-6736(17)30818-8

Table S3: Covariates included in the model of antibiotic usage

Covariates were normalised by subtracting the mean from each value and dividing them by the standard deviation. This results in all covariates having a mean of 0 and a standard deviation of 1. This allows for the comparison of regression coefficients across multiple covariates as the regression coefficients will appear on the same scale, increasing the ease of interpretation. Normalising covariates is considered important in penalised regression models. Final covariate selection was based on those informing the models with the best predictive validity (models were run with five-fold cross-validation and covariates from models with the best out-of-sample metrics were selected for the final model. The covariates used for each of the modelling regions are shown in Figure S6

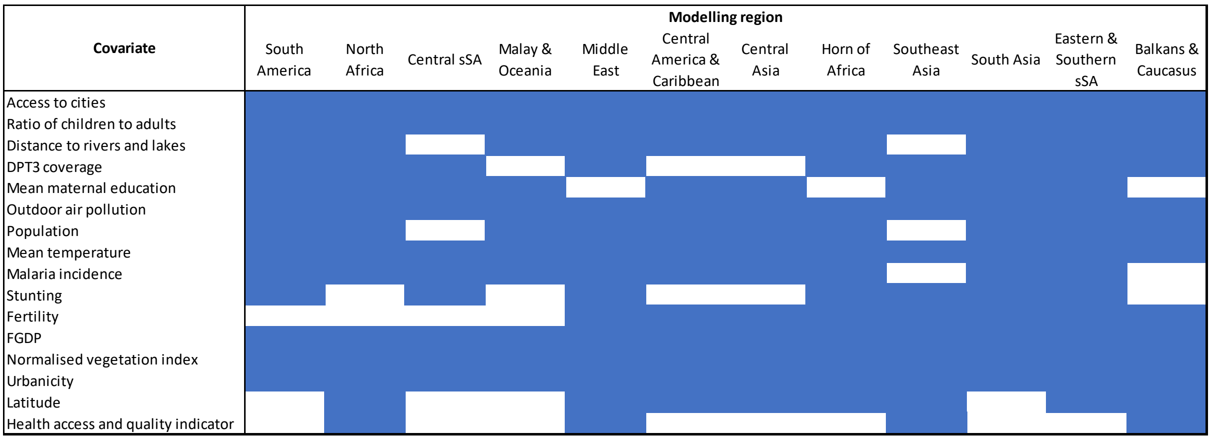

Figure S6. Covariates included in the model of antibiotic usage for each modelling regions

2.2 Stacked ensemble model

A stacked ensemble model was fit to the data and the selected explanatory covariates, following the methodology of Bhatt et al.[3] For each region we fit three child models: boosted regression trees (BRT), generalised additive models (GAM) and elastic net (E-net, a penalised regression model) using five-fold cross-

validation to produce out-of-sample predictions for each datapoint. These estimates were then used as the explanatory covariates in the subsequent geostatistical model. This modelling strategy is beneficial as it aids in covariate selection, captures potential non-linear effects and accounts for interactions between covariates, improving the predictive power over single models alone.

2.3 Geostatistical model

2.3.1 Model specification

We fit a spatially and temporally explicit binomial Bayesian hierarchal generalised linear model (GLM) to estimate the proportion of antibiotic usage in each 5x5km pixel in our study area. We modelled the logit probability of antibiotic use, p_i, using a GLM with the out-of-sample predictions from the stacked ensemble model, χ_i- used as the explanatory covariates with the coefficients, $\boldsymbol{\beta}$, constrained to sum up to one. We included a term for the residual spatial and temporal error, ϵ_{GP}; an independent nugget effect, ϵ_i; a Gaussian country level random effect, ϵ_C, to allow for differences in antibiotic provision, policies and political effects; and a Gaussian survey level random effect, ϵ_S, to account for biases and reporting inaccuracies (this final random effect was included in model fitting but not in prediction).

The residual spatial and temporal error was modelled as a three-dimensional Gaussian process with a covariance matrix as the Kronecker product of spatial covariance ($\boldsymbol{K}_{\text{space}}$) and temporal covariance ($\boldsymbol{K}_{\text{time}}$). Spatial covariance was modelled as a stationary Matérn function and temporal covariance as an autoregressive function of order 1 (AR1).

$$N_i^+ \sim Binomial(p_i, N_i)$$

$$logit(p_i) = \alpha + \boldsymbol{X}_i\boldsymbol{\beta} + \epsilon_C + \epsilon_S + \epsilon_i + \epsilon_{GP}$$

$$\epsilon \sim GP\left(0, \boldsymbol{K}_{space} \otimes \boldsymbol{K}_{time}\right)$$

$$\boldsymbol{K}_{space} = (2^{\nu-1}\Gamma(\nu))^{-1}(\kappa\boldsymbol{D})^{\nu}K_{\nu}(\kappa\boldsymbol{D})$$

$$\boldsymbol{K}_{time_{k,l}} = \rho^{|t_k - t_l|}$$

2.3.2 Model priors

We included priors on each of the random effects, these varied slightly by region and were selected to produce the models with the best predictive performance. We selected the following standard priors based on iterations of the model run and resulting in the highest predictive validity and most plausible trends in antibiotic use:

- $\epsilon_c = loggamma(\alpha = 2, \gamma = 0.05)$
- $\epsilon_i = loggamma(\alpha = 2, \gamma = 0.05)$
- $\epsilon_s = loggamma(\alpha = 2, \gamma = 0.05)$
- $\theta_{2t} = N(\mu = 2.5, \sigma^2 = 1/1.21)$

ϵ_c was changed to $loggamma(\alpha = 2, \gamma = 1)$ for North Africa and Malay and Oceania, and to $loggamma(\alpha = 2, \gamma = 4)$ for Western sub-Saharan Africa.

For the spatial hyperparameter (κ and τ) we used the multivariate, uncorrelated normal priors automatically selected by R-INLA, based on the finite elements spatial mesh.

2.3.3 Spatial Mesh creation

A finite element spatial mesh is required to fit the stochastic partial differential equation (SPDE) approximation, used to model the spatial-temporal error. We used smoothed polygon boundaries for the modelled regions as templates for the meshes. We created inner triangles for the mesh with a maximum edge length set to 0.25 degrees and the outer buffer triangles maximum edge length set to 5 degrees. Figure S7 shows the mesh for the Western sub-Saharan Africa region. The spatial mesh was uniform for all modelled regions.

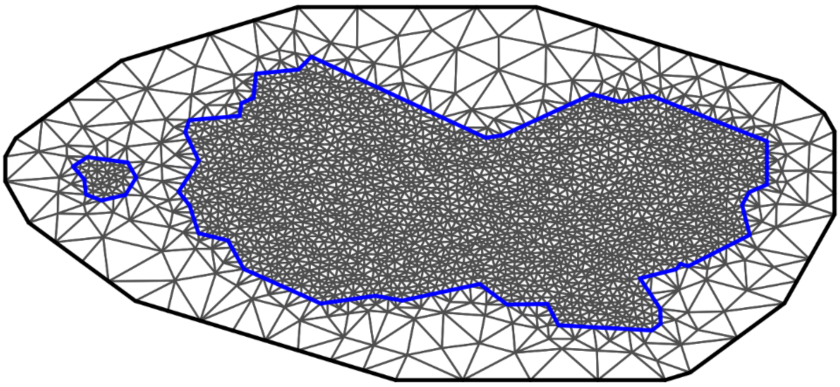

Figure S7: Finite element mesh for the Western sub-Saharan Africa region.

2.3.4 Model fitting

The models were fit using the Stochastic partial differential equation (SPDE)[4] approach using Integrated Nested Laplace Approximations (R-INLA version 18.07.12) in the R statistical software environment version 3.5.0. Laplace Approximations were used to model the spatial-temporal error. We took 1000 draws of the model posterior then calculated the mean and 95% uncertainty intervals for the probability of antibiotic use for each pixel. Additionally, we aggregated the draw level estimates to the district, state and national level, calculating the mean and 95% uncertainty intervals for these locations.

2.4 Model validation

To assess the predictive validity of the model (i.e. understanding how well the model predicts to locations with no data), we performed five-fold cross validation and calculated out-of-sample predictive metrics (RMSE and r^2). We assigned included surveys to one of five random folds; we then created five datasets holding out one-fold of data from each dataset and fit the model on each of these datasets using the final model specifications. This creates an out-of-sample prediction for each datapoint. We then calculated the r^2 and RMSE of the input data points and the mean out-of-sample prediction to show the predictive validity of the model.

Our dataset contains relatively rare binomial outcomes with small sample sizes, meaning there are an abundance of antibiotic use proportions in the survey data at 0, 0.5 and 1. These are not realistic probabilities, but an artefact of the small sample sizes. To compare our model estimates at the pixel level would therefore be uninformative as, due to these factors, even if the data were modelled perfectly it may not be captured by the model validation as we cannot retrieve the true probability from the raw data. To account for these nuances, we aggregated the data to the national level, to stabilise the estimates, and investigated the predictive performance of the model against this data.

3. Antibiotic consumption data preparation

We quantified antibiotic consumption using defined daily doses (DDD), a standardized World Health Organization (WHO) metric. We used the latest version of the Anatomical Therapeutic Chemical Classification (ATC)/DDD guideline and index.[5,6] DDDs are defined as *'the assumed average maintenance dose per day for a drug used for its main indication in adults',*[5] allowing for a standardized comparison of antibiotic consumption between countries and years.

The 2019 version included updated DDD values for nine antimicrobials and routes of administration: ampicillin (from 2g to 6g), oral amoxicillin (from 1g to 1.5g), parenteral amoxicillin (from 1g to 3g), oral amoxicillin with beta-lactamase inhibitor (from 1g to 1.5g), parenteral temocillin (from 2g to 4g), cefepime (from 2g to 4.5g), meropenem (from 2g to 3g), parenteral ciprofloxacin (from 0.5g to 0.8g), and parenteral colistin (from 3g to 9g), for a better reflection of recommended and prescribed dosages.[7]

3.1 IQVIA data processing

We obtained two datasets on antibiotic consumption from IQVIA, one for the years 2000-2013 and one for 2014-2018. IQVIA conduct national surveys and audits and calculate the total sales of each antibiotic preparation by country and year. IQVIA provided data from 76 countries and territories; data were aggregated for Central America (Costa Rica, El Salvador, Guatemala, Honduras, Nicaragua and Panama) and French West

Africa (Benin, Burkina Faso, Côte d'Ivoire, Cameroon, Congo, Gabon, Guinea, Mali, Senegal and Togo). Data for China and Hong Kong were provided separately. Data are available by the channel in which they were sold; hospital or retail.

For the 2000-2013 dataset antibiotic sales were provided in 'standard units' for each preparation of each antibiotic (i.e. one record for each available brand/product of each antibiotic). A standard unit is defined as one dose of the preparation and is often one tablet/capsule or one 1ml or 5ml dose of parenteral antibiotic. The weight of antibiotic in each preparation and the definition of the standard unit were provided from IQVIA. Each preparation was matched to the ATC level 5 code of the antibiotic, based on the molecule name. The total weight of antibiotic, in kilograms, in each preparation was calculated by multiplying the weight of antibiotic by the number of standard units. This was then aggregated to the total quantity in kilograms of each antibiotic (ATC level 5 code) by country, year and channel (hospital/retail).

For the 2014-2018 dataset, the total weight in kilograms for each antibiotic that was sold through retail and hospital channels were provided for each country and year. We limited the dataset to antibiotics for systemic use (J01) and then matched each substance to its ATC level 5 code and combined this with the 2000-2013 dataset. For antibiotics combined with salts, diluents or probiotics the additional substances were considered to have a negligible contribution to the total weight (i.e. those substances were assigned to the ATC level 5 code of the antibiotic).

The total DDDs for each antibiotic stratified by country and year were calculated using the WHO methodology.[5,6] This methodology provides a value, in grams, which is considered the DDD for each antibiotic (Table S4). We divided the total sales (in kilograms) per year for each antibiotic by the assigned DDD value, to obtain DDDs per year.

$$Total\ DDDs = \frac{Kg\ of\ antibiotics/1000}{WHO\ DDD\ value\ (g)}$$

For combination antibiotics without specific ATC level 5 codes and DDD values, the DDDs were calculated and split between the component antibiotics, based on the standard preparations of the combination substances. In cases were the oral and parenteral DDD values differed we used expert opinion to select the suitable DDD (Table S4b).

We expressed the rate of antibiotic consumption as DDDs per 1000 population per year (DDD/1000/year) for each antibiotic using the population estimates from the Global Burden of Disease (GBD) 2019 study for each country and year in our dataset (available from http://ghdx.healthdata.org/). For French West Africa and Central America, the sum of the populations from all included countries were used to calculate the DDDs/1000 population.

3.2 IQVIA missing data imputation

IQVIA collected data from different 'channels', specified as retail and hospital channels. For the majority of countries, data on both channels was available, however, some countries had retail data only. We used multiple imputation to fill in these missing values, using the classification and regression trees (CART) method from the 'mice' package version 3·7·0,[8] in R version 3·6·1.

MICE, Multivariate Imputation by Chained Equations, is a method for dealing with missing data which is based on Fully Conditional Specification (FCS).[9] Each incomplete variable is imputed by a separate model with multiple iterations generated by Gibbs sampling, producing plausible values for each target variable based on a set of predictor variables within the dataset. For this implementation the predictor variables were super-region, region, country, year, ATC3 code, ATC4 code and retail DDD per 1000 population (where complete). The classification and regression trees algorithm was selected for use as this produced the most realistic values when trialling imputation methods.

We ran the multiple imputation algorithm 10 times, with 100 iterations each to impute the missing DDD per 1000 per year values for the missing antibiotics and channels. The imputed values were squeezed to be within the existing range of DDD per 1000 per year for that ATC level 3 group of antibiotics. We then calculated the total J01 DDD per 1000 per year for each country and year, as well as the total DDD per 1000 per year for each group of antibiotics at ATC level 3 (J01A tetracyclines; J01B amphenicols; J01C beta-lactam antibacterials - penicillins; J01D other beta-lactam antibacterials; J01E sulfonamides and trimethoprim; J01F macrolides,

lincosamides and streptogramins; J01G aminoglycosides; J01M quinolones), and separately for each AWaRe category (Access, Watch, Reserve, the designation of antibiotics to each of these categories is shown in Table S5)[10]

Antibiotic	Daily Defined Dose (grams)	Antibiotic	Daily Defined Dose (grams)
**acetylspiramycin**	3	**flomoxef**	2
**amikacin**	1	**flucloxacillin**	3
**amoxicillin**	1.5	**flumequine**	1.2
**amoxicillin & beta-lactamase inhibitor**	1.5	**fosfomycin**	3
**ampicillin**	6	**fusidic acid**	1.5
**ampicillin & beta-lactamase inhibitor**	6	**garenoxacin**	0.4
**arbekacin**	0.2	**gatifloxacin**	0.4
**azidocillin**	1.5	**gemifloxacin**	0.32
**azithromycin**	0.3	**gentamicin**	0.24
**azlocillin**	12	**imipenem & cilastatin**	2
**aztreonam**	4	**isepamicin**	0.4
**bacampicillin**	1.2	**josamycin**	2
**benzathine phenoxymethylpenicillin**	2	**kanamycin**	1
**biapenem**	1.2	**latamoxef**	4
**carbenicillin**	12	**levofloxacin**	0.5
**cefaclor**	1	**lincomycin**	1.8
**cefadroxil**	2	**linezolid**	1.2
**cefalexin**	2	**lomefloxacin**	0.4
**cefalotin**	4	**lymecycline**	0.6
**cefamandole nafate**	6	**meropenem**	3
**cefatrizine**	1	**meropenem & vaborbactam**	3
**cefazedone**	3	**metacycline**	0.6
**cefazolin**	3	**metronidazole**	1.5
**cefbuperazone**	2	**mezlocillin**	6
**cefcapene pivoxil**	0.45	**midecamycin**	1
**cefdinir**	0.6	**minocycline**	0.2
**cefditoren pivoxil**	0.4	**moxifloxacin**	0.4
**cefepime**	4	**nafcillin**	3
**cefetamet pivoxil**	1	**neomycin**	1
**cefixime**	0.4	**netilmicin**	0.35
**cefmenoxime**	2	**norfloxacin**	0.8
**cefmetazole**	4	**ofloxacin**	0.4
**cefminox**	4	**oleandomycin**	1
**cefodizime**	2	**ornidazole**	1
**cefonicid**	1	**oxacillin**	2

**cefoperazone**	4	**oxytetracycline**	1
**cefoperazone & beta-lactamase inhibitor**	4	**panipenem & betamipron**	2
**ceforanide**	4	**pazufloxacin**	1
**cefotaxime**	4	**pefloxacin**	0.8
**cefotaxime & beta-lactamase inhibitor**	4	**penicillin g**	3.6
**cefotetan**	4	**penicillin v**	2
**cefotiam**	4	**pheneticillin**	1
**cefoxitin**	6	**piperacillin**	14
**cefozopran**	4	**piperacillin & beta-lactamase inhibitor**	14
**cefpiramide**	2	**pivmecillinam**	0.6
**cefpirome**	4	**pristinamycin**	2
**cefpodoxime**	0.4	**prulifloxacin**	0.6
**cefprozil**	1	**ribostamycin**	1
**cefradine**	2	**rifabutin**	0.15
**cefroxadine**	2.1	**rifampicin**	0.6
**ceftaroline fosamil**	1.2	**rifamycin**	0.6
**ceftazidime & beta-lactamase inhibitor**	6	**roxithromycin**	0.3
**ceftazidime**	4	**rufloxacin**	0.2
**cefteram pivoxil**	0.4	**sitafloxacin**	0.1
**ceftezole**	3	**sparfloxacin**	0.2
**ceftibuten**	0.4	**spectinomycin**	3
**ceftizoxime**	4	**streptomycin**	1
**ceftolozane & beta-lactamase inhibitor**	3	**sulbactam**	1
**ceftriaxone**	2	**sulbenicillin**	15
**ceftriaxone & beta-lactamase inhibitor**	2	**sulfadiazine**	0.6
**cefuroxime**	0.5	**sulfadiazine & trimethoprim**	1
**chloramfenicol**	3	**sulfamethoxazole**	2
**chlortetracycline**	1	**sulfamethoxazole & trimethoprim**	1.92
**ciprofloxacin**	1	**sulfametrole & trimethoprim**	1.92
**clarithromycin**	0.5	**sultamicillin**	1.5
**clindamycin**	1.2	**tebipenem pivoxil**	0.56
**clofoctol**	1.5	**tedizolid**	0.2
**cloxacillin**	2	**teicoplanin**	0.4
**combination penicillins**	2	**telavancin**	0.7
**daptomycin**	0.27	**telithromycin**	0.8
**demeclocycline**	0.6	**temocillin**	2
**dibekacin**	0.14	**tetracycline**	1
**dicloxacillin**	2	**thiamphenicol**	1.5
**dirithromycin**	0.5	**ticarcillin & beta-lactamase inhibitor**	15
**doripenem**	1.5	**tigecycline**	0.1
**doxycycline**	0.1	**tinidazole**	1.5

**enoxacin**	0.8	**tobramycin**	0.24
**ertapenem**	1	**tosufloxacin**	0.45
**erythromycin**	1	**trimethoprim**	0.4
**faropenem**	0.75	**vancomycin**	2
**fleroxacin**	0.4		

Table S4a: DDD values for each included J01 antibiotic

**Antimicrobial**	**DDD_O(g)**	**DDD_P(g)**
Amoxicillin	**1.5**	3
Ampicillin	2	**6**
Azithromycin	**0.3**	0.5
Cefotiam	1.2	**4**
Cefuroxime	**0.5**	3
Ciprofloxacin	**1**	0.8
Clarithromycin	**0.5**	1
Clindamycin	**1.2**	1.8
Erythromycin	**1**	2
Fosfomycin	**3**	8
Linezolid	1.5	**1.2**
Tobramycin	0.3	**0.24**

Table S4b: DDD values for each included J01 antibiotic which differ for oral and parenteral preparations. Expert opinion was used to select the most suitable values, highlighted in bold in the table

**AWaRe Categorisation**	**Antibiotic**
**Access**	amikacin, amoxicillin & beta-lactamase inhibitor, amoxicillin, ampicillin & beta-lactamase inhibitor, ampicillin, azithromycin, bacampicillin, benzathine phenoxymethylpenicillin, cefadroxil, cefalexin, cephalothin, cefatrizine, cefazedone, cefazolin, cefbuperazone, cefmetazole, cefminox, cefonicid, cefotetan, cefoxitin, cefprozil, cefradine, cefroxadine, ceftezole, cefuroxime, chloramfenicol, clindamycin, cloxacillin, combination penicillins, dibekacin, dicloxacillin, doxycycline, flucloxacillin, gentamicin, isepamicin, kanamycin, lymecycline, metacycline, minocycline, nafcillin, neomycin, netilmicin, oxacillin, oxytetracycline, penicillin g, penicillin v, pheneticillin, spectinomycin, sulfadiazine & trimethoprim, sulfamethoxazole & trimethoprim, sulfametrole & trimethoprim, temocillin, tetracycline, thiamphenicol, ticarcillin & beta-lactamase inhibitor, tobramycin, trimethoprim
**Watch**	biapenem, cefaclor, cefamandole nafate, cefcapene pivoxil, cefdinir, cefditoren pivoxil, cefetamet pivoxil, cefixime, cefmenoxime, cefodizime, cefoperazone & beta-lactamase inhibitor , cefoperazone, cefotaxime & beta-lactamase inhibitor , cefotaxime, cefotiam, cefpiramide, cefpodoxime, ceftazidime , cefteram pivoxil, ceftibuten, ceftizoxime, ceftriaxone & beta-lactamase inhibitor , ceftriaxone, ciprofloxacin, clarithromycin, dirithromycin, doripenem, enoxacin, ertapenem, erythromycin, faropenem, flumequine, garenoxacin, gatifloxacin, gemifloxacin, imipenem & cilastatin, josamycin, levofloxacin, lomefloxacin, meropenem, midecamycin, moxifloxacin , norfloxacin, ofloxacin, panipenem & betamipron , pazufloxacin, pefloxacin, piperacillin & beta-lactamase inhibitor, piperacillin, pristinamycin, prulifloxacin, ribostamycin, roxithromycin, rufloxacin, sitafloxacin, sparfloxacin, spiramycin, tebipenem pivoxil, teicoplanin, telavancin, tosufloxacin, vancomycin
**Reserve**	aztreonam, cefepime, cefozopran, cefpirome, ceftaroline fosamil, ceftolozane & beta-lactamase inhibitor, daptomycin, fosfomycin, linezolid, meropenem & vaborbactam, tedizolid.

Table S5. AWaRe categorisation of J01 antibiotics[10]

3.3 WHO data and correlation between IQVIA Analytics and WHO estimates

WHO reported antibiotic consumption in DDD per 1000 per day for all J01 antibiotics and for each ATC level 3 class for 65 countries for either 2015 or 2016.[11] The estimates for 40 countries overlapped with the IQVIA dataset. To investigate whether the data were comparable we converted the WHO data to DDD per 1000 per year (by multiplying by 365) and plotted the values against the corresponding countries and years from the IQVIA dataset. We calculated the Pearson's correlation coefficient; there was a strong linear relationship between the IQVIA and WHO data, with an r^2 of 0.83 (Figure S8).

The WHO data for Austria, Czech Republic, Germany, New Zealand and Spain only covered antibiotic consumption from the community, excluding hospital use. We were unable to impute values for the missing data as the report did not provide consumption by sectors (hospital and community/retail). Consequentially, the IQVIA estimates for these countries are higher than those from WHO. Excluding these five countries, improved the Pearson correlation coefficient to r^2=0.88. Additionally, the WHO data for Jordan covered less than 70% of the population and was not population adjusted. When we removed Jordan as well as the five countries above from the dataset, the correlation coefficient increased to r^2=0.9.

In the WHO dataset, six countries included only data from the public sector (community/retail and hospital), these were Burundi, Bolivia, Costa Rica, Paraguay, Peru and Brunei. However, the results were similar to the IQVIA estimates, indicating that the public sector might make up the vast majority of sales in these countries, with the private sector having a more negligible role.

After accounting for these differences, the only remaining datapoint which was inconsistent between the IQVIA and WHO estimates was that for Brazil, with the WHO estimate being considerably higher. The IQVIA and WHO data for Brazil come from different sources with IQVIA being from an audit of retail antibiotic sales and WHO data being from manufacturers, however, neither organisation provides reasons for the variation in the estimates and no indication as to which is the more plausible value. Interestingly, the Center for Disease Dynamics, Economics and Policy (CDDEP) published relatively high estimates of antibiotic consumption in Brazil of 6,763 DDD/1000 population in 2015 (18.5 DDD/1000 population/day), based on IQVIA Midas data[12]. Due to this inconsistency, we decided to incorporate both the WHO estimates and IQVIA estimates into our model.

The WHO estimate for antimicrobial consumption for Mongolia (64.4 DDD/1,000 population/day) was noticeably higher than for surrounding countries. Studies in Mongolia have consistently shown overuse of antibiotics in both hospitals and the community at a large scale [13-15]; we therefore deemed there was no reason to suspect this data of being inaccurate and allowed our model to account for the spatial variation between Mongolia and neighbouring countries.

Based on the results of this comparison we were satisfied to combine both datasets of antibiotic consumption. We added the WHO data for countries that were not included in the IQVIA datasets. The IQVIA dataset covered more country-years and there are overlapping sources between the data making it inappropriate to include the data as multiple data points for each country, as this would be putting undue weight upon those data estimates in the subsequent models).

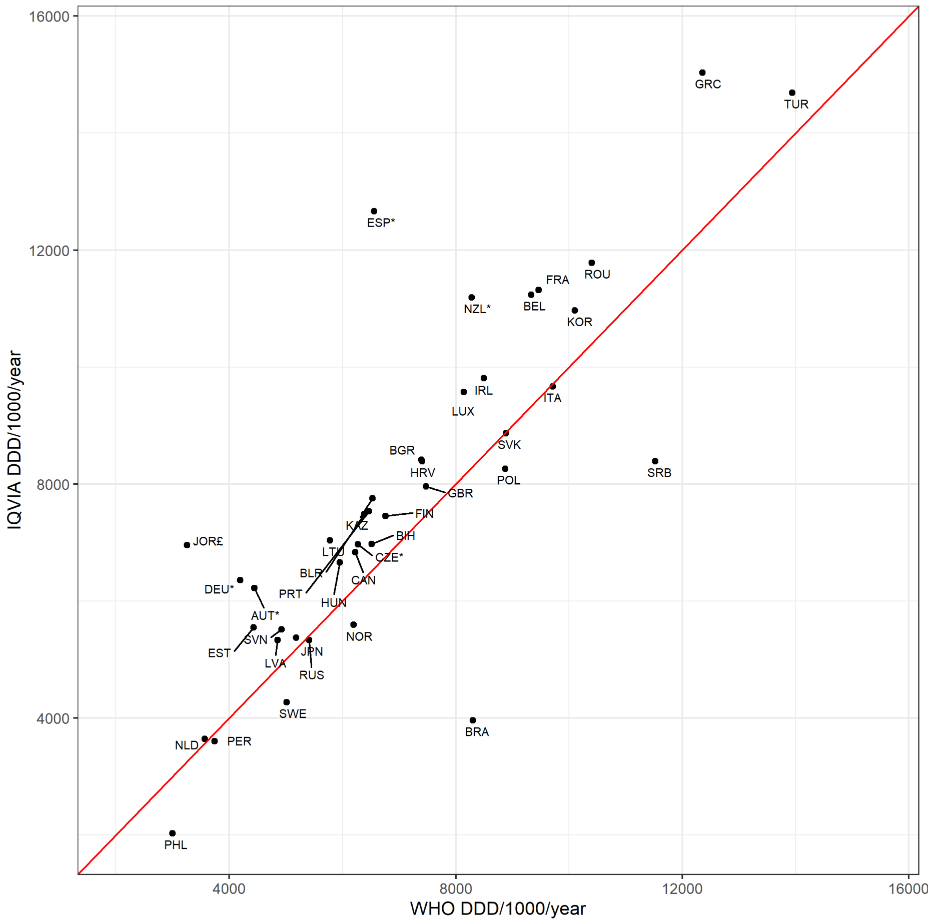

Figure S8: Comparison of IQVIA and WHO antibiotic consumption data. DDDs/1000 population/year from the IQVIA Analytics database on the y-axis and from the WHO report on surveillance of antibiotic consumption on the x-axis. This includes data from all country-years from both sources. The red line indicates a 1:1 relationship between the data (intercept = 0, slope = 1). Countries followed by * indicated that the WHO estimates are for community antibiotic consumption only. Countries followed by £ indicate that the WHO estimate is for <70% of the population and has not been population adjusted.

3.4 ESAC-NET data

The European Surveillance of Antimicrobial Consumption Network (ESAC-NET), managed by the European Centre for Disease Prevention and Control (ECDC), compiles data on antimicrobial consumption for 27 countries in the European Union (EU) and European Economic Area (EEA) for selected years between 1997 and 2018. Antibiotic consumption is provided by hospital and community sectors, or total for some countries, and is available for the total J01 class antimicrobials and at the ATC level 3.[16] Data were presented in DDDs/1000/day and were converted to DDD/1000/year. Not all countries reported both hospital and community sector antimicrobial consumption for all years. For country-years with missing data from either the hospital or retail sector we imputed the missing DDDs/1000/year using the CART multiple imputation method from the mice package in R, as detailed in section 3.1. If data were missing from both sectors for a specific year, we excluded this country-year from the analysis.

We visually compared the ESAC-NET data to the IQVIA data, by plotting the DDD/1000/year values for each country for a selected year (2015) (Figure S9). The Pearson's correlation coefficient for all the years was r^2 = 0.93, and for 2015 only it was r^2 =0.89. IQVIA data from Spain and Greece report high antibiotic consumption than the data for ECDC; reasons for this difference are not explored in existing publications. The IQVIA data was included in the model over the ESAC-NET data as this was more detailed and there is likely duplication between the two data sources meaning it was not suitable to include both.

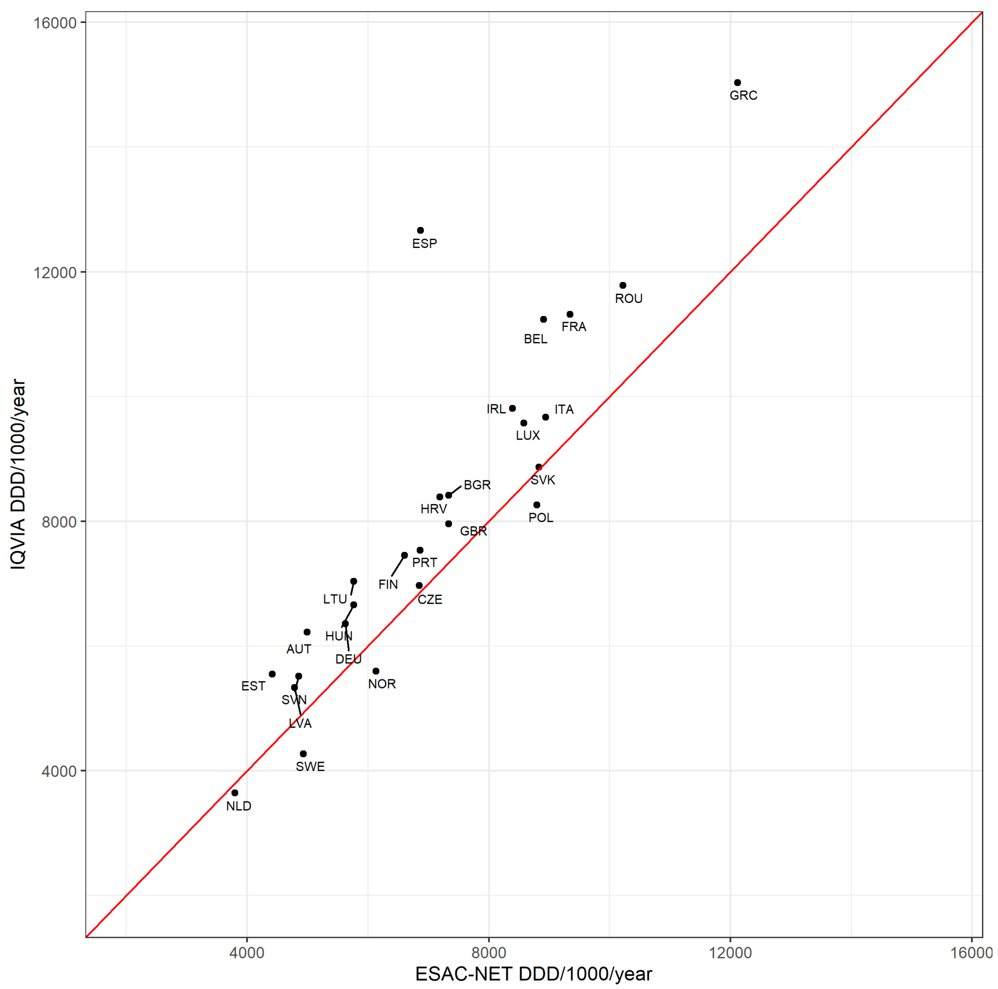

Figure S9: Comparison of IQVIA and the ESAC-NET antibiotic consumption dataset. All data are for 2015 and are presented in DDD/1000 population /year. The red line indicates a 1:1 relationship between the data (intercept = 0, slope = 1).

3.5 Combining data sources

Due to the IQVIA data being more detailed and comprehensive, with consumption data available for each individual antibiotic (provide ATC level) and both channels (hospital and retail/community sectors) for a larger number of countries, we considered this our gold standard dataset. Due to the high correlations between the estimates from WHO and ESAC-NET with the IQVIA data, data from WHO and ESAC-NET were used to supplement countries and years not covered by the IQVIA dataset.

We searched the published literature for additional antibiotic surveillance systems or research studies publishing nationally representative J01 antibiotic consumption data presented in DDDs/1000/year. We identified two further data sources, a research study from Samoa,[17] and one from Kenya;[18] these were added to the dataset, illustrating the paucity of nationally representative antibiotic consumption surveillance data, likely due to it being resource-intensive and expensive to collect such data, and is therefore rarely done in LMICs. It was only possible to gather this data for Samoa due to the small population of the country and the fact that it is an island, geographically isolated meaning that unregulated importation of antibiotics is restricted and it is possible to measure consumption without large personnel and infrastructure expenses.

Our final dataset consisted of antibiotics consumption data from 112 countries/territories, covering 47 HICs and 65 LMICs (Figure S10).

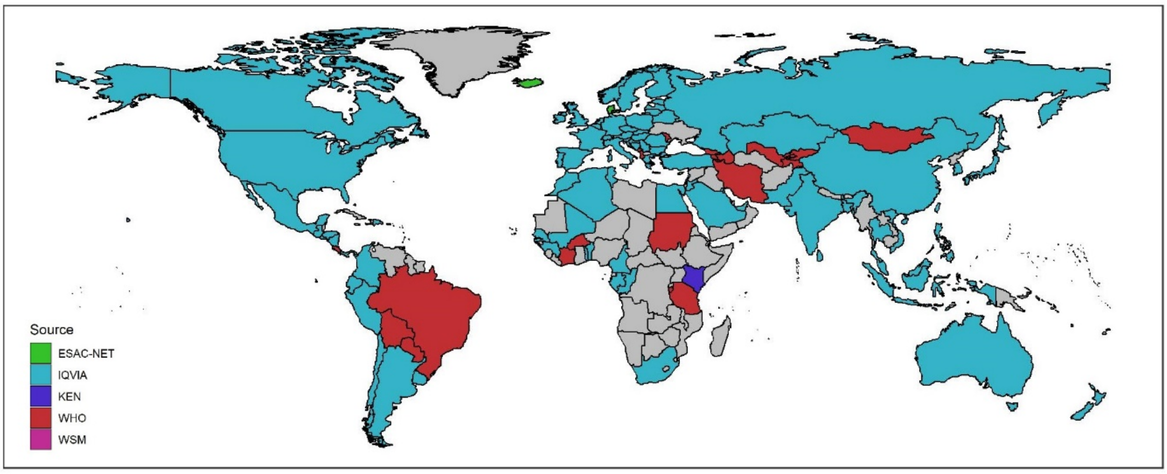

Figure S10. All data sources included in the final model of antibiotic consumption. KEN represents the published literature from Kenya[18]; WSM represents the published literature from Samoa.[17]

4. Antibiotic consumption model

High-income and low- and middle-income countries have differing patterns and drivers of antibiotic consumption. Due to this factor, and the differing quantity of data available, we modelled antibiotic consumption in HICs and LMICs separately. We split the data into two datasets, one containing the high-income countries as designated by the World Bank plus China, Russia and Lebanon; and one containing the low, lower-middle and upper-middle income countries, as designated by the World Bank, minus China, Russia and Lebanon. We decided to model China, Russia and Lebanon as part of the HICs as all three countries had a large amount of data available from IQVIA, were lacking in data for antibiotic use (from household surveys) and were considered to be irregular for the GBD regions in which they are located, based on the IQVIA data - Lebanon was considered to be more similar levels of antibiotic consumption in the raw data to Israel (an HIC) than to other LMICs in the region such as Syria and Iraq, we hypothesised that Lebanon's health and socio-demographic properties might be skewed due to the large number of refugees in the country. Due to this factor, we felt that the associations between health, sociodemographic covariates and antibiotic consumption observed in Lebanon was better incorporated into the model of antibiotic consumption for HICs. Similarly, Russia and China were considered dissimilar to other LMICs within their GBD regions, mainly due to the large geographic areas they cover, large populations and differing political systems. They were therefore considered more suited to the model of antibiotic consumption in HICs.

4.1 High income countries antibiotic consumption model specifications

Our HIC dataset contained data for 47 out of 69 countries deemed high-income. The countries without data were primarily small islands nations, nations with small populations and those on the Arabian Peninsula (data were not available for Andorra, Antigua and Barbuda, Bahamas, Bahrain, Barbados, Trinidad and Tobago, Bermuda, Cook Islands, Greenland, Guam, Israel, Monaco, Northern Mariana Islands, Niue, Oman, Palau, Qatar, San Marino, Seychelles, Saint Kitts and Nevis, Tokelau, and US Virgin Islands).

We selected existing covariates available from the GBD study (http://ghdx.healthdata.org/) with biological plausibility to influence antibiotic consumption. We trialled a stacked ensemble model (as detailed in section 4.2.2), to model the association between selected covariates, and combinations thereof, and the antibiotic consumption data. However, examining out-of-sample cross validation with the r^2 and RMSE statistics, we found no combination of covariates that could suitably predict antibiotics consumption in HICs. We therefore deemed a spatial and temporal model of antibiotic consumption unsuitable for HICs.

Due to these limitations, and the very few country-years requiring estimation of antibiotic consumption, we implemented a CART multiple imputation algorithm (as detailed in section 3.1) to predict antibiotic consumption for HICs without data for 2000-2018.

4.2 Low- and middle- income countries antibiotic consumption model specifications

The dataset for LMICs was sparser than for HICs, only covering 65 out of 135 LMICs. We therefore use a two-stage modelling technique to firstly capture the associations between selected socio-demographic and health related covariates, and then to smooth the estimates in time and space, borrowing strength from countries and years with more data to inform estimates for country-years without data. We firstly implemented a stacked ensemble model, and then used the out-of-sample estimates for this model in a Spatial-Temporal Gaussian Process Regression (ST-GPR) model to estimate the antibiotic consumption by country and year for 2000-2018, together with the associated uncertainty.

4.2.1 Covariate selection

We screened the available covariates in the GBD database for plausibility in driving antimicrobial consumption in LMICs. We selected 29 possible covariates and fitted a Lasso regression model to select the most informative covariates, i.e. the covariates which minimised the prediction error for antibiotic consumption the most. We then fit a Lasso regression model between the log of the antimicrobial consumption rate (DDD/1000/year) and the covariates.

The covariates with non-zero regression coefficients when lambda was set to the highest value possible within one standard deviation of the lambda for the best performing model (λ=0.2) were considered relevant. This covariate selection process secures that the model is not overly complex and reduces overfitting. The final covariates included in the model were: antibiotic use (results from the model specified in Sections 1 and 2), antenatal care coverage, hospital beds per 1000 persons, temperature, outdoor air pollution, sanitation, physicians per capita, health access and quality index, sociodemographic index (covariates available from http://ghdx.healthdata.org/).

Various transformations of the covariates plus centre-scaling were trialled to identity any preferable associations with the data. The only improvements to the model whilst fitting were by taking the log of hospital beds per 1000 persons, therefore all other covariates were used non-centre-scaled and with no transformations.

4.2.2 Stacked ensemble model

We fit a stacked ensemble model, following the general methodology of Bhatt *et al.*[3] We fit eight child models to the data and covariates, these were boosted regression trees (BRT), generalised linear models (GAM), neural nets, random forest, cubist and penalised regression (elastic net (e-net), ridge and lasso) models. The BRT, neural net, cubist and random forest were built with five-fold cross-validation and the model parameters were repeatedly fit from a grid of potential model parameters. The best performing model parameters were selected based on the model with the lowest RMSE. The models were fit using the CARET package version 6·085 in R. The GAM was fit with using the 'mgcv' package version 1·8·31 in R. The penalised regression models were fit (using the 'glmnet' package version 3·0·2 in R) with $\alpha = 0$ for ridge; $\alpha = 0.5$ for e-net and $\alpha = 1$ for lasso; for each model λ was selected as the lowest possible λ within one standard deviation of the λ for the best performing model. This allows for high predictive performance but restricts the model from being over-complicated.

Each of the final child models was fit with five-fold cross validation and the out-of-sample predictions were taken through to the next stage. We checked the pairwise correlation for each of the models using Pearson's correlation coefficient; where any pair of models had an r^2 over 0.8 than the model with the lowest predictive validity (lowest r^2 between the out-of-sample predictions and the observed data) was excluded. The final child models used in the ensemble were BRT, GAM, ridge and neural net, random forest and cubist.

The child models were then combined using a stacking model. Various options of stacking models were trialled, these included a linear regression, constrained weighted mean; a BRT, neural net or GAM. Based on superior out-of-sample performance we used a constrained weighted mean algorithm to stack the child models. The coefficients (weights) of each child model were calculated using quadratic programming and constrained to sum to one, these were then used to calculate the weighted mean of the child models for each country-year.

Using this final stacked ensemble model, we predicted the antibiotic consumption in DDD per 1000 per year for each LMIC for 2000-2018. These predictions were taken forward to the next stage of the model and will be hereafter referred to as the stage one model. All modelling for the stacked ensemble model was undertaken in R.

4.2.3 Spatial-Temporal Gaussian Process Regression
We used the out-of-sample predictions from the stacked ensemble model (our stage 1 model) for a Spatial-Temporal Gaussian Process Regression (ST-GPR). The ST-GPR is a modelling framework developed by the GBD study;[19] it is a stochastic model which easily captures flexible, non-linear trends over time. It firstly smooths the residuals of the stage one model based on spatial and temporal weights, then fits the trend using a Gaussian Process Regression (GPR). We followed the GBD methodology and code, running the model in Python.

To estimate residual variability of the stage 1 model, we firstly fit a locally weighted polynomial regression (LOESS) function, borrowing strength across space and time. Temporal weights determine how quickly the correlation between datapoints diminishes over time and are based on the scaled distance (in time) between two observations. Temporal weights are controlled by lambda (λ); a lower λ indicates high temporal correlation, higher temporal weights, and therefore smoother trends in time.

$$W_i = \frac{1}{e^{\lambda|t-t^0|}}$$

Spatial smoothing is determined by the GBD location hierarchy (Table S6). We calculated a single constant based on a vector of weights for each level of the location hierarchy, controlled by the parameter zeta, ζ. Varying weights were assigned based on the country's location in the hierarchy:

- Weights of residuals in the same country: ζ^0
- Weights of residuals in different countries in the same region: ζ^1
- Weights of residuals in different regions in the same super region: ζ^2
- Weights of residuals from other super regions: ζ^3

These weights work in a scaler fashion and ζ can be interpreted as the amount to downweight residuals from different countries within the same region, compared to datapoints from the same country. For example, when applying spatial weights to datapoint from Tanzania, if ζ= 0.1, then residuals from data in Kenya will be weighted 1/100 of the weight of those in Tanzania, a datapoint in Nigeria will be given 1/10,000 of the weight of those in Tanzania, whilst data from India will be given 1/1,000,000 of the weight. This means that a higher ζ translates to more spatial smoothing across locations more distant in the location hierarchy.

The spatial and temporal weights were combined by multiplying them and summing them across each level in the location hierarchy. The weights were then normalised for each time period. This allows the amount of spatial weight, $w_{i,j}$, assigned to a datapoint (in time and space) to vary based on the amount of data available at each time point and location hierarchy. The weights were then added back into the stage 1 model.

$$Stage\ 2 = Stage\ 1 + \sum residual_j * w_{i,j}$$

For the model of antibiotic consumption in LMICs, for locations with no data the smoothing parameters were set to $\lambda = 0.05$; $\zeta = 0.1$; for locations with one data point $\lambda = 0.05$; $\zeta = 0.05$; and for locations with more than one data point as $\lambda = 0.005$; $\zeta = 0.5$, indicating that as the data density increasing there is less spatial and temporal smoothing required. The model for the Oceania region was run with increased smoothing, $\lambda = 0.05$; $\zeta = 0.1$ as there was only one data point in the region which was from published literature instead of the gold standard datasets, and therefore less weight was assigned to this.

The GPR was then fit to the stage 2 model. This assumes the trend follows a Gaussian distribution defined by a mean function $m(.)$ and a covariance function $Cov(.)$. We use a Matérn-Euclidian covariance function controlled by three parameters; the differentiability, controlling the smoothness of the estimates; the scale, controlling how correlated the estimates are over time; and the amplitude, controlling how far the estimates can vary from the mean. We ran the GPR with the scale set to 5 and amplitude to 5. For Oceania, the GPR amplitude was set to 0 to restrict the weight given to the single datapoint.

We took 1000 draws of the GPR, predicting antibiotic consumption for each year and country in our study period as the mean of these draws. Additionally, we calculated 95% uncertainty as the 0.05 and 0.95 percentiles of the sampling distribution.

Level 0	Level 1 - Super Region	Level 2 - Region	Level 3 - Country	Income level
Global				
	Central Europe, Eastern Europe, and Central Asia			
		Central Asia		
			Armenia	Lower middle
			Azerbaijan	Upper middle
			Georgia	Lower middle
			Kazakhstan	Upper middle
			Kyrgyzstan	Lower middle
			Mongolia	Lower middle
			Tajikistan	Lower middle
			Turkmenistan	Upper middle
			Uzbekistan	Lower middle
		Central Europe		
			Albania	Upper middle
			Bosnia and Herzegovina	Upper middle
			Bulgaria	Upper middle
			Croatia	Upper middle
			Czech Republic	High
			Hungary	High
			Macedonia	Upper middle
			Montenegro	Upper middle
			Poland	High
			Romania	Upper middle
			Serbia	Upper middle
			Slovakia	High
			Slovenia	High
		Eastern Europe		
			Belarus	Upper middle
			Estonia	High
			Latvia	High
			Lithuania	High
			Moldova	Lower middle
			Russian Federation	High
			Ukraine	Lower middle
	High-income			
		Australasia		
			Australia	High
			New Zealand	High
		High-income Asia Pacific		
			Brunei	High
			Japan	High
			South Korea	High
			Singapore	High

	High-income North America		
		Canada	High
		Greenland	High
		United States	High
	Southern Latin America		
		Argentina	High
		Chile	High
		Uruguay	High
	Western Europe		
		Andorra	High
		Austria	High
		Belgium	High
		Cyprus	High
		Denmark	High
		Finland	High
		France	High
		Germany	High
		Greece	High
		Iceland	High
		Ireland	High
		Israel	High
		Italy	High
		Luxembourg	High
		Malta	High
		Monaco	High
		Netherlands	High
		Norway	High
		Portugal	High
		San Marino	High
		Spain	High
		Sweden	High
		Switzerland	High
		United Kingdom	High
Latin America and Caribbean			
	Andean Latin America		
		Bolivia	Lower middle
		Ecuador	Upper middle
		Peru	Upper middle
	Caribbean		
		Antigua and Barbuda	High
		The Bahamas	High
		Barbados	High
		Belize	Upper middle
		Bermuda	High
		Cuba	Upper middle

		Dominica	Upper middle
		Dominican Republic	Upper middle
		Grenada	Upper middle
		Guyana	Upper middle
		Haiti	Low
		Jamaica	Upper middle
		Puerto Rico	High
		Saint Kitts and Nevis	High
		Saint Lucia	Upper middle
		Saint Vincent and the Grenadines	Upper middle
		Suriname	Upper middle
		Trinidad and Tobago	High
		Virgin Islands, U.S.	High
	Central Latin America		
		Colombia	Upper middle
		Costa Rica	Upper middle
		El Salvador	Lower middle
		Guatemala	Lower middle
		Honduras	Lower middle
		Mexico	Upper middle
		Nicaragua	Lower middle
		Panama	Upper middle
		Venezuela	Upper middle
	Tropical Latin America		
		Brazil	Upper middle
		Paraguay	Upper middle
North Africa and Middle East			
	North Africa and Middle East		
		Afghanistan	Low
		Algeria	Upper middle
		Bahrain	High
		Egypt	Lower middle
		Iran	Upper middle
		Iraq	Upper middle
		Jordan	Lower middle
		Kuwait	High
		Lebanon	Upper middle
		Libya	Upper middle
		Morocco	Lower middle
		Palestine	Lower middle
		Oman	High
		Qatar	High
		Saudi Arabia	High
		Sudan	Lower middle
		Syria	Lower middle

		Tunisia	Lower middle
		Turkey	Upper middle
		United Arab Emirates	High
		Yemen	Lower middle
South Asia			
	South Asia		
		Bangladesh	Lower middle
		Bhutan	Lower middle
		India	Lower middle
		Nepal	Low
		Pakistan	Lower middle
Southeast Asia, East Asia, and Oceania			
	East Asia		
		China	Upper middle
		North Korea	Low
		Taiwan	High
	Oceania		
		American Samoa	Upper middle
		Cook Islands	High
		Federated States of Micronesia	Lower middle
		Fiji	Upper middle
		Guam	High
		Kiribati	Lower middle
		Marshall Islands	Upper middle
		Nauru	UPPER MIDDLE
		Niue	High
		Northern Mariana Islands	High
		Palau	High
		Papua New Guinea	Lower middle
		Samoa	Upper middle
		Solomon Islands	Lower middle
		Tokelau	High
		Tonga	Upper middle
		Tuvalu	Upper middle
		Vanuatu	Lower middle
	Southeast Asia		
		Cambodia	Lower middle
		Indonesia	Lower middle
		Laos	Lower middle
		Malaysia	Upper middle
		Maldives	Upper middle
		Mauritius	Upper middle
		Myanmar	Lower middle
		Philippines	Lower middle
		Sri Lanka	Lower middle

		Seychelles	High
		Thailand	Upper middle
		Timor-Leste	Lower middle
		Vietnam	Lower middle
Sub-Saharan Africa			
	Central Sub-Saharan Africa		
		Angola	Lower middle
		Central African Republic	Low
		Congo	Lower middle
		Democratic Republic of the Congo	Low
		Equatorial Guinea	Upper middle
		Gabon	Upper middle
	Eastern Sub-Saharan Africa		
		Burundi	Low
		Comoros	Low
		Djibouti	Lower middle
		Eritrea	Low
		Ethiopia	Low
		Kenya	Lower middle
		Madagascar	Low
		Malawi	Low
		Mozambique	Low
		Rwanda	Low
		Somalia	Low
		South Sudan	Low
		Tanzania	Low
		Uganda	Low
		Zambia	Lower middle
	Southern Sub-Saharan Africa		
		Botswana	Upper middle
		Lesotho	Lower middle
		Namibia	Upper middle
		South Africa	Upper middle
		Swaziland	Lower middle
		Zimbabwe	Low
	Western Sub-Saharan Africa		
		Benin	Low
		Burkina Faso	Low
		Cameroon	Lower middle
		Cape Verde	Lower middle
		Chad	Low
		Côte d'Ivoire	Lower middle
		The Gambia	Low
		Ghana	Lower middle
		Guinea	Low

		Guinea-Bissau	Low
		Liberia	Low
		Mali	Low
		Mauritania	Lower middle
		Niger	Low
		Nigeria	Lower middle
		Sao Tome and Principe	Lower middle
		Senegal	Low
		Sierra Leone	Low
		Togo	Low

Table S6. Global Burden of Disease location hierarchy. Each country/territory is assigned to one of 21 regions and 7 super regions in a hierarchical structure, based on their geographic locations and sociodemographic and epidemiological characteristics.

4.2.4 Model validation

We ran the full model (stacked ensemble plus ST-GPR) with full five-fold out-of-sample cross validation, holding out all of the data from 20% of the countries from each fold and predicting the antibiotic consumption for the held-out data. All data from each country was held out within the same fold, as there was very little temporal variation in the data; this provides a more rigorous test of the model's predictive validity. We then calculated the RMSE and r^2 for the data versus the out-of-sample prediction.

4.3 Types of antibiotics consumed

We investigated the suitability of spatial-temporal models to estimate a) consumption for each antibiotic class at ATC level 3, b) consumption for select ATC level 4 classes and c) consumption of antibiotics according to AWaRe category[10]. We were unable to identify covariates to satisfactorily explain the spatial-temporal patterns of consumption for these subgroups, and could not identify clear spatial trends in consumption. It was therefore deemed inappropriate to model classes of antibiotics (at the ATC level 3 or 4) or AWaRe categorisation.

To assess the spatial and temporal trends in consumption of different ATC level 3 class antibiotics, and select ATC level 4 class antibiotic, we calculated the proportion of the total J01 class antibiotics consumed for each class of antibiotic by country and year from the applicable cleaned data, pre imputation of missing values, (all data sources combined for ATC level 3 and solely IQVIA for ATC level 4 estimates) and applied these proportions to the modelled estimates of total (J01) antibiotic consumption. For country-years with no data on ATC level 3 or 4 class antibiotic consumption, we calculated these proportion for the corresponding GBD region, and applied these numbers to the estimated total antibiotic consumption for each country-year.

The proportion of antibiotics in each AWaRe category (Access, Watch or Reserve)[10] were calculated from the IQVIA dataset and plotted and a map to allow visual comparison of consumption between countries.

Supplementary Results

1. Antibiotic usage model results

1.1 Model results

We took 1000 draws of the model posterior for each 5 x 5km pixel in our study area for each year, 2000-2018. These were then aggregated to the district (admin 2), state (admin 1) and national (admin 0) levels. (Figure S13) We are primarily showing the district level estimates as this is often the spatial resolution at which healthcare policy is administrated and allows identification of fine spatial trends without focussing on too small populations.

Our modelling strategy allowed us to quantify uncertainty within the model. We calculated the relative uncertainty of our estimates for each district (the ratio of the uncertainty intervals to the proportion of antibiotic usage) and display this in relation to the proportion of antibiotic usage (Figure S11). A wide range in antibiotic usage (32-75%) was estimated in Latin America and the Caribbean; however, uncertainty estimates in this region were high due to a paucity of data. Uncertainty was relatively low in India and Central Asia, where antibiotic usage was high, but uncertainty was high in Indonesia, Philippines and Papua New Guinea where there is low antibiotic usage. In sub-Saharan Africa, our estimates have low uncertainty in many countries (Ethiopia, South Sudan, Central African Republic, Nigeria, Benin, Côte d'Ivoire), however, uncertainty was high in locations lacking data such as Botswana, Mozambique, and Niger (Figure S11). Figure S12 shows the results for the (a) 2.5 percentile, (b) the mean, and (c) the 97.5th percentile of the 1000 draws of our model posterior.

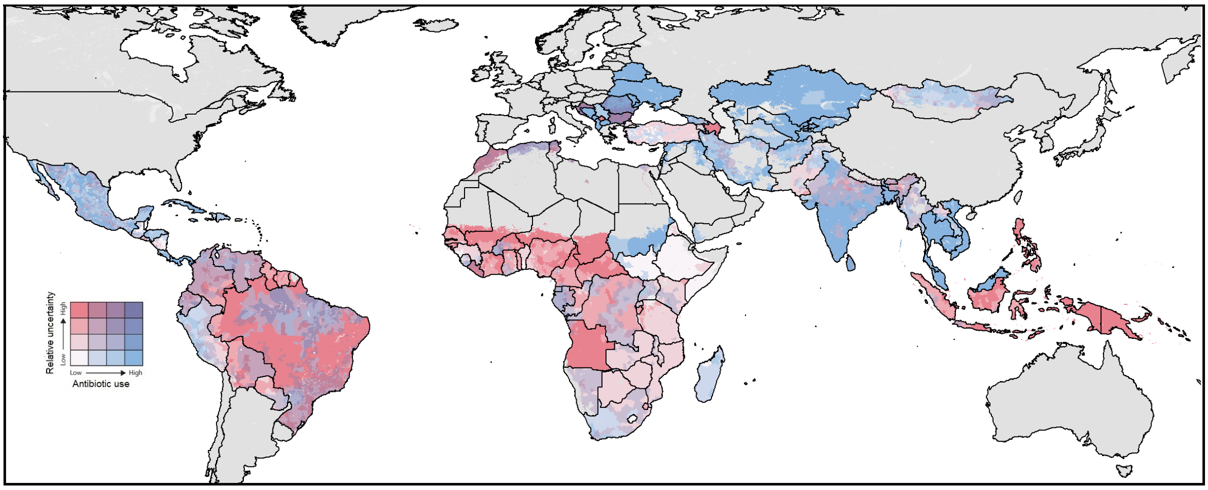

Figure S11: Relative uncertainty of antibiotic usage for 2018. Estimates are shown for level two administrative divisions in 2018. Relative uncertainty was defined as the ratio of the 95% uncertainty intervals to the proportion of antibiotic use. Data were coloured based on the quartile of the proportion of antibiotic use and the quartile of relative uncertainty to which they belonged. Pixels (1 x 1km) with populations less than 10 persons are masked in grey.

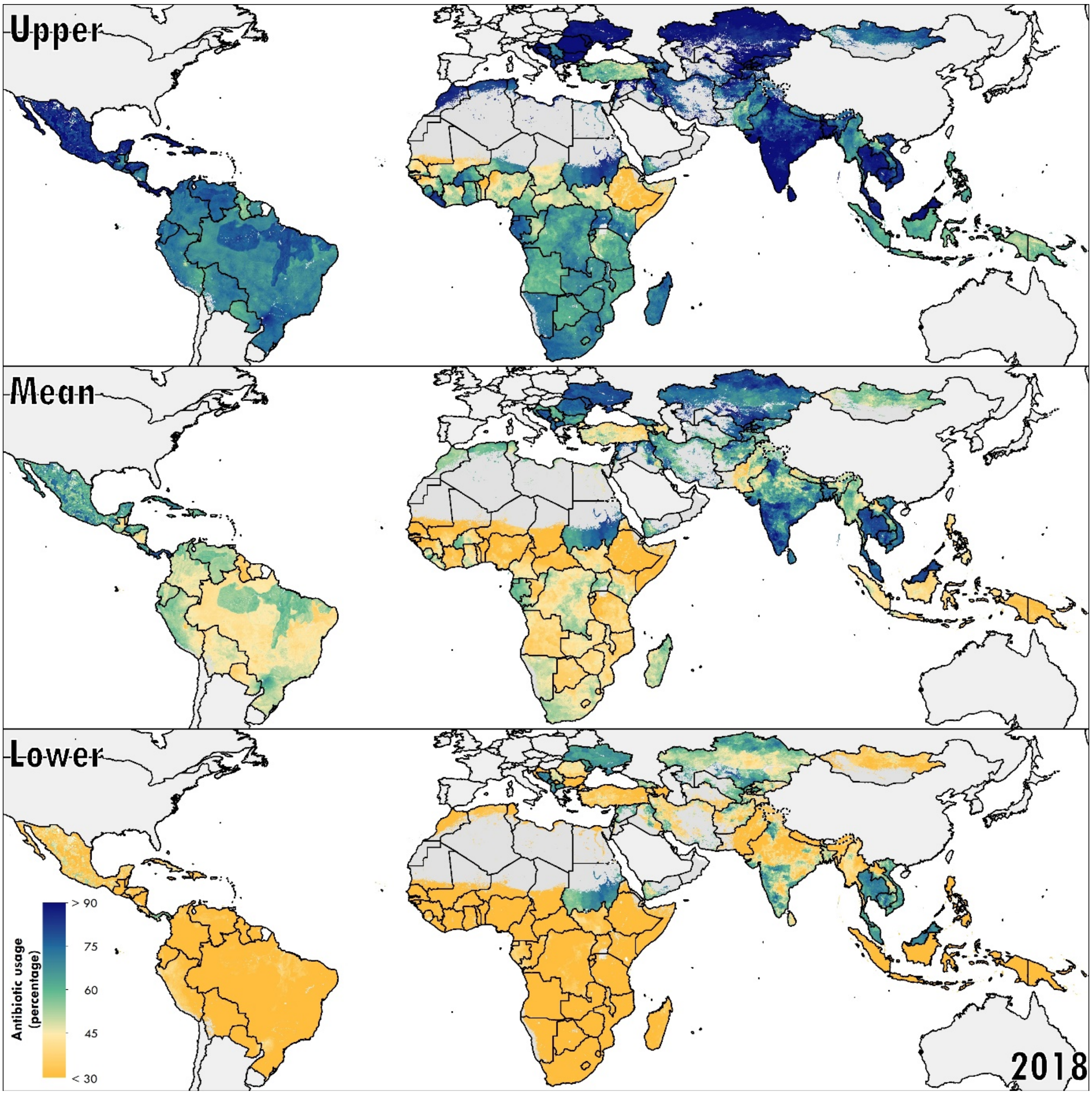

Figure S12. Estimates of the proportion of antibiotic usage in LMICs for 2018 with the mean and upper and lower 95% uncertainty intervals. a) Estimates of the upper 95% uncertainty value; b) mean estimates; c) estimates of the lower 95% uncertainty value. 1 x 1 km pixels with a population of less than 10 people have been masked.

Modelling antibiotic usage at the pixel level allows to present the results at varying spatial aggregations (Figure S13). Large spatial disparities are evident in India, Pakistan, DRC and Nigeria and national estimates can mask these patterns in antibiotic use. However, there is little spatial variation in many countries, possibly indicating the drive for caregivers to seek treatments when the child is suffering from LRI, in spite of potential financial and healthcare access limitations.

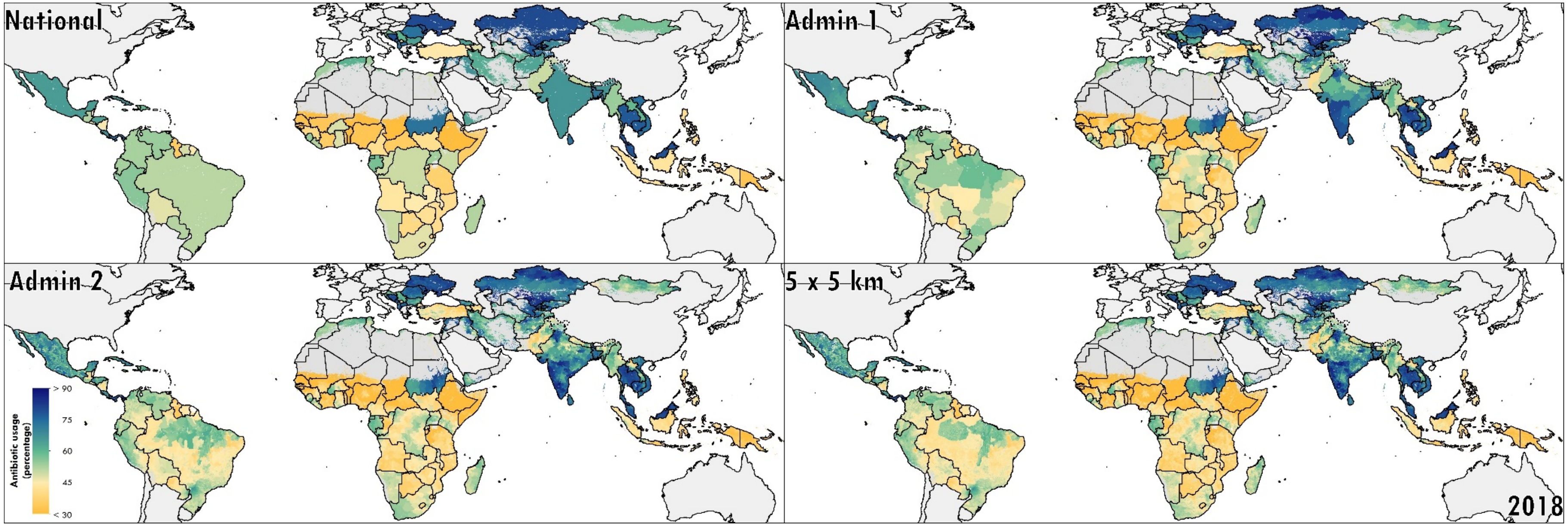

Figure S13. Estimates of the proportion of antibiotic usage in LMICs for 2018 at varying spatial aggregations. a) national estimates; b) estimates for level 1 administrative divisions; c) estimates for level 2 administrative divisions; d) estimates for 5x5km pixels. All estimates are for 2018.

1.2 Model validation

We calculated validation metrics between the out-of-sample predictions of the model, aggregated to the national level, and the total proportion of antibiotic use from each survey in the input dataset. We used the root mean square error (RMSE) to indicate how far from the data the model estimates lay, and the r^2 to show the correlation between the model estimates and the input data. The full model was correlated to the input data with an r^2 of 0.53 and had an RMSE of 0.14 (Figure S14); the predictive validity varied by region (Table S7). The model performed best in the African regions, South America and the Balkans and Caucasus but struggled for predictive validity in Asia. This is partially due to a paucity of survey data in these regions (Figure S15), but also indicates that complex drivers of antibiotic use might not be captured by our covariates or spatial-temporal trends.

Region	In sample		Out of sample	
	RMSE	Correlation (r^2)	RMSE	Correlation (r^2)
**OVERALL**	**0.03**	**0.99**	**0.14**	**0.53**
Balkans & Caucasus	0.02	1.00	0.14	0.73
Central America & Caribbean	0.02	1.00	0.15	-0.09
Central Asia	0.03	0.98	0.09	0.46
Central sub-Saharan Africa	0.02	0.98	0.07	0.70
Eastern & Southern sub-Saharan Africa	0.01	1.00	0.11	0.54
Horn of Africa	0.01	1.00	0.09	0.89
Malay & Oceania	0.01	0.99	0.11	-0.80
Middle East	0.02	0.99	0.11	-0.05
North Africa	0.01	1.00	0.09	0.21
South America	0.02	0.97	0.07	0.14
South Asia	0.05	0.99	0.24	-0.20
Southeast Asia	0.03	0.99	0.23	-0.44
Western sub-Saharan Africa	0.02	0.99	0.11	0.24

Table S7. In- and out-of-sample metrics for the five-fold cross validation models on antibiotic use

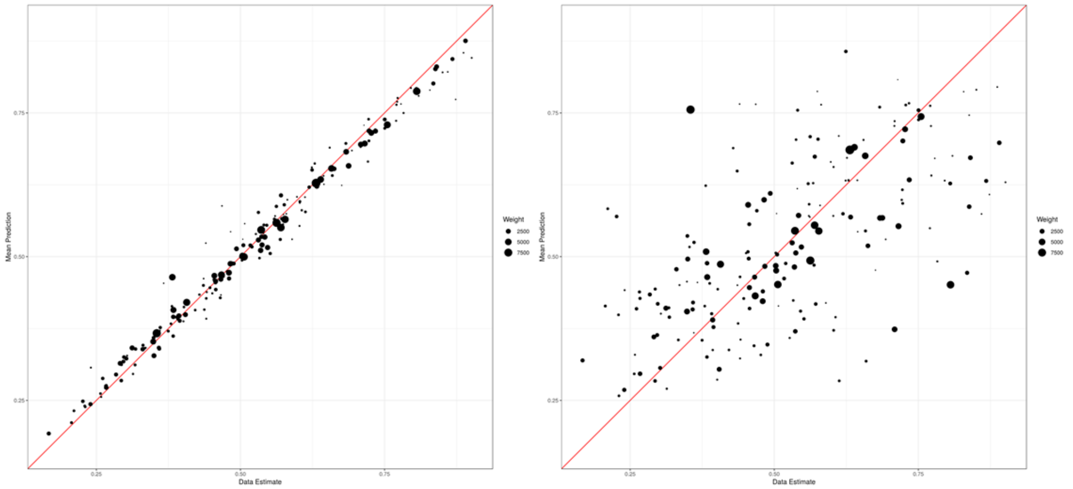

Figure S14. Validation plots for the model of antibiotic usage. a) Scatter plot representing the mean proportion of antibiotic use for each survey, at the national level, against the in-sample predicted proportion of antibiotic use for that country-year; b) Scatter plots of the mean proportion of antibiotic use for each survey, at the national level, against the out-of-sample predicted proportion of antibiotic use for that country based on five-fold cross validation

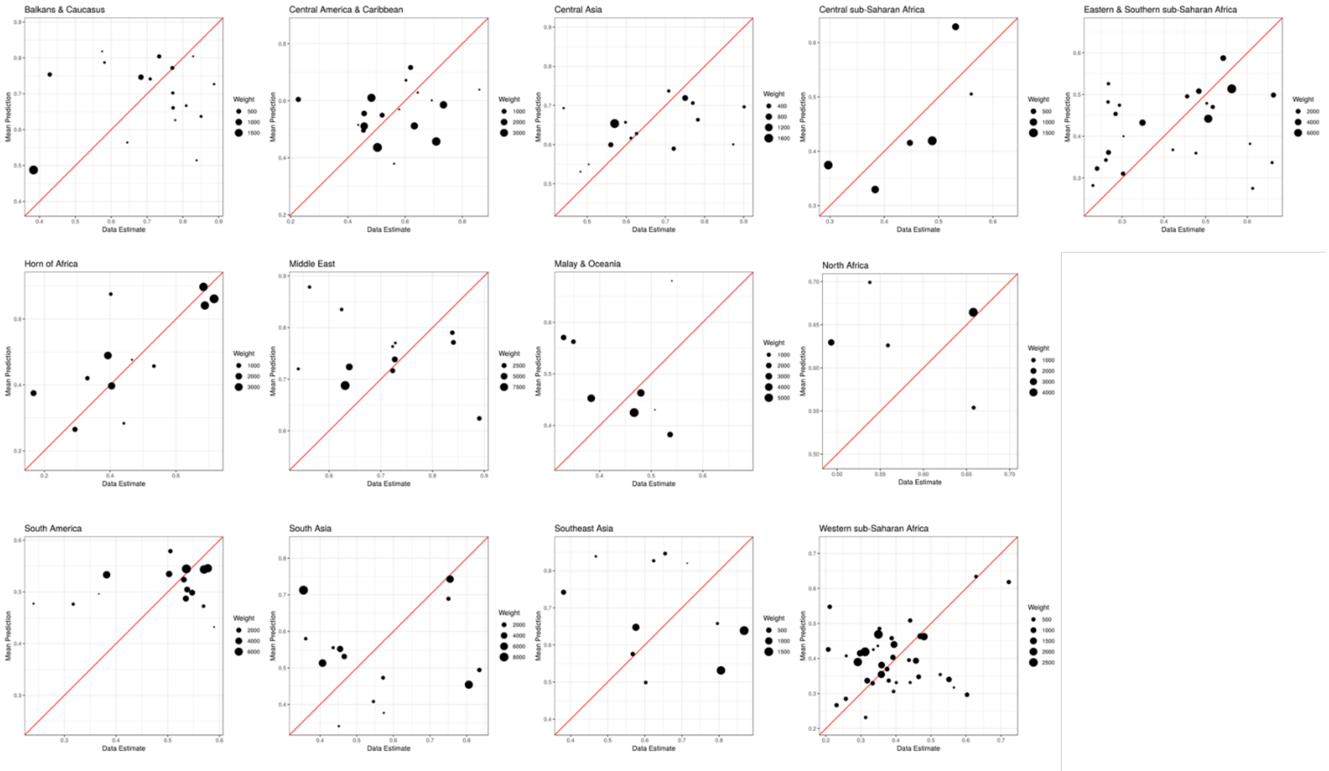

Figure S15. Validation plots for the model of antibiotic use for each modelling region. Each plot shows the mean proportion of antibiotic use from each survey, at the national level, against the out-of-sample predicted proportion of antibiotic use for that country-year based on five-fold cross-validation. a) Balkans & Caucasus; b) Central America & Caribbean; c) Central Asia; d) Central sub-Saharan Africa; e) Eastern & Southern sub-Saharan Africa; f) Horn of Africa; g) Malay & Oceania; h) Middle East; i) North Africa; j) South America; k) South Asia; l) Southeast Asia; m) Western sub-Saharan Africa.

2. Global antibiotic consumption model results

2.1 Model results

We predicted the total antibiotic consumption for 204 countries from 2000 to 2018, five-yearly estimates of antibiotic consumption are displayed in Figure S16. Our final estimates for LMICs were the mean of 1000 draws of a GPR with the 95% uncertainty estimates calculated from the GPR draws. These were then combined with the multiple imputation results for HICs and the DDD per 1000 per day calculated. As the final results are a combination of methods, uncertainty is only displayed for the LMICs model. Due to no uncertainty intervals being available for the HIC model, when aggregating estimates to the regional, super-regional, or global level the upper and lower 95% uncertainty intervals are calculated using the single estimate of antibiotic consumption for HICs, in addition to the uncertainty intervals from the LMIC model.

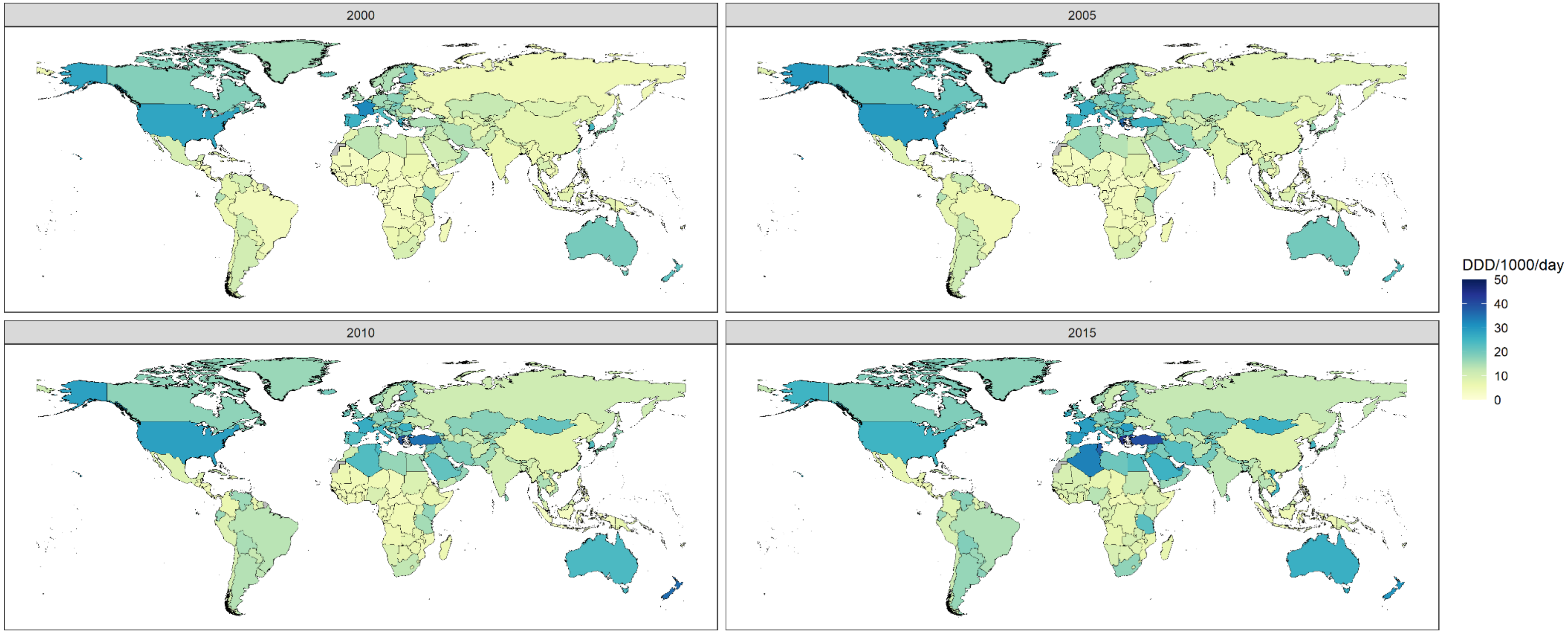

Figure S16. Five yearly global estimates of total antibiotic consumption rates from 2000 to 2015. Estimates of antibiotic consumption rates in defined daily doses (DDDs) per 1000 population per day are provided for 204 countries, for the years 2000; 2005; 2010; and 2015.

We also present consumption according to classes of antibiotics. Consumption of different classes varied greatly between countries, with consumption of penicillins the highest and amphenicols and aminoglycosides the lowest (Figure S17). Consumption of penicillins (J01C) and other beta-lactams (J01D) increased greatly throughout the study period, whilst macrolides, lincosamides and streptogramins (J01F) and quinolones (J01M) increased modestly, amphenicols (J01B) and aminoglycosides (J01G) remained stable and of sulfonamides and trimethoprim (J01E) decreased (Figure S18).

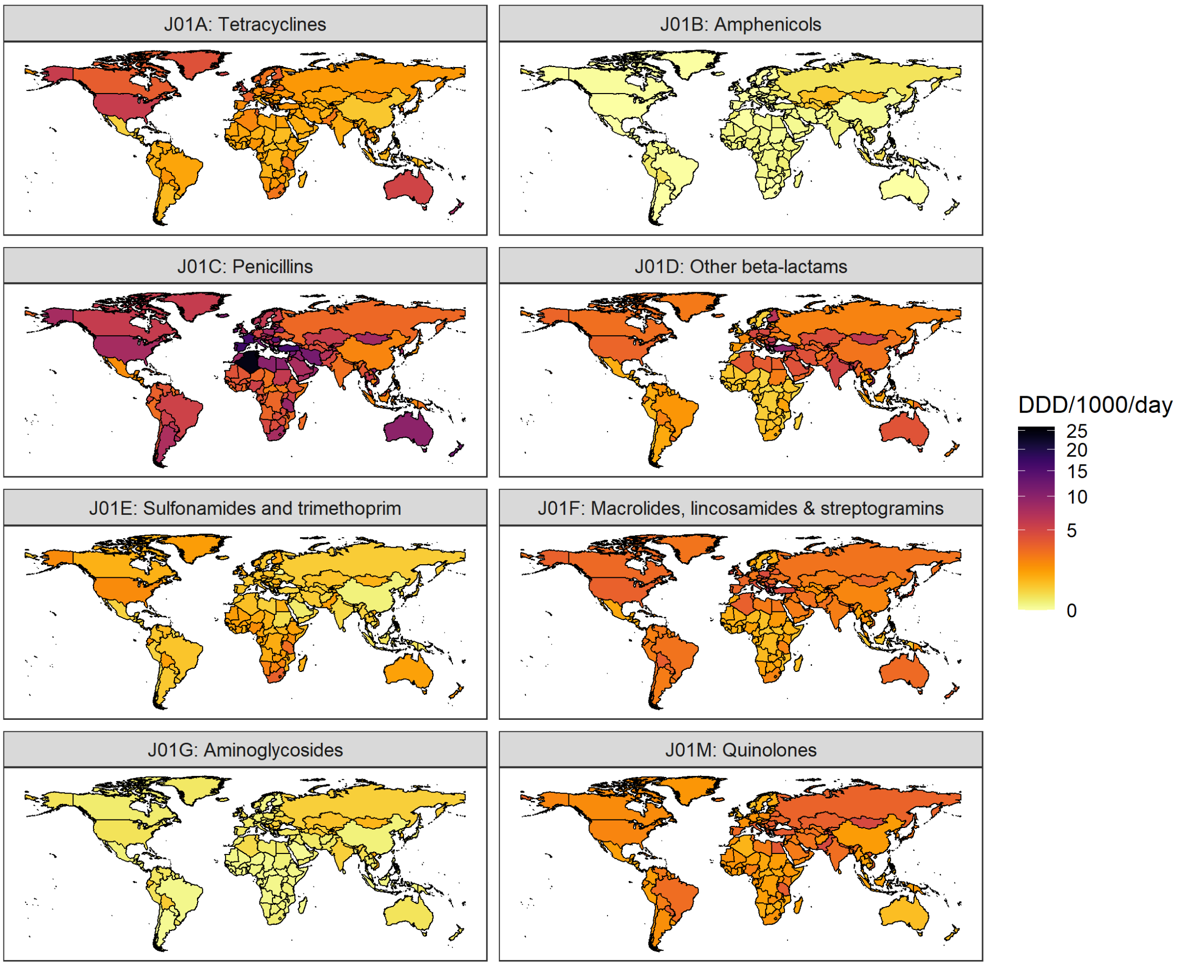

Figure S17: Global antibiotic consumption by class (ATC level 3) for 2018. The proportions of consumption of each antibiotic class were calculated from raw IQVIA, WHO and ESAC-Net data and applied to the modelled total antibiotic consumption for 204 countries and expressed as DDDs per 1000 population per day on a square root transformed scale.

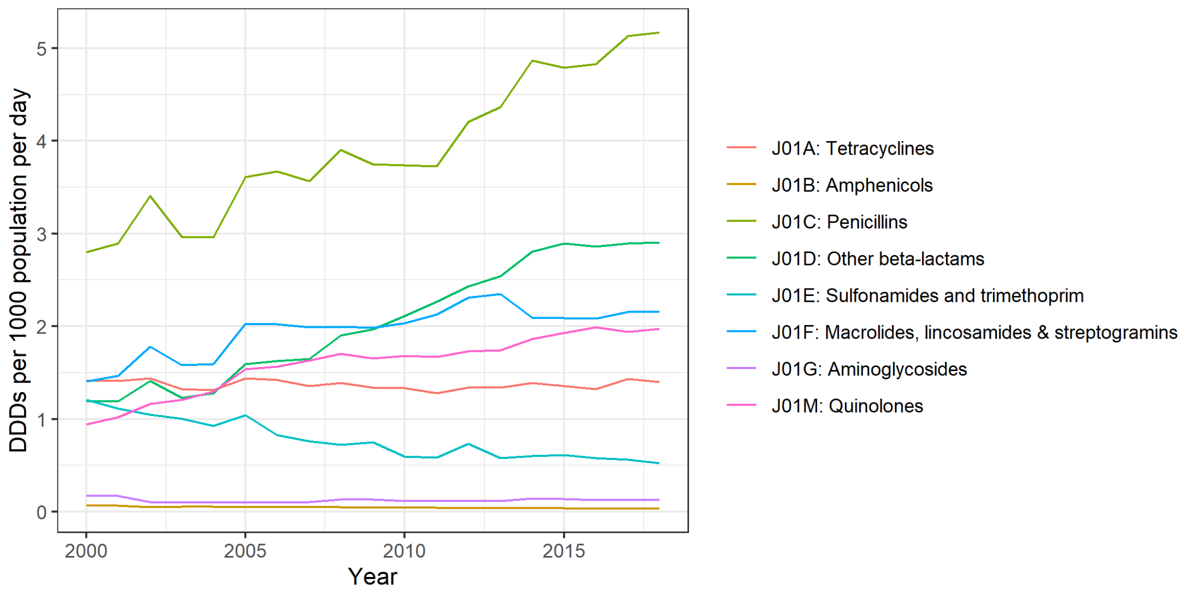

Figure S18: Temporal trends in global antibiotic consumption rates by class (ATC level 3) for 2000-2018. The proportions of consumption of each antibiotic class were calculated from raw IQVIA, WHO and ESAC-Net data and applied to the modelled total antibiotic consumption for each country and expressed as DDDs per 1000 population per day.

We present antibiotic consumption of key ATC level 4 classes of antimicrobials for selected important GBD super-regions.

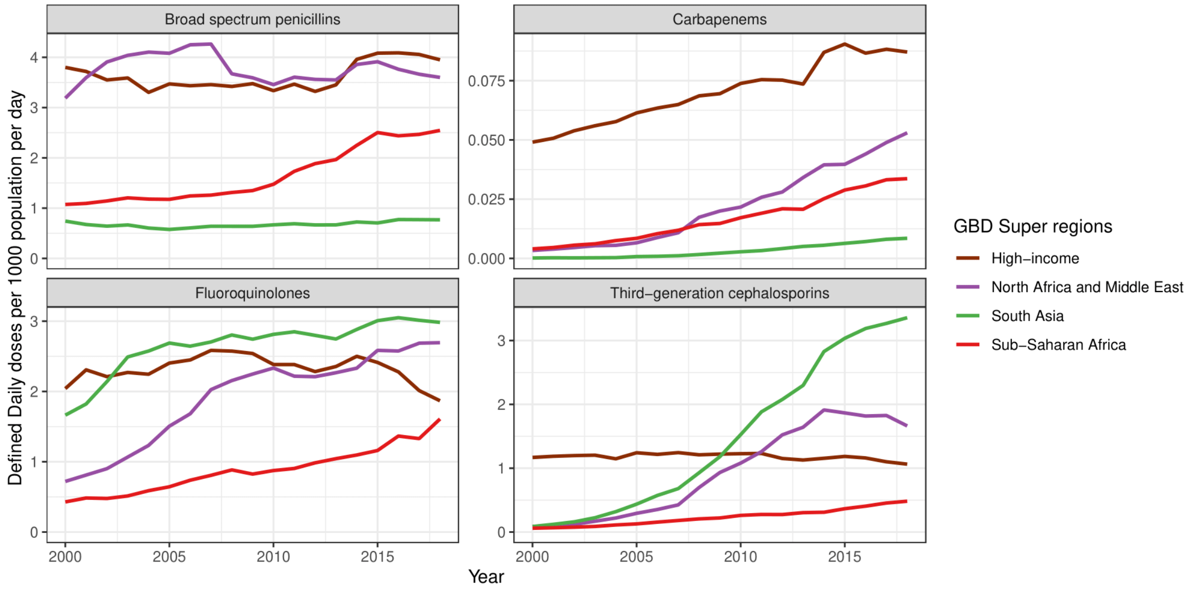

Figure S19: Temporal trends in consumption of selected antibiotic classes (ATC level 4), displayed for four selected GBD super regions. The proportion of each antibiotic class (ATC level 4) was calculated from the raw IQVIA dataset and applied to the modelled estimates of total antibiotic consumption for each country and aggregated to the High-Income; North Africa and the Middle East; South Asia; and Sub-Saharan Africa super regions. Estimates are displayed for: a) broad spectrum penicillins, J01CA; b) carbapenems, J01DH; c) Fluoroquinolones, J01MA; and d) third generation cephalosporins, J01DD**.**

For similar reasons, we also deemed a spatial model unsuitable when investigating consumption by AWaRe categories. Using the raw IQVIA data, the proportions of total antibiotic consumption for each category is shown in Figure S20.

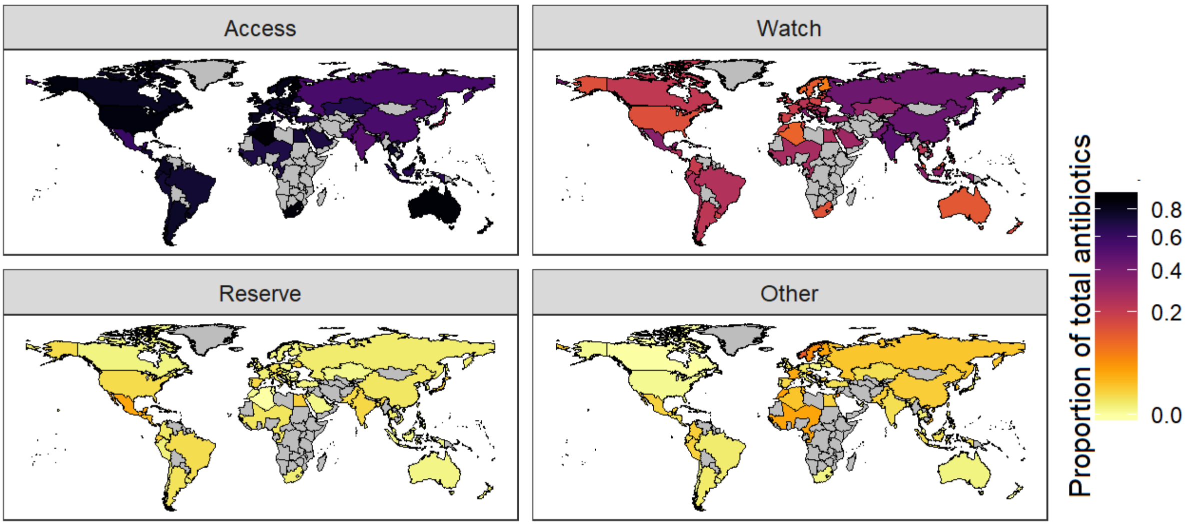

Figure S20. Proportion of total antibiotics consumed by AWaRe categories for 2018. Data from IQVIA; antibiotics are classified as 'Access', 'Watch', 'Reserve' or 'Other' based on the WHO Essential Medicines list[10] and expressed as a proportion of the total antibiotic consumption.

2.3 Model validation

The full model for antibiotic consumption in LMICs was run with five-fold cross validation and the RMSE and r^2 between the input data and the model estimates calculated. Overall the model had relatively good predictive validity with an r^2 of 0.57 and an RMSE of 1672 (DDD per 1000 population) between the input data and the out-of-sample estimates.

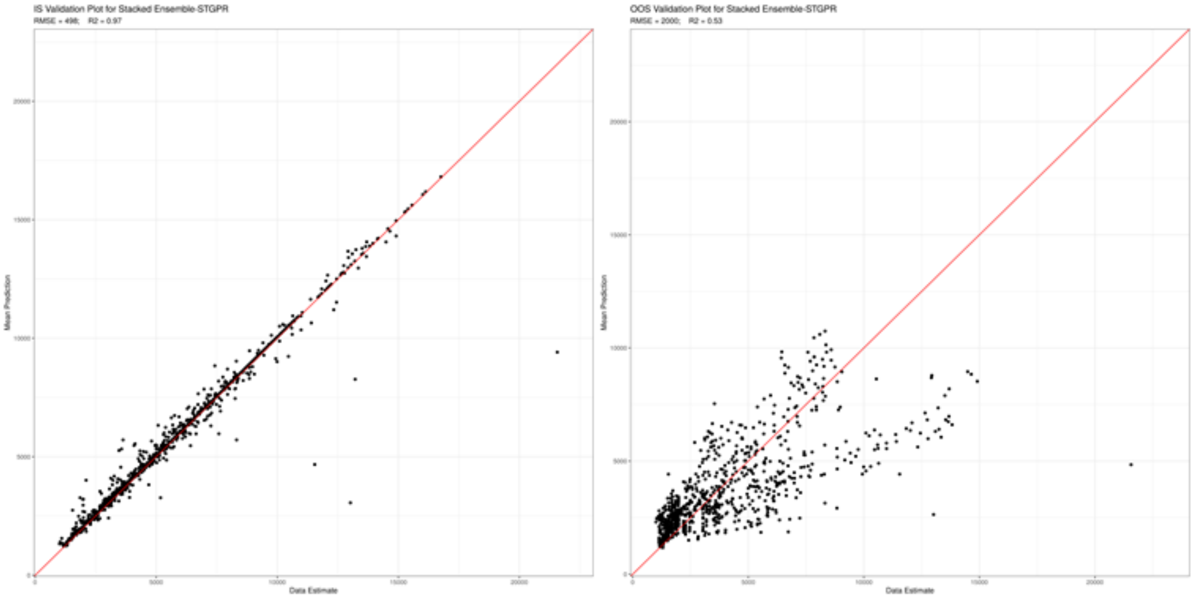

Figure S21. Validation plots for the model of antibiotic consumption. a) Scatter plot presenting the antibiotic consumption of J01 antibiotics in DDDs/1000/year for each country-year, against the in-sample predicted antibiotic consumption for that country-year; b) Scatter plot of the antibiotic consumption of J01 antibiotics in DDDs/1000/year for each country-year, against the out-of-sample predicted antibiotic consumption for that country based on five-fold cross validation

2.4 Impact of updated daily defined dose values

In 2019 the WHO updated the DDD values for nine key antimicrobials, including some of the most highly utilised broad spectrum antibiotics – ampicillin and amoxicillin[6,7]. This increased the DDD for parenteral ampicillin from 2g to 6g, parenteral amoxicillin from 1g to 3g and oral amoxicillin from 1g to 1.5g. These alterations mean that a given quantity in kg of one of these antibiotics now represents a lower number of DDDs. For example, 1kg of parenteral ampicillin would have been 500 DDDs based on the previous (historic) DDD value, but this now only amounts to 167 DDDs when using the updated 2019 DDD value. To assess the impact of these changes on our estimates we used the IQVIA dataset and calculated the total DDDs per 1000 population per day for HICs and LMICs for each year, 2000-2018, based on a) the updated 2019 DDD values, and b) the previous (historic) DDD values.

In 2018, the change in DDD values had a large impact on the rate of antibiotic consumption in HICs, lowering the DDD/1000/day from 24.4 DDD/1000/day (when using the historic values) to 20.5 DDD/1000/day when using the updated values. Interestingly, in LMICs, this had no impact on the rates of antibiotic consumption (Table S8).

Year	Using the 2019 DDD values				Using the historic (pre-2019) DDD values			
	LMIC		HIC		LMIC		HIC	
	DDDs	DDD/1000/DAY	DDDs	DDD/1000/DAY	DDDs	DDD/1000/DAY	DDDs	DDD/1000/DAY
2000	7,941,273,121	5.3	8,180,210,981	20.5	8,782,752,352	5.8	9,317,706,888	23.4
2001	8,135,122,711	5.3	8,423,974,386	21.0	9,049,989,370	5.9	9,590,958,821	23.9
2002	8,632,942,591	5.6	8,267,659,921	20.5	9,625,730,124	6.2	9,432,438,766	23.4
2003	8,957,001,860	5.7	8,455,591,732	20.8	9,974,750,377	6.4	9,680,632,834	23.9
2004	9,355,688,655	5.9	8,177,810,129	20.0	10,423,804,696	6.6	9,367,309,339	22.9
2005	10,706,204,184	6.7	8,763,631,276	21.3	11,851,729,781	7.4	10,038,898,121	24.4
2006	11,035,958,469	6.8	8,729,105,068	21.1	12,228,980,551	7.5	10,025,765,573	24.2
2007	11,307,828,042	6.9	8,857,180,354	21.2	12,559,914,143	7.6	10,203,135,664	24.4
2008	12,509,263,703	7.5	8,961,369,504	21.3	13,823,260,463	8.3	10,369,383,530	24.6
2009	13,295,998,894	7.9	9,206,162,654	21.7	14,721,185,039	8.8	10,659,559,946	25.1
2010	14,421,006,630	8.5	9,283,336,460	21.7	15,967,073,294	9.4	10,736,314,874	25.1
2011	15,774,694,530	9.2	9,537,837,951	22.2	17,558,520,419	10.2	11,048,105,112	25.7
2012	18,258,322,653	10.5	9,056,231,163	21.0	20,358,120,918	11.7	10,527,788,978	24.4
2013	18,288,340,383	10.4	9,136,754,953	21.0	20,482,247,939	11.7	10,675,488,193	24.6
2014	19,754,398,040	16.0	9,382,566,526	21.6	17,705,278,173	14.3	11,102,186,075	25.5
2015	21,014,630,910	16.8	9,575,548,330	21.9	18,823,195,548	15.0	11,365,282,682	26.0
2016	22,006,487,057	17.3	9,502,828,938	21.6	19,859,085,212	15.6	11,298,381,362	25.7
2017	21,492,408,297	16.7	9,360,217,484	21.2	19,559,778,903	15.2	11,143,202,033	25.2
2018	22,133,931,886	17.0	9,061,093,060	20.5	20,455,854,131	15.7	10,805,800,040	24.4

Table S8: Total DDDs and DDD/1000/day in HICs and LMICs for 2000-2018 based on calculations using the updated 2019 DDD values compared to the historic (pre-2019) DDD values [5,7]

Gather Checklist

This study was conducted in line with the Guidelines for Accurate and Transparent Health Estimates Reporting (GATHER) (Table S9).[20]

Item #	Checklist item	Reported on page #
**Objectives and funding**		
**1**	Define the indicator(s), populations (including age, sex, and geographic entities), and time period(s) for which estimates were made.	10,12
**2**	List the funding sources for the work.	15
**Data Inputs**		
For all data inputs from multiple sources that are synthesized as part of the study:		
**3**	Describe how the data were identified and how the data were accessed.	10,12,13
**4**	Specify the inclusion and exclusion criteria. Identify all ad-hoc exclusions.	S36
**5**	Provide information on all included data sources and their main characteristics. For each data source used, report reference information or contact name/institution, population represented, data collection method, year(s) of data collection, sex and age range, diagnostic criteria or measurement method, and sample size, as relevant.	12-13, S6-S24
**6**	Identify and describe any categories of input data that have potentially important biases (e.g., based on characteristics listed in item 5).	23,24
For data inputs that contribute to the analysis but were not synthesized as part of the study:		
**7**	Describe and give sources for any other data inputs.	13, S27, S40
For all data inputs:		
**8**	Provide all data inputs in a file format from which data can be efficiently extracted (e.g., a spreadsheet rather than a PDF), including all relevant meta-data listed in item 5. For any data inputs that cannot be shared because of ethical or legal reasons, such as third-party ownership, provide a contact name or the name of the institution that retains the right to the data.	S6-S24, S27, S34, S35
**Data analysis**		
**9**	Provide a conceptual overview of the data analysis method. A diagram may be helpful.	S3
**10**	Provide a detailed description of all steps of the analysis, including mathematical formulae. This description should cover, as relevant, data cleaning, data pre-processing, data adjustments and weighting of data sources, and mathematical or statistical model(s).	10-15, S4-S49
**11**	Describe how candidate models were evaluated and how the final model(s) were selected.	S30, S53, S57-58
**12**	Provide the results of an evaluation of model performance, if done, as well as the results of any relevant sensitivity analysis.	S53, S57-S58
**13**	Describe methods for calculating uncertainty of the estimates. State which sources of uncertainty were, and were not, accounted for in the uncertainty analysis.	S30, S50-S51
**14**	State how analytic or statistical source code used to generate estimates can be accessed.	25
**Results and Discussion**		
**15**	Provide published estimates in a file format from which data can be efficiently extracted.	25
**16**	Report a quantitative measure of the uncertainty of the estimates (e.g. uncertainty intervals).	25, S30, S50
**17**	Interpret results in light of existing evidence. If updating a previous set of estimates, describe the reasons for changes in estimates.	20-21
**18**	Discuss limitations of the estimates. Include a discussion of any modelling assumptions or data limitations that affect interpretation of the estimates.	23-24

Table S9: GATHER Checklist: A checklist identifying each point of the Guidelines for Accurate and Transparent Health Estimates Reporting and whether they are covered in this study.

References

1. Lloyd CT, Sorichetta A, Tatem AJ. High resolution global gridded data for use in population studies. *Scientific Data* 2017; **4**(1): 170001.
2. Golding N, Burstein R, Longbottom J, et al. Mapping under-5 and neonatal mortality in Africa, 2000-2015: a baseline analysis for the Sustainable Development Goals. *Lancet* 2017; **390**(10108): 2171-82.
3. Bhatt S, Cameron E, Flaxman SR, Weiss DJ, Smith DL, Gething PW. Improved prediction accuracy for disease risk mapping using Gaussian process stacked generalization. *J R Soc Interface* 2017; **14**(134): 20170520.
4. Lindgren F, Rue H, Lindström J. An explicit link between Gaussian fields and Gaussian Markov random fields: the stochastic partial differential equation approach. *Journal of the Royal Statistical Society: Series B (Statistical Methodology)* 2011; **73**(4): 423-98.
5. WHO Collaborating Centre for Drug Statistics Methodology. Guidelines for ATC classification and DDD assignment 2020. Oslo, Norway, 2019.
6. WHO Collaborating Centre for Drug Statistics Methodology. ATC/DDD Index 2020. 2019. https://www.whocc.no/atc_ddd_index/ (accessed 8th June 2020).
7. WHO Collaborating Centre for Drug Statistics Methodology. DDD alterations from 2005-2020. 2020. https://www.whocc.no/atc_ddd_alterations__cumulative/ddd_alterations/ (accessed 8th June 2020).
8. van Buuren S, Groothuis-Oudshoorn K. mice: Multivariate Imputation by Chained Equations in R. *J Stat Softw* 2011; **45**(3): 1-67.
9. Azur MJ, Stuart EA, Frangakis C, Leaf PJ. Multiple imputation by chained equations: what is it and how does it work? *Int J Methods Psychiatr Res* 2011; **20**(1): 40-9.
10. World Health Organization. World Health Organization model list of essential medicines, 21st List. Geneva, 2019.
11. World Health Organization. WHO report on surveillance of antibiotic consumption: 2016-2018 early implementation. Geneva: World Health Organization, 2018.
12. The Center for Disease Dynamics EaP. ResistanceMap. 2020. https://resistancemap.cddep.org/AntibioticUse.php (accessed 12th June 2020).
13. Togoobaatar G, Ikeda N, Ali M, et al. Survey of non-prescribed use of antibiotics for children in an urban community in Mongolia. *Bull World Health Organ* 2010; **88**(12): 930-6.
14. Nakajima R, Takano T, Urnaa V, Khaliun N, Nakamura K. Antimicrobial use in a country with insufficient enforcement of pharmaceutical regulations: A survey of consumption and retail sales in Ulaanbaatar, Mongolia. *South Med Rev* 2010; **3**(1): 19-23.
15. Dorj G, Salvesen Blix H, Sunderland B, et al. Antibiotic utilization trends in two state hospitals of Mongolia from 2013 to 2017. *BioMed Research International* 2019; **2019**.
16. European Centre for Disease Prevention and Control. Antimicrobial consumption database (ESAC-Net). 2019. https://www.ecdc.europa.eu/en/antimicrobial-consumption/surveillance-and-disease-data/database (accessed 23rd December 2019).
17. Norris P, Nguyen HA. Consumption of antibiotics in a small Pacific island nation: Samoa. *Pharm Pract* 2007; **5**(1): 36-41.
18. Mitema ES, Kikuvi GM. Surveillance of the overall use of antimicrobial drugs in humans over a 5 year period (1997-2001) in Kenya. *J Antimicrob Chemother* 2004; **54**(5): 966-7.
19. GBD 2016 Risk Factors Collaborators. Global, regional, and national comparative risk assessment of 84 behavioural, environmental and occupational, and metabolic risks or clusters of risks, 1990-2016: a systematic analysis for the Global Burden of Disease Study 2016. *Lancet* 2017; **390**(10100): 1345-422.
20. Stevens GA, Alkema L, Black RE, et al. Guidelines for Accurate and Transparent Health Estimates Reporting: the GATHER statement. *Lancet* 2016; **388**(10062): e19-e23.
